# The *icmF3* locus is involved in multiple adaptation- and virulence-related characteristics in *Pseudomonas aeruginosa* PAO1

**DOI:** 10.3389/fcimb.2015.00070

**Published:** 2015-10-01

**Authors:** Jinshui Lin, Juanli Cheng, Keqi Chen, Chenghao Guo, Weipeng Zhang, Xu Yang, Wei Ding, Li Ma, Yao Wang, Xihui Shen

**Affiliations:** ^1^State Key Laboratory of Crop Stress Biology for Arid Areas and College of Life Sciences, Northwest A&F UniversityYangling, China; ^2^Life Sciences Department, Yuncheng UniversityYuncheng, China

**Keywords:** *Pseudomonas aeruginosa*, IcmF, type VI secretion system, virulence, environmental adaptation

## Abstract

The type VI secretion system (T6SS) is widely distributed in Gram-negative bacteria. Three separate T6SSs called H1-, H2-, and H3-T6SS have been discovered in *Pseudomonas aeruginosa* PAO1. Recent studies suggest that, in contrast to the H1-T6SS that targets prokaryotic cells, H2- and H3-T6SS are involved in interactions with both prokaryotic and eukaryotic cells. However, the detailed functions of T6SS components are still uncharacterized. The intracellular multiplication factor (IcmF) protein is conserved in type VI secretion systems (T6SS) of all different bacterial pathogens. Bioinformatic analysis revealed that IcmF3 in *P. aeruginosa* PAO1 is different from other IcmF homologs and may represent a new branch of these proteins with distinct functions. Herein, we have investigated the function of IcmF3 in this strain. We have shown that deletion of the *icmF3* gene in *P. aeruginosa* PAO1 is associated with pleiotropic phenotypes. The *icmF3* mutant has variant colony morphology and an hypergrowth phenotype in iron-limiting medium. Surprisingly, this mutant is also defective for the production of pyoverdine, as well as defects in swimming motility and virulence in a *C. elegans* worm model. The *icmF3* mutant exhibits higher conjugation frequency than the wild type and increased biofilm formation on abiotic surfaces. Additionally, expression of two phenazine biosynthetic loci is increased in the *icmF3* mutant, leading to the overproduction of pyocyanin. Finally, the mutant exhibits decreased susceptibility to aminoglycosides such as tobramycin and gentamicin. And the detected phenotypes can be restored completely or partially by trans complementation of wild type *icmF3* gene. The pleiotropic effects observed upon *icmF3* deletion demonstrate that *icmF3* plays critical roles in both pathogenesis and environmental adaptation in *P. aeruginosa* PAO1.

## Introduction

*Pseudomonas aeruginosa* is a Gram-negative opportunistic pathogen that is responsible for a wide range of human diseases, including septicaemia, pneumonia, and other serious infections. One possible explanation for its virulence is that *P. aeruginosa* is equipped with nearly all the different types of secretion machinery except for the T4SS (Bleves et al., 2010). Notably, three distinct and conserved type VI secretion system (T6SS) loci, H1-, H2-, and H3-T6SS, are present in *P. aeruginosa* genomes (Mougous et al., 2006). H1-T6SS has been widely studied and is known to deliver several toxic effectors into target bacteria, thus providing a major fitness advantage for *P. aeruginosa* (Russell et al., 2014). Recent studies have suggested that, in contrast to H1-T6SS targeting prokaryotic cells, H2- and H3-T6SS are involved in interactions with both prokaryotic and eukaryotic cells through the PldA and PldB trans-kingdom effectors. PldA and PldB are active in the bacterial periplasm and they exert antibacterial activity. Furthermore, PldA and PldB can also facilitate intracellular invasion of host eukaryotic cells by activating the phosphatidylinositol 3-kinase (PI3K)/Akt signaling pathway (Lesic et al., 2009; Sana et al., 2012; Russell et al., 2013; Jiang et al., 2014). It has been recently shown that the H2-T6SS machinery promotes *P. aeruginosa* internalization in eukaryotic host cells through the evolved VgrG2b effector (Sana et al., 2015). However, the mechanism of H2- and H3-T6SS action warrants further investigation.

Although the exact function of most T6SS proteins remains unknown, 13 core components are conserved in most T6SSs. These include proteins such as hemolysin-coregulated protein (Hcp), valine-glycine repeat protein (VgrG), AAA^+^-family ATPase (ClpV), intracellular multiplication protein (IcmF) and organelle trafficking protein (DotU) (Silverman et al., 2012). IcmF was first identified in *Legionella pneumophila*, where it is also a component of the T4SS. The IcmF component of the T4SS machinery of *L. pneumophila* is an accessory protein since it can be dispensable (Sexton et al., 2004). Structurally, IcmF is anchored to the inner membrane through three transmembrane (TM) domains, whereas the bulk of IcmF is located in the periplasm. A cytoplasmic domain, which usually contains functional ATP-binding Walker A motifs, is located within the cytoplasmic loop and is flanked by the second and third TM domains (Ma et al., 2009). Site-directed mutagenesis studies suggest that the function of the Walker A domain varies among different T6SS models. For example, in *Edwardsiella tarda*, the Walker A motif of IcmF is dispensable for T6SS assembly, as the secretion of Hcp and VgrG is not affected by its inactivation (Zheng and Leung, 2007). In contrast, in *Agrobacterium tumefaciens* the Walker A motif of IcmF is crucial for T6SS-mediated Hcp secretion and IcmF also exhibits ATPase activity, which is required to pump Hcp out of the cell. Upon ATP binding and hydrolysis that allows for the recruitment of Hcp to the IcmF/DotU complex, IcmF undergoes a conformational change (Ma et al., 2009, 2012). However, in several examples of IcmF proteins, such as IcmF3 of H3-T6SS in *P. aeruginosa*, Walker A motifs cannot be identified in the cytoplasmic domain.

In *L. pneumophila* T4SS-associated IcmF also plays a role in virulence, where it is involved in intracellular growth and killing of human macrophages (Segal and Shuman, 1999). Insertional mutagenesis of *icmF* in *V. cholerae* has shown that this gene is involved in regulating the motility, conjugation frequency and adherence of this organism to intestinal epithelial cells *in vitro* (Das et al., 2002). Moreover, IcmF limits intracellular growth in macrophages during the late stage of infection and attenuates the lethality of *Salmonella enterica* serovar Typhimurium in a murine host (Parsons and Heffron, 2005). Additionally, IcmF in avian pathogenic *Escherichia coli* (APEC) is involved in motility, biofilm formation, adherence to and invasion of epithelial cells, intra-macrophage survival and infection in chicks (de Pace et al., 2011). Due to the great functional diversity of IcmF proteins, their roles in other bacterial species remain largely unknown.

The major aim of the present study is to characterize the function of IcmF3 in *P. aeruginosa* PAO1. The *icmF3*-deletion mutant and complemented strains were constructed and the effects of *icmF3* mutation on phenotypes *in vitro* and *in vivo* were investigated. We show here that an *icmF3* deletion mutation causes multiple phenotypic changes in *P. aeruginosa*, several of which have not been reported before (such as siderophore production and growth in iron-limited conditions), thus expanding our knowledge of the roles of IcmF proteins.

## Materials and methods

### Bacterial strains, plasmids, and growth media

Bacterial strains and plasmids used in this study are listed in Supplementary Table 1. *Escherichia coli* strains were grown at 37°C in either Luria–Bertani (LB) broth or agar. *P. aeruginosa* PAO1 and its mutants were grown at 37°C in either LB, TSB, casamino acids (CAA) medium (Cornelis et al., 1992) or in succinate minimal medium (Meyer and Abdallah, 1978). Antibiotics were used at the following concentrations for *E. coli*: kanamycin, 50 μg ml^−1^; tetracycline, 15 μg ml^−1^; gentamicin, 15 μg ml^−1^ and for *P. aeruginosa*: kanamycin, 50 μg ml^−1^; chloramphenicol, 30 μg ml^−1^; gentamicin, 100 μg ml^−1^; tetracycline, 200 μg ml^−1^ for plates or 160 μg ml^−1^ for liquid growth.

### In-frame deletion and complementation in *P. aeruginosa*

To generate an *icmF3* deletion mutant, 870 bp upstream and 805 bp downstream of the *icmF3* gene were amplified by overlapping PCR with *Pfu* DNA-polymerase using the primer pairs PAIcmF3 up F/PAIcmF3 up R and PAIcmF3 low F/PAIcmF3 low R (see Supplementary Table 2). The PCR product was inserted into pK18*mobsacB*, a suicide vector, using the *Xba*I and *Hin*dIII sites prior to digestion with *Hin*dIII. The gentamicin resistance cassette from p34s-Gm was subsequently inserted into the same *Hin*dIII site to yield the mutation plasmid pK-F. After mating an *E. coli* S17-1 derivative that carried pK-F with *P. aeruginosa* PAO1 on LB plates at 37°C for 48 h, the cells were suspended in LB and appropriate dilutions were spread on LB plates containing chloramphenicol (to select against the donor strain) plus gentamicin (to select for recipient with non-replicating plasmid integrated into its chromosome). Several colonies were transferred to LB medium and incubated at 37°C overnight before appropriate dilutions were spread on LB plates containing 12% sucrose for counter-selection against single cross-over mutants. Double cross-over mutants resulting in the nonpolar deletion of *icmF3* were verified by PCR using external primer pair PAIcmF3 up F/PAIcmF3 low R and Sanger DNA sequencing. The resulting *icmF3* deletion contains the first 79 codons fused in frame with the last 65 codons. The *pvdA* mutant, *pchD* mutant and *pvdApchD* double mutant were all isolated by the same procedure as the *icmF3* mutant. To complement the Δ*icmF3* strain, PCR-amplified *icmF3* was cloned into the *Eco*RI and *Bgl*II sites of plasmid pME6032, giving rise to the plasmid pME6032-*icmF3*. This plasmid was then transformed into the Δ*icmF3* mutant PAO-F, generating the complemented strain PAO-F (pME6032-*icmF3*).

### Construction of chromosomal fusion reporter strains and β-galactosidase assays

The *phzA1-lacZ, phzA2-lacZ, fliC-lacZ, fliL-lacZ, fliE-lacZ, flgF-lacZ, flgM-lacZ, pchD-lacZ*, and *pchE-lacZ* transcriptional fusions were constructed by PCR amplification of the 1286, 1099, 1120, 1146, 633, 760, 684, 684, and 684 bp upstream DNA region from the *phzA1, phzA2, fliC, fliL, fliE, flgF, flgM, pchD*, and *pchE* gene by using primer pairs PphzA1 F/PphzA1 R, PphzA2 F/PphzA2 R, PfliC F/PfliC R, PfliL F/PfliL R, PfliE F/PfliE R, PflgF F/PflgF R, PflgM F/PflgM R, PpchD F/PpchD R, and PpchE F/PpchE R, respectively (see Supplementary Table 2). PCR amplification products from each of the upstream regions were cloned directly into the pMini-CTX::*lacZ* vector (Becher and Schweizer, 2000; Hoang et al., 2000), yielding a range of *lacZ* reporter constructs as listed in Supplementary Table 1. Promoter fragments were integrated at the CTX phage attachment site in wild-type strain PAO1 and the *icmF3* mutant strain following established protocols (Becher and Schweizer, 2000; Hoang et al., 2000). For β-galactosidase assays, overnight bacterial cultures were diluted 1:500 in TSB or 1:1000 in CAA. Growth and β-galactosidase activity were monitored by harvesting samples at different time points. β-Galactosidase activity was measured according to the Miller method (Miller, 1992) based on *o-*nitrophenyl-β-D-galactopyranoside hydrolysis and expressed in Miller units.

### Motility assays

Swimming motility assays were performed as previously described (Inoue et al., 2008). Briefly, static overnight cultures were stabbed into motility plates [1% tryptone, 0.5% NaCl, 0.3% Bacto™ agar (BD, USA)] and incubated at 37°C. Motility halos were measured after 36 h of incubation.

### Pyoverdine (PVD) measurements

Culture supernatants from cultures grown in CAA were diluted in 100 mM Tris-HCl (pH 8.0), and PVD was measured as OD_405_ normalized by the cell density of bacterial cultures (OD_600_) (Imperi et al., 2009).

### Chrome azurol assay (CAS) for detection of siderophores

Siderophore concentration was estimated according to the method of Schwyn & Neilands (Schwyn and Neilands, 1987). Briefly, the ternary complex of chrome azurol S/iron (III)/hexadecyl trimethylammonium bromide was added to the CAA agar plates as an indicator. A 6 μl sample of a CAA culture (OD_600_ 0.5) was spotted onto CAS plates and incubated overnight. The diameter of the orange halos was measured after the incubation.

### Determination of conjugation frequency

Conjugation frequency was determined following the method of Das et al. (2002), with several modifications. Briefly, overnight cultures of *P. aeruginosa* (recipient) or *E. coli* S17-1 with pMP220 or pBBR1MCS-5 (donor) were washed with LB before mixing at the appropriate ratio in LB. The bacterial mixture was filtered through a 0.45 μm Millipore (Bedford, MA) membrane placed on a reusable filter unit. After filtration, the membrane was placed cell side up on the agar surface of an LB plate. After 36 h incubation, bacteria were resuspended in LB. Undiluted, 1/10 and 1/100 dilutions were plated on LB agar plates containing kanamycin (50 μg ml^−1^) and tetracycline (200 μg ml^−1^) or kanamycin (50 μg ml^−1^) and gentamicin (100 μg ml^−1^) for enumeration of transconjugants. If required, 1 mM IPTG was included in the medium for induction.

### *E. coli* killing assay

The *E. coli* killing assay was modified from previously reported studies (Basler et al., 2013; Jiang et al., 2014). *P. aeruginosa* and *E. coli* DH5α with pBBR1MCS-5 strains were grown overnight and washed with sterile ddH_2_O before mixing at the appropriate ratio in ddH_2_O. The mixture was spotted onto 0.45 μm Millipore (Bedford, MA) membrane overlaid onto a 3% LB-LS agar plate (LB low salt: 10 g peptone and 5 g yeast extract per liter). If needed, 0.5 mM IPTG was included in the medium for induction. The *E. coli* DH5α strain contained plasmid conferred gentamycin gene for selection. The plates were incubated at 37°C for 36 h. Bacterial spots were scraped off and the cells were resuspended in 1 ml LB medium. After serial dilution, *P. aeruginosa* and *E. coli* colonies were counted on selective LB agar plates (kanamycin for *P. aeruginosa*, gentamycin for *E. coli*) and changes of the *P. aeruginosa-E. coli* ratios were determined. At least three biological replicates were analyzed.

### Pyocyanin quantitation assay

Pyocyanin concentration was determined as described by Essar et al. (1990). Bacteria were grown in 5 ml of TSB medium to maximize pyocyanin production. The bacterial cultures were extracted with 3 ml of chloroform and then re-extracted with 1 ml of 0.2 N HCl, leading to the development of a pink to deep red color. The absorbance of this solution was measured at 520 nm. Concentrations, expressed as micrograms of pyocyanin produced per milliliter of culture supernatant, were determined by multiplying the optical density at 520 nm (OD_520_) by 17.072. The concentration values were normalized to the cell density of each sample (OD_600_).

### Biofilm formation assay

Biofilm formation was determined according to the method of O'Toole and Kolter (1998). Briefly, overnight bacterial cultures were diluted 100-fold in fresh LB medium containing 1 mM IPTG and 160 μg ml^−1^ tetracycline. The cell suspension (100 μl) was transferred into each well of a 96-well PVC plate (Sigma) and incubated at 37°C. After incubation for 24 h, the LB medium was removed and the wells were washed twice with phosphate-buffered saline (PBS). The cells that adhered to the wells were stained with 0.1% crystal violet for 10 min and then washed twice with PBS. The cell-bound dye was eluted in 4 ml of 95% ethanol, and the absorbance of the eluted solution was measured using a microplate reader at 570 nm. The average and SD values were calculated from eight wells for each sample.

### Determination of MICs and MBCs

Minimum inhibitory concentrations (MICs) and minimal bactericidal concentrations (MBCs) were determined according to the method of Pompilio et al. (2012) with some modifications. Briefly, a twofold dilution series of LB broth containing different antimicrobials was prepared in glass tubes. Subsequently, 5 ml of LB containing the same antimicrobial was inoculated with 5 μl of a standardized inoculum, corresponding to a final test concentration of about 0.8–1 × 10^5^ CFU ml^−1^. After incubation at 37°C with shaking at 180 rpm for 18 h, the MIC was noted as the lowest concentration of the tested agent that completely inhibited visible growth. To measure the MBC, 100 μl of broth from clear tubes was plated on LB agar plates, and incubated at 37°C for 24 h. MBC was defined as the lowest concentration of the tested agent killing at least 99.99% of the original inoculum.

### *Caenorhabditis elegans* killing assay

Bacteria were grown overnight at 37°C and supplemented with nematode growth medium (NGM) according to the published method (Tan et al., 1999). NGM plates were incubated firstly at 37°C for 24 h and then at 25°C for 24 h before seeding with adult worms (N2 Bristol). Before solidification, all experimental plates were supplemented with 200 μM 5-fluorodeoxyuridine, which was used to prevent the development of progeny. In each assay, 40–50 adult nematodes were transferred to a single plate. Plates were incubated at 25°C and scored for live worms each day. The experiments were conducted in triplicate and *E. coli* OP50 was used as a negative control. A worm was considered dead when it no longer responded to touch. Any worms that died as a result of getting stuck to the wall of the plate were excluded from the analyses.

### IcmF sequence alignment and phylogenetic tree construction

The entire IcmF protein sequences from various bacterial species deposited at the NCBI database were used for sequence alignment and tree construction. Sequences were aligned using ClustalW (Thompson et al., 1994). A distance tree was calculated from the alignment (with gapsexcluded) by the Neighbor Joining method (Saitou and Nei, 1987), as implemented in MEGA6 (Tamura et al., 2013), using a Poisson correction model. Bootstrap values (% from 1000 replicates) over 50% are indicated at the nodes. All positions containing gaps and missing data were eliminated from the dataset (complete deletion option).

### Statistical analysis

All experiments were performed at least in triplicate and repeated on two different occasions. Data are expressed as mean ± SD. Differences between frequencies were assessed by the Student's *t*-test (bilateral and unpaired). Statistical analysis of results was conducted with GraphPad Prism version 5.00 (GraphPad software Inc.; San Diego, CA, USA), using a *p*-value of < 0.05 as statistically significant.

## Results

### Bioinformatic characterization of *icmF3*

**Figure 1:**
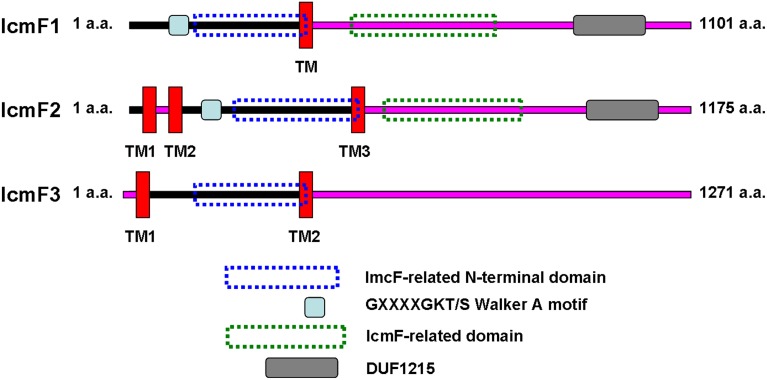
**Comparison of the predicted domain organization of IcmF1, IcmF2, and IcmF3 of ***P. aeruginosa*** PAO1**. IcmF protein domain structures were analyzed using the software tools available at the ExPASY website (http://us.expasy.org/tools/). Transmembrane domains were analyzed with TMHMM (Krogh et al., 2001) prediction programs. InterProScan (Zdobnov and Apweiler, 2001) was used for the analysis of IcmF domain architecture. IcmF amino acids predicted to face the cytoplasmic side are indicated with a black line, and those facing the periplasm are indicated with a pink line.

**Figure 2:**
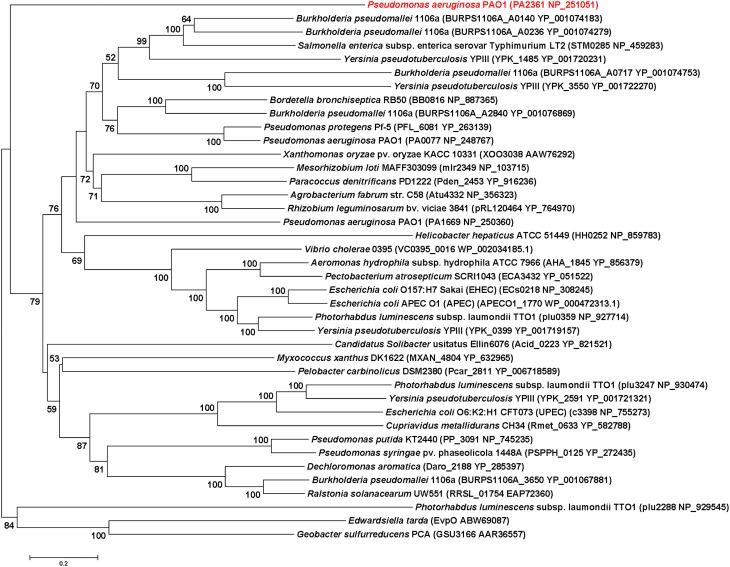
**Evolutionary relationships of 40 IcmF orthologues of various bacterial species**. Tree construction and other details are given in the Materials and Methods. The scale bar indicates 0.2 substitutions per site.

IcmF is conserved in the T6SS clusters of all studied bacterial pathogens. Three *icmF* homologs PA0077 (*icmF1*), PA1669 (*icmF2*), and PA2361 (*icmF3*) are present in *P. aeruginosa* strain PAO1. We performed domain architecture analysis to reveal different features between IcmF3 and the other two IcmF homologs. The Walker A motifs, IcmF-related domains and DUF1215 domains, which are present in both IcmF1 and IcmF2, were not detected in IcmF3 (Figure 1). To further assess the phylogenetic relationship between *icmF3* and other bacterial *icmF* genes, a multiple-sequence alignment was conducted using ClustalW followed by construction of a neighbor joining tree (Figure 2, Supplementary Figure 1). The IcmF3 from strain PAO1 formed an independent branch on the phylogenetic tree and a long branch length was observed between IcmF3 and other IcmF proteins. These results suggest that IcmF3 from strain PAO1 may represent a distinct branch with novel functions that have not been previously characterized.

### Colony morphology and growth characteristics

**Figure 3:**
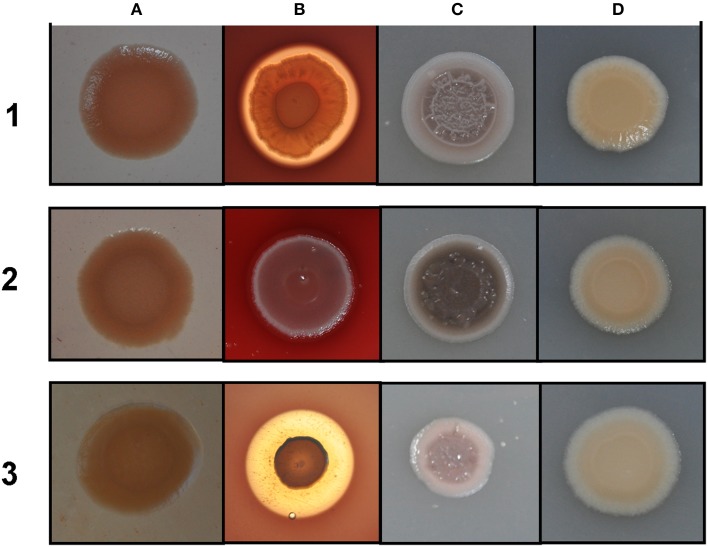
**Different colony morphologies of ***P. aeruginosa*** on LB agar with Congo red (A), blood agar (B), LB agar supplemented with 4 mM FeSO_4_ (C), LB agar (D)**. 1 mM IPTG was included in the media for induction. (1) wild type PAO1 with pME6032; (2) *icmF3* deletion mutant PAO-F with pME6032; (3) PAO-F with pME6032-*icmF3*.

**Figure 4:**
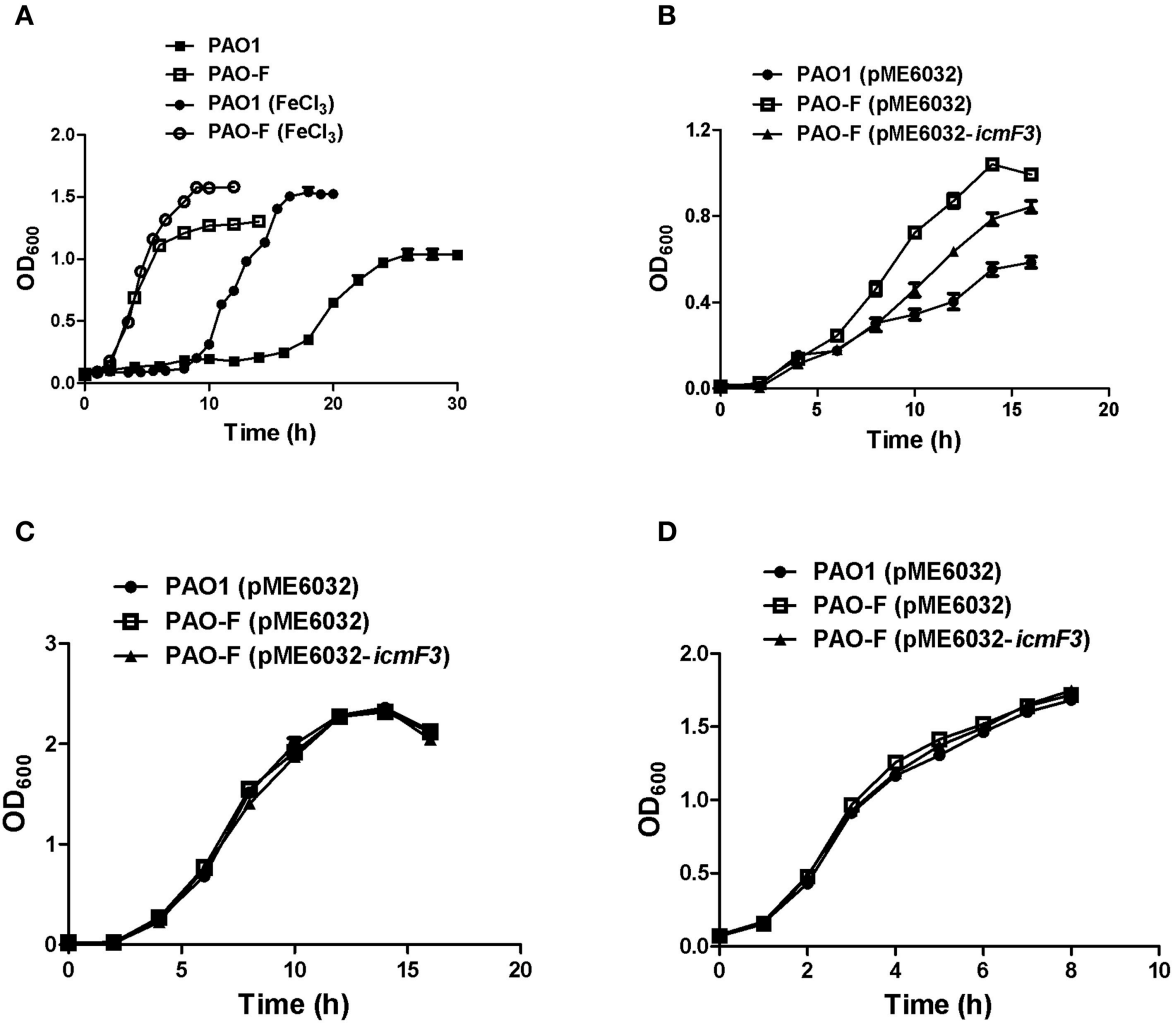
**Growth curves of ***P. aeruginosa*** in LB and iron-limiting media at 37°C**. **(A)** *P. aeruginosa* PAO1 and PAO-F were cultured in CAA alone or CAA supplemented with 200 μM FeCl_3_ and growth was measured as a function of time. **(B–D)** Growth of *P. aeruginosa* PAO1 with pME6032, PAO-F with pME6032, PAO-F with pME6032-*icmF3* in succinate minimal medium **(B)**, succinate minimal medium supplemented with 50 μM FeCl_3_ **(C)**, and LB **(D)**. If needed, 1 mM IPTG was included in the medium for induction. These data are representative of a minimum of three independent experiments. Error bars represent the standard deviations.

Rakhimova et al. (2008) reported that mini-Tn5 transposon insertion mutation in the *icmF3* gene of *P. aeruginosa* strain TBCF10839 caused a pleiotropic B phenotype (formation of white colonies on blood agar or iron supplemented agar medium, with the formation of colonies with red centers on Congo red agar). However, the colony morphology variant seen in the TBCF10839 *icmF3* transposon mutant could not be reproduced by a transposon insertion in *icmF3* of the PAO1 strain (Rakhimova et al., 2008). Similarly, the *icmF3* deletion mutant of *P. aeruginosa* strain PAO1 in this study did not display a pleiotropic B phenotype, but novel differences between the wild-type strain and the *icmF3* deletion mutant were observed. However, as the detailed results of colony morphology variation caused by a transposon insertion in PAO1 *icmF3* were not shown in Rkhimova et al. study, comparison between the morphology variation in these two *icmF3* mutants could not be performed. Compared to the wild-type strain, the *icmF3* deletion mutant exhibited impaired hemolytic activity on blood agar (Figure 3B). Moreover, the colonies formed by the wild-type strain were light brown colored on iron supplemented agar, however, the *icmF3* deletion mutant colonies were dark brown, indicating accumulation of more iron in the cells (Figure 3C). Complementation of the *icmF3* deletion mutant with full-length *icmF3* under the control of the pTac promoter restored the wild-type phenotype (Figure 3). Additionally, it was found that growth of *P. aeruginosa* PAO1 on iron-limiting CAA media at 37°C was drastically stimulated by the inactivation of *icmF3* (the doubling time was reduced from ~188 to ~73 min; Figure 4A, Table 1). The addition of FeCl_3_ to the CAA medium accelerated the growth of the wild-type (the doubling time was reduced from ~188 to ~105 min; Figure 4A, Table 1), however, this exerted only a marginal effect on the growth of the *icmF3* mutant (the doubling time was reduced from ~73 to ~70 min; Figure 4A, Table 1). We tried to complement the *icmF3* mutant with a plasmid containing complete *icmF3* gene, but we could only observe partial complementation (data not shown). The reason for the partial complementation could be due to gene dosage-dependent functions. Similarly, the growth rate of *P. aeruginosa* PAO1 at 37°C was also noticeably stimulated by inactivation of *icmF3* when grown on iron-limiting succinate minimal medium (Figure 4B). However, no significant difference was observed in the growth rate between the *icmF3* mutant and the wild-type strain PAO1 when grown in LB medium or succinate minimal medium supplemented with FeCl_3_ at 37°C (Figures 4C,D, Table 1).

**Table 1:** **Doubling time for the ***P. aeruginosa*** PAO1 and PAO-F^δ^**.

**Strains**	**Growth media**		
	**LB**	**CAA**	**CAA + 200 μM FeCl_3_**
PAO1	43.7 ± 1.0	187.8 ± 8.0	105.2 ± 6.1
PAO-F	46.6 ± 1.2	73.3 ± 0.7	70.1 ± 0.2

δ *Bacterial growth in liquid culture was assessed by optical density at 600 nm (OD_600_), and doubling times (in minute) during exponential phase growth were calculated based on time required for 2-fold increases in the OD_600_*.

### Production of siderophores

**Figure 5:**
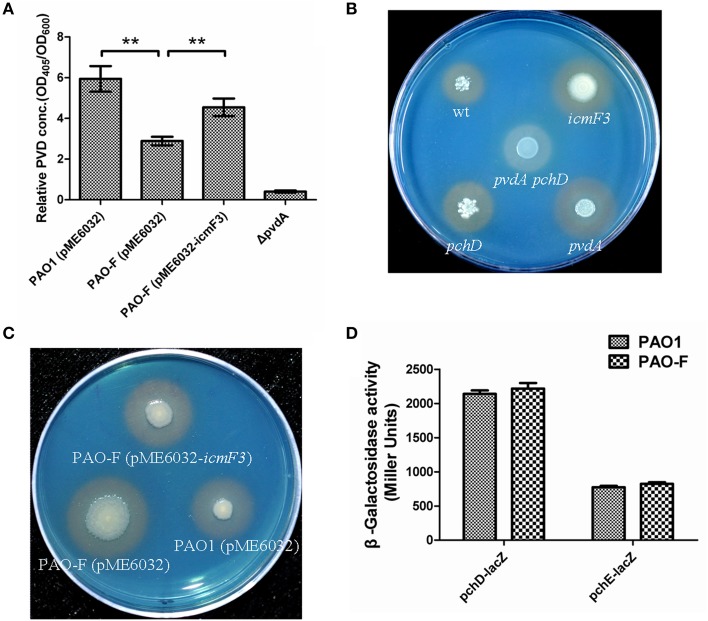
**Deletion of ***icmF3*** affects siderophore production**. Wild-type and mutant strains were grown at 37°C in CAA to stationary phase. **(A)** Relative levels of extracellular pyoverdine (PVD) in culture supernatants were determined spectrophotometrically. Values are presented as the mean (SD) of five independent assays. The Δ*pvdA* strain was used as a negative control. ^**^P < 0.01. **(B)** Comparison of the colored halos around wild-type (top left), *pchD* pyochelin-negative mutant (bottom left), a pyoverdine-negative *pvdA* mutant (bottom right), a double *pvdApchD* mutant (center), and the *icmF3* mutant (top right) colonies. **(C)** Comparison of the colored halos around PAO1 (pME6032) (bottom right), PAO-F (pME6032) (bottom left), PAO-F (pME6032-*icmF3*) (top) colonies. **(D)** Cells were cultured in CAA and levels of *pchDCBA* and *pchEFGHI* transcription in *P. aeruginosa* PAO1 and *icmF3* mutant cells were monitored using the *pchD–lacZ* and *pchE–lacZ* transcriptional fusions, respectively. If needed, 1 mM IPTG was included in the medium for induction. The graphs show the mean and SD of two experiments performed in eight replicates each time.

As growth of *P. aeruginosa* PAO1 in iron-limiting media at 37°C was drastically stimulated by inactivation of *icmF3*, this lead us to hypothesize that iron transport would be enhanced by mutating *icmF3*. Thus, we investigated the impact of the deletion of *icmF3* on the production of siderophores, which are crucial for iron transport in strain PAO1. Such an alteration in growth might be expected to have a positive effect on the production of siderophores. Two siderophores are produced by *P. aeruginosa* PAO1, pyoverdine, and pyochelin, characterized by high and low affinities for iron, respectively (Cornelis and Dingemans, 2013). Thus, we measured pyoverdine production first. Unexpectedly, under iron-limiting conditions, pyoverdine production was significantly reduced (approximately 50% of the wild-type level) by mutation of the *icmF3* gene, which was partially rescued by the introduction of the complementation plasmid (Figure 5A). However, high levels of siderophore production by the *icmF3* mutant on the CAS plates were evident (Figures 5B,C, Supplementary Figure 2), and the halo produced around the inoculated bacteria was similar in size and color to that produced by a pyoverdine negative mutant (Δ*pvdA*) that still produces another siderophore, pyochelin. To assess whether the siderophore activity seen in the *icmF3* mutant on CAS agar plates is due to pyochelin, we investigated expression of the pyochelin biosynthetic operon in this mutant. Unexpectedly, the β-galactosidase assay revealed that *pchDCBA* and *pchEFGHI* transcript levels did not change in *icmF3* mutant, compared to wild-type PAO1 (Figure 5D). Since the *icmF3* mutation had been shown to enhance growth in iron-limiting media, we proposed that alterations in siderophore production seen on the CAS plates were due to altered growth.

### Involvement of *icmF3* in plasmid conjugation

**Figure 6:**
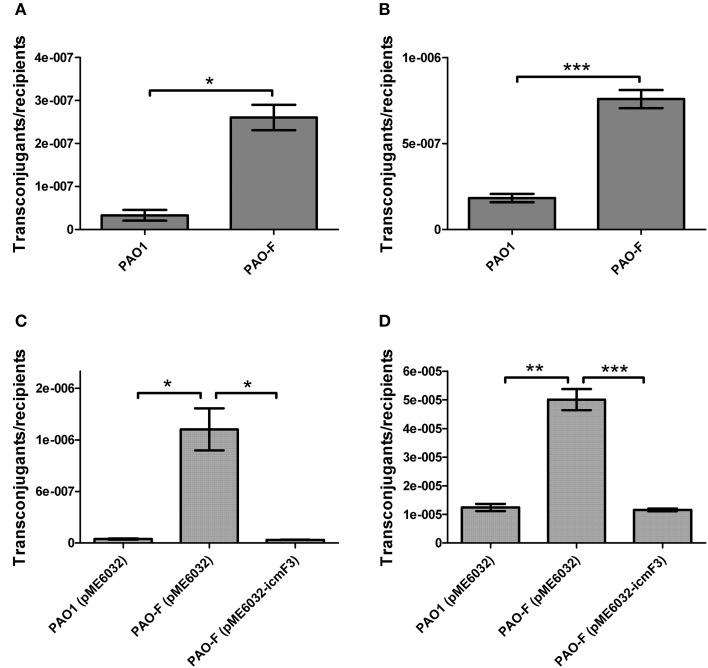
**Conjugation frequencies of wild type PAO1 and ***icmF3*** mutant as recipients**. **(A,B)** Recipient strains: wild type PAO1 and *icmF3* mutant PAO-F; donor strain: *E. coli* S17-1 with pMP220. Donor and recipient strains were mixed in 1:1 ratio for **(A)** and in 10:1 ratio for **(B)**. **(C,D)** Recipient strains: PAO1 (pME6032), PAO-F (pME6032), PAO-F (pME6032-*icmF3*); donor strain: *E. coli* S17-1 with pBBR1MCS-5. Donor and recipient strains were mixed in 1:1 ratio for **(C)** and in 10:1 ratio for **(D)**. If needed, 1 mM IPTG was included in the medium for induction. Data are presented as mean ± SD of a minimum of three independent experiments. ^*^P < 0.05, ^**^P < 0.01, ^***^P < 0.001.

**Figure 7:**
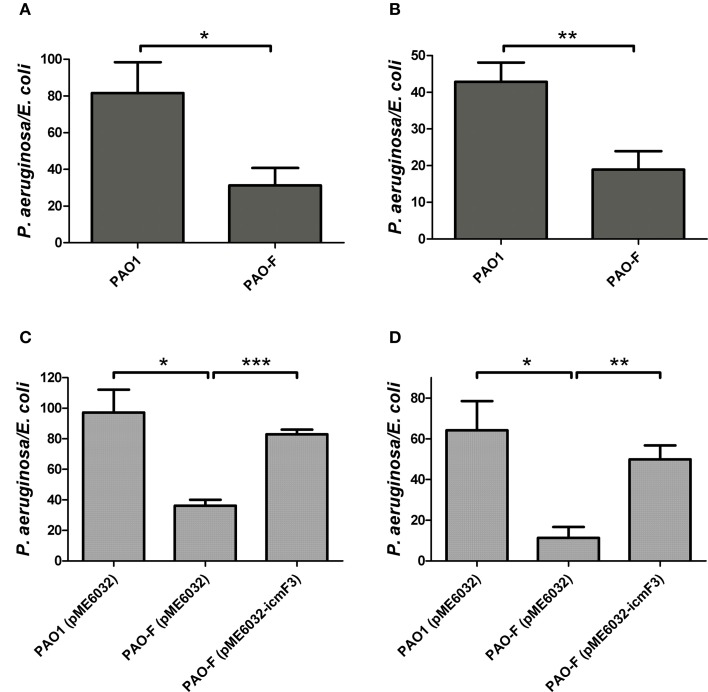
**Competitive growth outcome of the indicated ***P. aeruginosa*** strains (x axis) against ***E. coli*** DH5α with pBBR1MCS-5 strain at 37°C for 36 h**. *P. aeruginosa* and *E. coli* strains were mixed in 1:2 ratio for **(A,C)** and in 10:1 ratio for **(B,D)**. If needed, 1 mM IPTG was included in the medium for induction. The competitive index result is calculated as the final c.f.u. ratio (*P. aeruginosa*/*E. coli*) divided by the initial ratio. Error bars represent ± SD (*n* = 3). ^*^P < 0.05, ^**^P < 0.01, ^***^P < 0.001.

In a previous study, it has been reported that a strain lacking *icmF* functions slightly better as a conjugal recipient of plasmid pRK290 when compared to the wild-type *V. cholerae* strain (Das et al., 2002), and it is the same case for the *L. pneumophila icmF* mutant (Segal and Shuman, 1998). The ability in conjugation-mediated DNA acquisition of the wild-type and *icmF3* mutant was tested via a mating experiment, using *icmF3* deletion mutant PAO-F or wild-type strain PAO1 as the recipients and *E. coli* S17-1 containing plasmid pMP220 or pBBR1MCS-5 as the donor. Similar as the *V. cholerae icmF* mutant, the mutation in *icmF3* gene in strain PAO1 resulted in an increased frequency of conjugation compared to wild-type (Figures 6A,B). Complementation with an *icmF3*-expressing plasmid restored the frequency of conjugation to parental levels (Figures 6C,D). Moreover, previous studies demonstrated that the H1-T6SS of *P. aeruginosa* PAO1 protects cells from RP4-conjugation mediated foreign DNA transfer (Ho et al., 2013; Leroux et al., 2015). Jiang et al. (2014) reported that the H3-T6SS contributes to interbacterial competitive fitness of *P. aeruginosa* via delivery of the toxin PldB to host cells (Jiang et al., 2014). To assess whether H3-T6SS in *P. aeruginosa* had similar properties, we performed interbacterial growth competition experiments between *P. aeruginosa* and *E. coli*. Previous quantitative RT-PCR analysis has indicated that H3-T6SS loci are upregulated under the prolonged static culture at 37°C (Jiang et al., 2014). The bacterial killing assay using *E. coli* as target cells was performed under these conditions. *E. coli* killing assay showed that the ablation of IcmF3 function abolished bacterial toxicity, reducing the normal growth advantage (Figures 7A,B, Supplementary Table 3). Note that the growth advantage could be restored to the *icmF3* knockout strain by transcomplementation with a functional *icmF3* gene (Figures 7C,D, Supplementary Table 3). These results demonstrate that the increased DNA acquisition in *icmF3* mutant may be caused by the reduced *E. coli* killing ability.

### Production of pyocyanin

**Figure 8:**
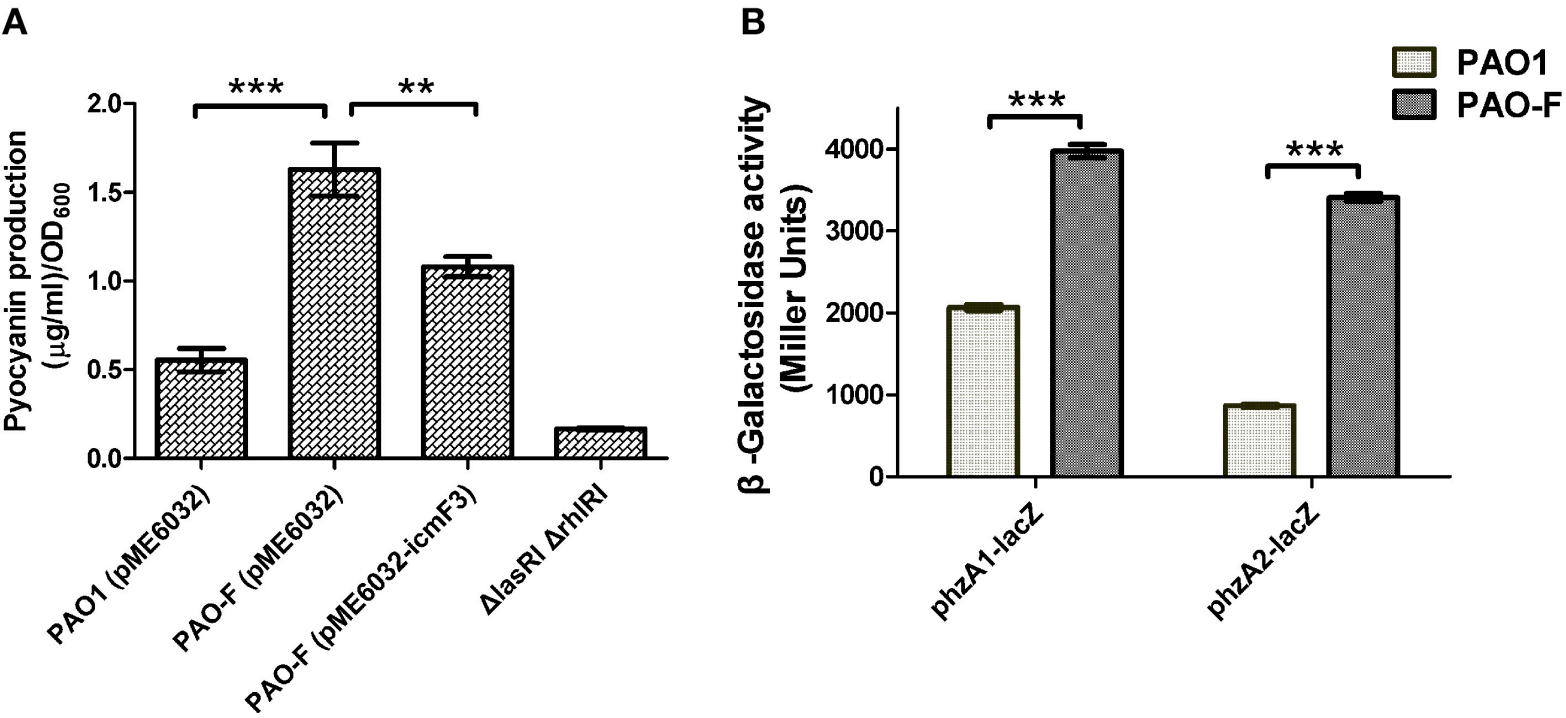
**Pyocyanin production by ***P. aeruginosa*** WT and ***icmF3*** mutant**. Cells were grown in TSB broth to an OD_600_ of 4.0 at 37°C with aeration. **(A)** Spectrophotometric quantitation of pyocyanin production in *P. aeruginosa* cultures. Extraction procedures are outlined in Materials and methods. The Δ*lasRI*Δ*rhlRI* strain was used as a negative control. **(B)** Levels of *phzA1B1C1D1E1F1G1* and *phzA2B2C2D2E2F2G2* transcription in *P. aeruginosa* WT and the *icmF3* mutant were monitored using the *phzA1–lacZ* and *phzA2–lacZ* transcriptional fusions, respectively. If needed, 1 mM IPTG was included in the medium for induction. The graphs show the mean and SD of two experiments performed in eight replicates each time. ^**^P < 0.01, ^***^P < 0.001.

During the course of our experiments we identified a correlation between the *icmF3* mutation and increased production of the blue-green phenazine pigment pyocyanin. Quantification of pyocyanin from cells grown in TSB media showed that the production of this pigment was significantly increased in the *icmF3* mutant compared to wild-type (approximately threefold greater in the *icmF3* mutant; Figure 8A). The wild-type phenotype was partially restored when the *icmF3* mutant was complemented by the introduction of the plasmid pME6032-*icmF3* (Figure 8A). To assess the reasons underlying the overproduction of pyocyanin in the *icmF3* mutant, we investigated expression of two phenazine biosynthetic loci in this mutant. β-galactosidase assays revealed that the transcription levels of *phzA1* operon (*phzA1B1C1D1E1F1G1*) and the *phzA2* operon (*phzA2B2C2D2E2F2G2*) increased at least 2-fold in the *icmF3* mutant, when compared to the wild-type strain (Figure 8B). These data suggest that the *icmF3* mutation increases expression of the phenazine biosynthetic operon, consequently leading to the overproduction of pyocyanin.

### Swimming motility

**Figure 9:**
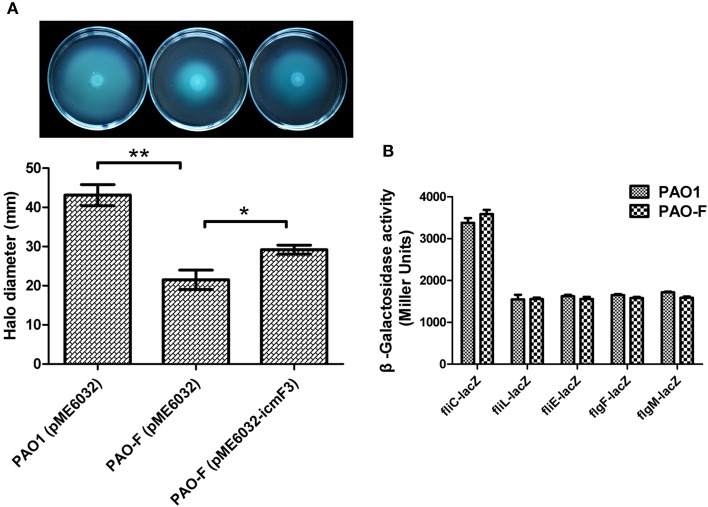
**IcmF3 is involved in motility**. **(A)** Motility assay on motility agar plates containing 1 mM IPTG and 100 μg ml^−1^ tetracycline after 36 h. Halos around the colonies were measured after 36 h of incubation at 37°C. The graph shows the mean and SD of two experiments performed in five replicates each time. ^*^P < 0.05, ^**^P < 0.01. **(B)** The expression of flagellar biogenesis genes in *P. aeruginosa* WT and the *icmF3* mutant. Levels of *fliCfleL, fliLMNOPQRflhB, fliEFGHIJ, flgFGHIJKL*, and *flgMN* transcription in *P. aeruginosa* WT and the *icmF3* mutant were monitored using the *fliC-lacZ, fliL-lacZ, fliE-lacZ, flgF-lacZ*, and *flgM-lacZ* transcriptional fusions, respectively. The graphs show the mean and SD of two experiments performed in eight replicates each time.

Given that in *V. cholerae* O395 and avian pathogenic *E. coli* SEPT362, IcmF has also been implicated in motility (Das et al., 2002; de Pace et al., 2011), we investigated whether *icmF3* in *P. aeruginosa* had similar properties. Swimming motility assays showed that the *icmF3* mutant was much less motile compared to the wild-type strain, and that motility was partially restored upon complementation (Figure 9A). In *E. coli*, an *icmF* mutant was found to be non-motile due to decreased expression of the flagella regulon (de Pace et al., 2011). Therefore, we also investigated the expression of several flagellar biogenesis genes in the *icmF3* mutant. Unexpectedly, β-galactosidase assays revealed that transcription levels of the major genes involved in swimming motility (*fliCfleL, fliLMNOPQRflhB, fliEFGHIJ, flgFGHIJKL*, and *flgMN*) were at comparable levels in both the *icmF3* mutant and wild-type PAO1 (Figure 9B). Since swimming motility is affected by multiple factors, further studies are needed to determine the underlying mechanisms for the inhibition of swimming motility due to *icmF3* mutation.

### Biofilm formation is influenced by *icmF3*

**Figure 10:**
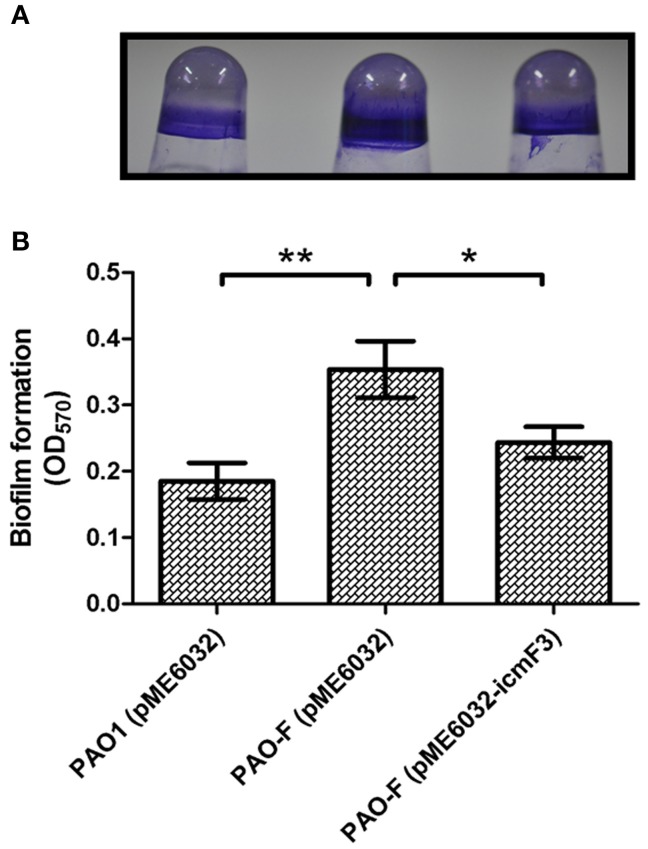
**Deletion of ***icmF3*** affects biofilm formation**. Cells were grown for 24 h in PVC 96-well plates containing LB, 1 mM IPTG and 160 μg ml^−1^ tetracycline. Biofilm formation the strains was determined by crystal violet staining **(A)** and quantified using optical density measurement **(B)**. ^*^P < 0.05, ^**^P < 0.01.

In avian pathogenic *E. coli* SEPT362, an *icmF* mutant was defective for biofilm formation on abiotic surfaces (de Pace et al., 2011). Here we also tested the influence of the *P. aeruginosa* i*cmF3* mutation on biofilm formation on abiotic surfaces. Using the crystal violet biofilm assay (O'Toole and Kolter, 1998), we demonstrated that the *icmF3* mutant strain had enhanced biofilm formation on 96-well PVC plates at 37°C, compared to the wild-type strain (Figure 10). As the *icmF3* mutation did not show a growth defect at 37°C, this suggests that the influence on biofilm formation is not due to an altered growth phenotype (Figure 4D). Complementation with *icmF3* reduced biofilm formation to wild-type levels (Figure 10). These results suggest that IcmF3 may be involved in a signaling pathway that modulates biofilm formation.

### IcmF3 is important for aminoglycoside resistance

Biofilm formation has been frequently linked to bacterial resistance to antibiotics. As a hyperbiofilm forming phenotype was observed in the *icmF3* mutant, we wondered whether this gene is involved in decreased antibiotic resistance. We determined the MIC and MBC for planktonic cells when grown in the presence of tobramycin, gentamicin, tetracycline, or ciprofloxacin (Table 2). No significant differences in the MIC or MBC to tetracycline and ciprofloxacin (Table 2) were detected between the *icmF3* mutant and the wild-type strain PAO1. However, deletion of *icmF3* resulted in a 2- to 4-fold increase in resistance to tobramycin and gentamicin. Collectively, these results showed that *icmF3* is involved in decreased aminoglycoside resistance in *P. aeruginosa*.

**Table 2:** **MICs and MBCs for the ***P. aeruginosa*** PAO1 and PAO-F^α^**.

**Strain**	**Tobramycin**		**Gentamicin**		**Tetracycline**		**Ciprofloxacin**	
	**MIC**	**MBC**	**MIC**	**MBC**	**MIC**	**MBC**	**MIC**	**MBC**
PAO1	4	32	8-16	256	32	512	0.5	64
PAO-F	16	128	32	512	32	512	0.5	64

α *MICs and MBCs (in micrograms per milliliter) represent the modes of at least six replicates*.

### IcmF3 is involved in virulence toward C. elegans

**Figure 11:**
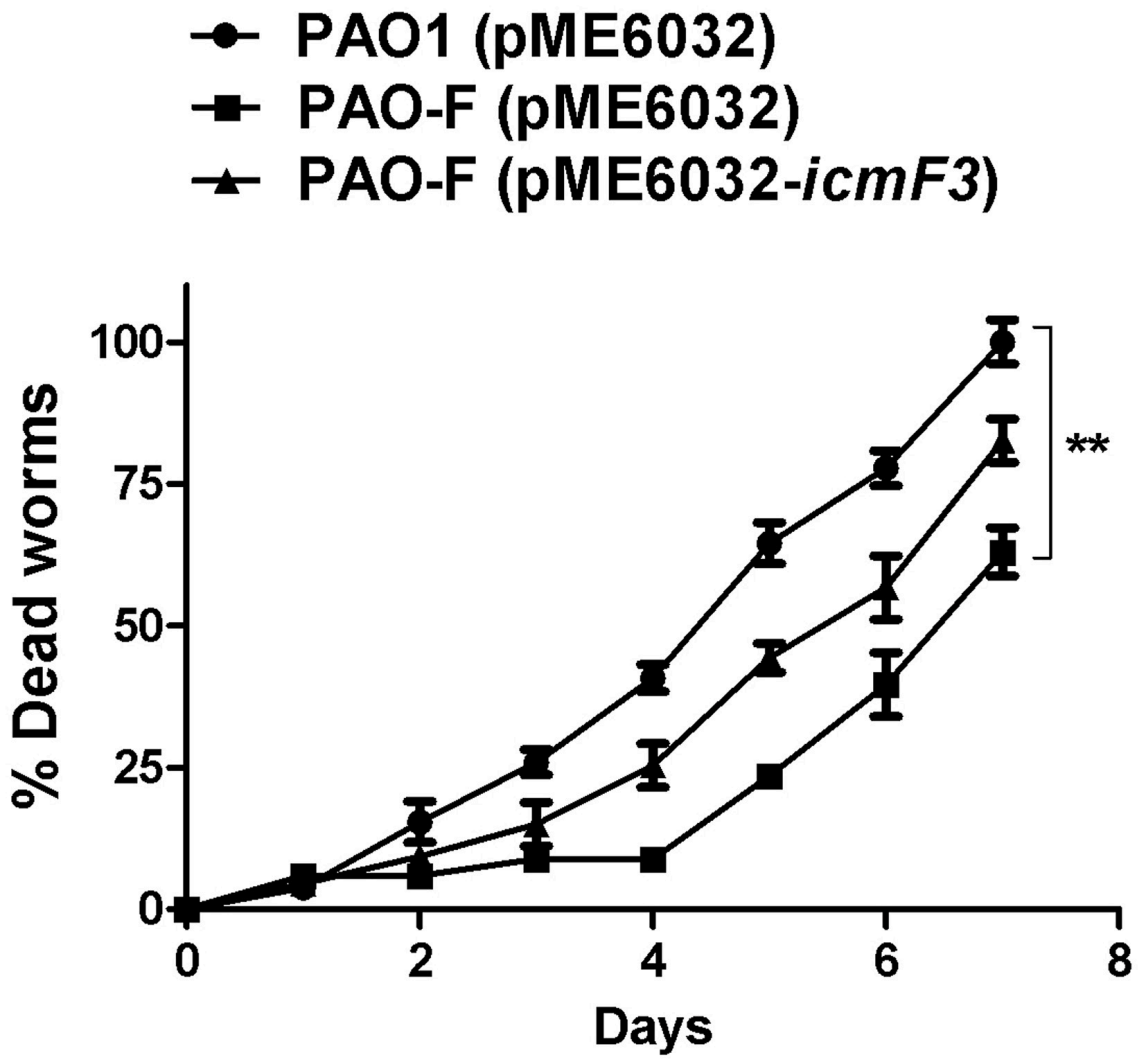
**IcmF3 is required for virulence in ***C. elegans*****. *C. elegans* was infected with PAO1 and the *icmF3* mutant. 1 mM IPTG was included in the medium for induction. The kinetics of the killing of *C. elegans* by *P. aeruginosa* is shown for each strain. ^**^P < 0.01.

The results outlined above revealed a strong correlation between IcmF3 and virulence related characteristics, such as motility and the biofilm formation. IcmF has been shown to be involved in avian pathogenic *E. coli* virulence in chicks (de Pace et al., 2011), and a PAO1 H3-T6SS mutant, which also harbors a deletion of the *clpV3* gene, showed reduced virulence in a worm model (Sana et al., 2013). Based on these phenotypes, we tested whether *icmF3* is involved in the pathogenesis of PAO1 using a *C. elegans* infection model. Worms were infected with wild-type PAO1, the *icmF3* mutant and the complemented strain, which showed that the *icmF3* mutant was significantly less virulent than PAO1. Worms infected with the mutant strain appeared to die on average 2 days after infection, which was significantly longer that worms infected with the wild-type strain. Complementation of *icmF3 in trans* partially rescued this phenotype, however, the complemented strain was not as virulent as the wild-type (Figure 11). As complementation was performed with the *icmF3* gene cloned into a plasmid, the different infection kinetics may be a result of gene dosage effects. Together, these data demonstrated that IcmF3 is required for virulence in *P. aeruginosa* PAO1.

## Discussion

IcmF is localized in the inner membrane and involved in the process of effector delivery into target cells and usually contains several transmembrane domains and a putative Walker A nucleotide-binding motif (Ma et al., 2009). In the present study, we characterized the function of IcmF3 from *P. aeruginosa* PAO1, which lacks a Walker A motif, IcmF-related domain and DUF1215 domain, differentiating it from the other IcmF homologs in *P. aeruginosa* PAO1. The peculiarity of IcmF3 is further supported by our phylogenetic analysis. Our results suggest that IcmF3 plays a role as a novel modulator of multiple virulence-related characteristics in *P. aeruginosa* PAO1, including DNA acquisition, motility, biofilm formation, and virulence, which are all previously reported functions of IcmF in other bacterial species (Das et al., 2002; Parsons and Heffron, 2005; de Pace et al., 2011). Furthermore, we have also identified novel effects related to IcmF3, including growth in iron-limited conditions, siderophore production, pyocyanin production, and aminoglycoside resistance.

### Motility, biofilm formation, and aminoglycoside resistance

Mutation of *icmF3* resulted in impaired motility, enhanced biofilm formation and increased aminoglycoside resistance. Mutation of *icmF* in an avian pathogenic *E. coli* strain resulted in decreased biofilm formation and loss of motility due to decreased expression of the flagella regulon (de Pace et al., 2011). In the present study we have also shown that mutation of *icmF3* results in impaired motility of *P. aeruginosa* PAO1, although the expression of these flagella genes was unchanged in the *icmF3* mutant, indicating that IcmF3 does not exert its effect on motility through control of the flagella genes. The role of IcmF in biofilm formation in *P. aeruginosa* and *E. coli* appears to be different, indicating functional differences between IcmF orthologues in different bacterial species. The impaired motility and increased biofilm formation observed in the present study are consistent with each other, as several previous studies have demonstrated that biofilm formation requires the inhibition of cell motility (Guttenplan and Kearns, 2013). However, it has also been indicated that motility is necessary for biofilm formation (Flagellar and twitching motility are necessary for *P. aeruginosa* biofilm development.). One explanation of these conflicting phenomenon would be that motility is required in the initial stage of biofilm development, while over a long term the motility ability is diluted to extinction during late of biofilm development; thus, the impaired motility and enhanced biofilm by mutation of *icmF3* could be due to its role in balancing the transition of the two stages of biofilm development. Additionally, aminoglycoside antibiotics have been shown to induce biofilm formation in *P. aeruginosa* and *E. coli* (Hoffman et al., 2005). Recent screening studies employing the comprehensive non-redundant Harvard PA14 *P. aeruginosa* mutant library have shown that many mutations can lead to increased aminoglycoside resistance. The *P. aeruginosa* aminoglycoside resistome involves 150 different genes from a range of functional categories (Schurek et al., 2008; Dötsch et al., 2009). However, here we performed the MIC and MBC test on the planktonic cells, which suggested the *icmF3* mutant is more resistant than the wild-type strain. Thus, the enhanced aminoglycoside resistance by mutation of *icmF3* could not be in a biofilm-dependent manner, although biofilm formation was also enhanced by the mutation of *icmF3*.

### DNA acquisition

Recently, LeRoux et al. showed that the T4SS encoded on the RP4 plasmid induces lysis within a subset of *P. aeruginosa* cells, which in turn induces *P. aeruginosa* response to antagonism (PARA), leading to T6SS-dependent *E. coli* cell death. This process could be an altruistic behavior mechanism of *P. aeruginosa* that both aborts the T4SS-dependent transfer event and alerts surrounding cells, thus decreasing the probability of foreign DNA acquisition by the colony (Leroux et al., 2015). Our results demonstrated that the deletion of *icmF3* gene in *P. aeruginosa* PAO1 reduced *E. coli* killing ability (Figure 7). Thus, the increased DNA acquisition phenotype shown by the *icmF3* mutant can be explained as follows: H3-T6SS-dependent *E. coli* cell death is abolished by *icmF3* mutation and the signal to abort the T4SS-dependent DNA transfer is quenched. A recent study has nicely shown that the T6SS of *V. cholerae* is part of the competence regulon (Borgeaud et al., 2015). When the T6SS regulon is co-induced with competence genes, by killing non-immune bacteria, the DNA that is released may be accessible for horizontal gene transfer. Since IcmF3 seems to be required for an opposite function (the mutant presents an increased frequency of conjugation and might also be affected for H3-T6SS antibacterial function), the role of T6SS is more versatile than previously thought.

### Growth in iron-limiting medium and production of siderophores

Our results revealed a potential interaction between IcmF3 and iron acquisition, which has not been previously reported. The growth of *P. aeruginosa* PAO1 in iron-limiting media was dramatically enhanced by inactivation of *icmF3*. These data suggested that *icmF3* deletion may affect the production of siderophores. Unexpectedly, the *icmF3* mutant does not produce pyoverdine, while pyochelin production was not significantly affected by *icmF3* inactivation. These results indicate that the improved growth phenotype of the *icmF3* mutant in iron-limiting media may be not linked to the production of siderophores. One possible explanation for the observed phenotype is that more iron is stored in *icmF3* mutant cells. Intracellular iron accumulation provides a source of this essential mineral when external supplies become limited. In support of our hypothesis we indeed found that more iron was accumulated in *icmF3* mutant cells when grown in iron supplemented media (Figure 3C).

### Phenazine biosynthesis

The observation of increased compound biosynthesis in the *icmF3* mutant was unexpected. In this study, we demonstrated that two phenazine biosynthetic loci were up-regulated in the *icmF3* mutant, which led to increased levels of pyocyanin. Two homologous operons are involved in the synthesis of pyocyanin in *P. aeruginosa, phzA1* (*phzA1B1C1D1G1*) and *phzA2* (*phzA2B2C2D2G2*) (Mavrodi et al., 2001). Pyocyanin production is tightly regulated by quorum-sensing systems in *P. aeruginosa*. Both the PQS and *rhl* systems positively regulate *phzA1* expression (Latifi et al., 1995; Diggle et al., 2003), while the LuxR-type quorum sensing regulator QscR negatively regulates the expression of both *phzA1* and *phzA2* (Ledgham et al., 2003). These results suggest that enhanced pyocyanin production occurs either directly by IcmF3-dependent transcriptional activation of *phzA1*/*phzA2* or indirectly via decreased QscR expression, which negatively influences pyocyanin production.

In conclusion, the present study demonstrated the involvement of IcmF3 in a broad range of *P. aeruginosa* phenotypes, suggesting that the role of IcmF is more versatile than previously thought. This is consistent with our recent findings that T6SS not only contributes to bacterial pathogenesis, but also defenses against diverse environmental insults through unexpected mechanisms (Zhang et al., 2013; Wang et al., 2015). However, the detailed mechanisms underlying the regulatory effects warrant further investigation.

### Conflict of interest statement

The authors declare that the research was conducted in the absence of any commercial or financial relationships that could be construed as a potential conflict of interest.

## Supplementary material

Supplementary Table 1 **Strains and plamids used in this work**.

Supplementary Table 2 **Sequences of the PCR primers used in this work**.

Supplementary Table 3 **Original data for the ***E. coli*** killing assay**.

Supplementary Figure 1 **Multiple sequence alignment of the full-length IcmF protein sequences from various bacterial species**. Sequences are labeled to the left by protein name and accession number, with residue numbers corresponding to the last alignable residue indicated to the right of the sequence. Red outline indicates the Walker A motif.

Supplementary Figure 2 **IcmF3 is involved in siderophore production**. The orange halos around the colonies were measured. **(A)** Relevant to Figure 5B, **(B)** Relevant to Figure 5C. The graph shows the mean and SD of two experiments performed in eight replicates each time. ^*^*P* < 0.05; ^**^*P* < 0.01; n.s., not significant.
