# Supplementary material for: The icmF3 locus is involved in multiple adaptation- and virulence-related characteristics in Pseudomonas aeruginosa PAO1
Source: Front Cell Infect Microbiol. 2015 Oct 1;5:70. doi: 10.3389/fcimb.2015.00070 (PMC4589678; doi:10.3389/fcimb.2015.00070)
Supplement: Supplementary Table 1 — Strains and plamids used in this work. [file Table1.DOC]

**Supplementary Table 1 | Strains and plamids used in this work.**

| Strains and plasmids | Relevant characteristics* | Source |
| --- | --- | --- |
| *E.coli* |  |  |
| DH5α | FΦ80Δ*lacZ*ΔM15/Δ(*lacZYA-argF*)*U169recA1 endA1 hsdR17* | Laboratory collection |
| S17-1 | F^-^*thi* *pro hsdR* [RP4-2 Tc::Mu Km::Tn7 (Tp Sm)] | Laboratory collection |
| *P. aeruginosa* |  |  |
| PAO1 | Wild-type | Laboratory collection |
| PAO-F | *icmF3* deletion mutant | This work |
| PAO-F (pME6032-*icmF3*) | Δ*icmF3* with pME6032-*icmF3* | This work |
| Δ*pvdA* | *pvdA* deletion mutant | This work |
| Δ*pchD* | *pchD* deletion mutant | This work |
| Δ*pvdA*Δ*pchD* | *pvdApchD* double deletion mutant | This work |
| Δ*lasRI* Δ*rhlRI* | PAO1Δ*lasRI*::Gm Δ*rhlRI*::Tc | (Beatson et al., 2002) |
| **Plasmids** |  |  |
| p34s-Gm | Amp^r^; Gm resistant cassette carrying vector | (Dennis and Zylstra, 1998) |
| pK18*mobsacB* | Km^r^; *sacB*-based gene replacement vector | (Schafer et al., 1994) |
| pMini-CTX::*lacZ* | Ω-*FRT*-*attP*-MCS, *ori*, *int*, *oriT*, Tc^r^ | (Becher and Schweizer, 2000;Hoang et al., 2000) |
| pMP220 | *IncP* replicon for *lacZ* transcriptional fusions, Tc^r^ | (Spaink et al., 1987) |
| pBBR1MCS-5 | Broad-host-range vector, Gm^r^ | (Kovach et al., 1995) |
| pK-F | Km^r^, Gm^r^; Δ*icmF3*::Gm in pK18*mobsacB* | This work |
| pK-A | Km^r^, Gm^r^; Δ*pvdA*::Gm in pK18*mobsacB* | This work |
| pK-D | Km^r^, Gm^r^; Δ*pchD*::Gm in pK18*mobsacB* | This work |
| pME6032 | Shuttle vector between *Pseudomonas* and  *E. coli* containing *lacI*^q^-*Ptac* fragment for  gene expression; source of *tetA* gene cassette, Tc^r^ | (Heeb et al., 2002) |
| pME6032-*icmF3* | *icmF3* in pME6032 | This work |
| pMP-A1 | 1286 bp upstream region of *phzA1* in pMini-CTX::*lacZ* | This work |
| pMP-A2 | 1099 bp upstream region of *phzA2* in pMini-CTX::*lacZ* | This work |
| pMP-C | 1120 bp upstream region of *fliC* in pMini-CTX::*lacZ* | This work |
| pMP-L | 1146 bp upstream region of *fliL* in pMini-CTX::*lacZ* | This work |
| pMP-E | 633 bp upstream region of *fliE* in pMini-CTX::*lacZ* | This work |
| pMP-F | 760 bp upstream region of *flgF* in pMini-CTX::*lacZ* | This work |
| pMP-M | 684 bp upstream region of *flgM* in pMini-CTX::*lacZ* | This work |
| pMP-D | 684 bp upstream region of *pchD* in pMini-CTX::*lacZ* | This work |
| pMP-E | 684 bp upstream region of *pchE* in pMini-CTX::*lacZ* | This work |

*Tc, tetracycline; Gm, gentamicin; Km, kanamycin; Amp, ampicillin.

**REFERENCES**
