# Supplementary material for: The icmF3 locus is involved in multiple adaptation- and virulence-related characteristics in Pseudomonas aeruginosa PAO1
Source: Front Cell Infect Microbiol. 2015 Oct 1;5:70. doi: 10.3389/fcimb.2015.00070 (PMC4589678; doi:10.3389/fcimb.2015.00070)
Supplement: Supplementary Table 2 — Sequences of the PCR primers used in this work. [file Table2.DOC]

**Supplementary Table 2 | Sequences of the PCR primers used in this work.**

| Primer name | Primer sequences (5’→3’) * |
| --- | --- |
| PAIcmF3 up F | CGTGTCTCTAGAGATGGTCGAGCGCGCCCTCGAACTG |
| PAIcmF3 up R | CAGGACCAGCGGCAGCAGACGCCAGGTGGAGCACAAC |
| PAIcmF3 low F | GGCGTCTGCTGCCGCTGGTCCTGCAACTGCCGGTG |
| PAIcmF3 low R | CACGACAAGCTTCGCGCGCAACGCTTCGTCTAGCTTC |
| pvdA up F | CTCGGAATTCCCAGGCTCGGGGATCGAC |
| pvdA up R | GTGTCGCTGAGCTGCTTGTCCAGGAACAGCAC |
| pvdA low F | GACAAGCAGCTCAGCGACACCCTGCTGTC |
| pvdA low R | CTCGTCTAGAGGTCATCGGTCGCGATGG |
| pchD up F | CTCGTCTAGACTCATGATCCGCGGATCG |
| pchD up R | GTATTGCTTCAGGCTCTGGTCCTGCCAGTG |
| pchD low F | GGACCAGAGCCTGAAGCAATACCTGCACG |
| pchD low R | CTCGAAGCTTGGCGCTGACTTCCTCGTC |
| icmF3 F | CTCGGAATTCATGAGCGGCGCGACGCTG |
| icmF3 R | CTCGAGATCTCATGGGGTACCTCCGGTGCTG |
| PphzA1 F | CTCGCTCGAGTGGAATGCCAGGTTGCTC |
| PphzA1 R | CTCGCTGCAGGTGCCGCTGTAACCGTTC |
| PphzA2 F | CTCGGGTACCTCCATCGACCCGATCGTG |
| PphzA2 R | CTCGGTCGACGCTGTGCCGCTGTAACCG |
| PfliC F | CTCGGGTACCGGACCGTTTCGTGGTCTC |
| PfliC R | CTCGAAGCTTACTGTTGATGCGGTAGCC |
| PfliL F | CTCGGGTACCACCCTGGTCAAGCTCAAC |
| PfliL R | CTCGAAGCTTGCCGACCACGATCAGCAG |
| PflgF F | CTCGGGTACCATGCGCAAGACCACCCAG |
| PflgF R | CTCGAAGCTTCTGCTCGAAGTCGCGACG |
| PflgM F | CTCGGGTACCGCGCCCTGGACCATCTTC |
| PflgM R | CTCGAAGCTTAGCCTGGCCTGCCTTGTC |
| PfliE F | CTCGGGTACCGCCGACCTGTGCCTCACC |
| PfliE R | CTCGAAGCTTGGCCATGGCTTCCATTTG |
| PpchD F | CTCGGGTACCGCGGCTGTCCAGGGCTTC |
| PpchD R | CTCGAAGCTTGCTCTGGTCCTGCCAGTG |
| PpchE F | CTCGGGTACCCGCTCAGCGCACAGTTCC |
| PpchE R | CTCGAAGCTTCAGCAGGTCGTCGTCGTC |

* *Underlined sites indicate restriction enzyme cutting sites added for cloning.
