# Supplementary material for: The icmF3 locus is involved in multiple adaptation- and virulence-related characteristics in Pseudomonas aeruginosa PAO1
Source: Front Cell Infect Microbiol. 2015 Oct 1;5:70. doi: 10.3389/fcimb.2015.00070 (PMC4589678; doi:10.3389/fcimb.2015.00070)
Supplement: Supplementary Figure 1 — Multiple sequence alignment of the full-length IcmF protein sequences from various bacterial species. Sequences are labeled to the left by protein name and accession number, with residue numbers corresponding to the last alignable residue indicated to the right of the sequence. Red outline indicates the Walker A motif. [file Image1.PDF]

AHA_1845 YP_856379	---MFKTIFTFLRQQL	13
BB0816 NP_887365	---MIPRL	5
BURPS1106A_A2840 YP_	--MSHAVARI	8
BUBURPS1106A_A0140 Y	MSYLKRFLRFVFSWQMLACIAVLLVCAAVWFVGPLLAFDELRPLAGVVVRVAVIVLLVAL	60
BURPS1106A_A0236 YP_	---MNKFLSFLVSRSFIAFVALLAVALVIWFVGPFVAFGGLTPLAGAGMRVLAIALLLAG	57
BURPS1106A_A0717 YP_	--	1
BURPS1106A_3650 YP_0	--MQRF	4
Daro_2188 YP_285397	---MMSQI	5
c3398 NP_755273	--MKRV	4
ECs0218 NP_308245	--	1
HH0252 NP_859783	--MFGKIFNY	8
mlr2349 NP_103715	---MFILRFL	7
MXAN_4804 YP_632965	--	1
Pden_2453 YP_916236	---MRILKAI	7
Pcar_2811 YP_0067185	--MQNILIFL	8
plu2288 NP_929545	--	1
plu0359 NP_927714	---MNWLSLLLNNIKKPARPRL	19
plu3247 NP_930474	--MKVNRHHQ	8
PFL_6081 YP_263139	---MKA	3
PP_3091 NP_745235	--MKTL	4
PSPPH_0125 YP_272435	--MKTL	4
Rmet_0633 YP_582788	--	1
PA2361 NP_251051	--	1
PA1669 NP_250360	--MKNFFKKV	8
PA0077 NP_248767	--	1
Atu4332 NP_356323	--MNPLSYFYTIRSYVES	16
EvpO ABW69087	--	1
pRL120464 YP_764970	--MNPLSYFYTLRSYVEA	16
STM0285 NP_459283	---MQKFLSLLFSRRALAVVGVLVLALLVWFVGPLVSFDTLRPLASVGSRVVTIALLML	57
Acid_0223 YP_821521	--	1
YPK_0399 YP_00171915	--MKLPFFIRIAKPAI	14
YPK_1485 YP_00172023	MSYLNRLFSGWAMKRILLVGCFILLAAAIWFLGPFLGFGEARPLESYEARIICILLALLL	60
YPK_3550 YP_00172227	--	1
YPK_2591 YP_00172132	---MKRIALPIKKP	11
XOO3038 AAW76292	--MRFV	4
RRSL_01754 EAP72360	--MQRF	4
GSU3166 AAR36557	--	1
ECA3432 YP_051522	---MFKTIITFLRKQL	13
VC0395_0016 WP_00203	--	1
APECO1_1770 WP_00047	---------------------------------------MFKFPTSRLFSTLKSALRPAM	21

AHA_1845 YP_856379	PKLKPSWPLLGAVLWVLALILVWWLGPRLEVRGAKPFEPLWGRVVFTLLWLWLLLGVVSW	73
BB0816 NP_887365	FGWLFSRGLWSFLGLVALALLVWIAGPLLAIGALRPLESETARVLVIAALFAFALLRLLW	65
BURPS1106A_A2840 YP_	VRPLPSRDIWTFAGLVVLACFVWLAGPLFAFAEFRPFESGAVRAATIVALFVAWGARIAW	68
BUBURPS1106A_A0140 Y	LAFWLLRWPLSPIGVAALCLLIWHAGPLFAFGDHRPFGPAWVRVLIVAAMLFCYAVYGLY	120
BURPS1106A_A0236 YP_	VLLWLAGRPTSIVFVALLCVLIWHASPLLAFGGATPFAPEAVRAIAIALVLAAFALHLAF	117
BURPS1106A_A0717 YP_	----MIRTSLRVFAAILIAILIWWVGPLFAFGIYHPLGPVWVREILVALVLIWGFWPTLA	56
BURPS1106A_3650 YP_0	LNVLTHPRTLTIVGFAALAAVLFIAADALQIGLAWAAVALGVALALWLATKLWRRWRVRR	64
Daro_2188 YP_285397	RSFLTDSRFLSFVGVAAVAAFFFLGAKTLKVAMIWAGIASLAILAVWAVVWFVRRQRARK	65
c3398 NP_755273	VRLLGVGLLLLVVLLLILFVLAQTTPLIS-------AQDEHAVWLRLLITAIVICLLS--	55
ECs0218 NP_308245	--	1
HH0252 NP_859783	LKSKTFLYILLICFALLLSVLFYIYSPLFAFNDIYIFSSALTRIQVLIVVWLLIFFIFLY	68
mlr2349 NP_103715	WAVITSRFLWTLIGIALLSLLIWVFGPIVQVGQYAPFESDNVRIAIIAGLIILWLIWLII	67
MXAN_4804 YP_632965	--MMAYLLPLLGIGAPMFALMTYLGLTMQQAAIVAALAGLLAAGVVWLVKRIRARAAAKK	58
Pden_2453 YP_916236	FRFFLSRGFWTFVGLALLAAAIWFYGPLVSVGEAAPLESAEVRALCLGAILVLWLLILLV	67
Pcar_2811 YP_0067185	QAYLFGRLGSRILGLMLILSLIWWAGPYVGIDDTSLRIYLILGTLTLFFGIWLVRKSIAK	68
plu2288 NP_929545	----MKKLFYAIGWLSVMVILLLLSLTIAVAFSWPTIGGLLLFICLLLLLLLRVAMAFM	56
plu0359 NP_927714	PPLKATASLILALLPCLALIWLWWWGPGWQFKQTHPLDTLSARWLATVIILLLALSFIGI	79
plu3247 NP_930474	YGYFGYIFITLSLVGIFLMLIYLNKTLLE-------ERGLLKSALYAYATVFPLLLIG--	59
PFL_6081 YP_263139	FFSFVIRWVIPLLGLIALSLIIWFIGPLLEF-----LVPEGRRWALIIFIFALWIGYRVF	58
PP_3091 NP_745235	LRLLKSFWLLLPLLWLASLLICWLAAPLVPWLRHHVPEALAIISACFLLVIVLRQYQRIR	64
PSPPH_0125 YP_272435	LRLLKSFWFLVPILWLISLAVCWFFAPRISWLQGYTLEAMAIISAFYLLLIVLRQYRRIR	64
Rmet_0633 YP_582788	--------------------MAYFHGTGS-------EPAWKQMELVALIGLAAVLLRG--	31
PA2361 NP_251051	------------------------------------MSGATLFMLVLLLVLLALLLGALG	24
PA1669 NP_250360	GAFLRKTWVWTLLLVLLAALLVWFVGPLLAVADYKFWESPTSRLLSISLMFLVWGLAMVF	68
PA0077 NP_248767	--	1
Atu4332 NP_356323	YAALVGRRFLSLLWVIALCVVVWFYGYLIALGDFKPLGTVQARLIAIGIIVAVWLVYIIV	76
EvpO ABW69087	-MAAMKRLFKWIAIVVLLSVLAMLCWLLTLWQRWPAAAAWVLFLAILAGALLTRYLWRKV	59
pRL120464 YP_764970	YAGLIGRRFISIIWVAAICVVIWFYGYLAVYGDFKPLASASARLTLIGIILAAWLAYLVF	76
STM0285 NP_459283	LVLWLVNWSMSIIGISVLCLAIGFVTPLLALGDVHPFAPLWVRLTLIGFILLMYALYGLY	117
Acid_0223 YP_821521	------------------------------------MIALYLLGLVAIILVVLVGVWFYL	24
YPK_0399 YP_00171915	PRLKASIPVVLALMACAALIWVWIYGPEWQLGENYPFETLLSRWLVTAVFVLVAVCWLSL	74
YPK_1485 YP_00172023	ILGFWYGAPRFVIAAVVACAAVWVIGPFILVGDDHPLTDGIRRGIIIAIIAFITLIYGTW	120
YPK_3550 YP_00172227	----MIRRMLILIIALVLCYIVWWIGPLIAIGPYFPLGGVWVRKIIIALILFWALWPFVA	56
YPK_2591 YP_00172132	EVWFWIVALLFLLAGAVLCWLVWQHPERVGLIPGTPQRDRWLTGLVVGTGILTLCALLSY	71
XOO3038 AAW76292	LDFLRVRYLASAAILSLLAVLVWVGGPYLSLGRWQPFASVSGRLLAMAVLVLAWAGWRYL	64
RRSL_01754 EAP72360	LNFLTHSRTLSILGVIVVTIFLLLMAQTFEIGLVWAGIALGVLLALWLASALWKRWRARR	64
GSU3166 AAR36557	MSERMMKSLKWLLLASVVVLTVFLVAGIVLALDWPWWVTFCLLLLLAGIAAGAILLRSLL	60
ECA3432 YP_051522	PKLKPSWLLLGALLWVVVLVLAWWLGPRLTVGESRPLQGIWGRVVFTLVWLWLAFSYSAW	73

```
VC0395_0016 WP_00203          ------------------------------------------------------------ 1
APECO1_1770 WP_00047          PRFKVSATWLLTLAWIFLLVWIWWQGPKWTLYEQHWLAPLANRWLATAVWGLIALVWLTW 81

AHA_1845 YP_856379            RVWRKMQQLKAERQHEVVL------------------EQDPVKGLIDRQALFLDRWLQAL 115
BB0816 NP_887365              RKWREGRLNAQLLGQLRRPARPEAEAAAEP----PPSDLRELEGRFDEAVELLRKTRFDG 121
BURPS1106A_A2840 YP_          RGWRAGQLNAQLLNQLR-EAAPRPAATGDP----AQAQLDELRSRFDEAATLLKKVRFGE 123
BUBURPS1106A_A0140 Y         RLWQAVRTNDALLKRILEPSAGKPDAA-------AQANIRAVSVAVSKAIGQLKRLRGGA 173
BURPS1106A_A0236 YP_          RLLQKARTDPDFLKSLLEFGGKQPESP-------AAGRLVAVNAAIAGAIARLKGMRSGA 170
BURPS1106A_A0717 YP_          RLWARLAMSPRQ--VKVAPKAKQLDFV----------DKHLRTLDQQLKERWRKEPRGRW 104
BURPS1106A_3650 YP_0          ANRQLGDMLEQQAETGKMSAAVAEPAKRA--------ELDVLRTRLADTVKTIKTSKIGQ 116
Daro_2188 YP_285397           AGEAIGSMLEQQADVLP---KRSGAATSA--------EILALKQRMQEAVKTIKTSKLGQ 114
c3398 NP_755273               ---MCIFFLFSFRQ-------------------------NEASTISLYAQPTDIKEINTE 87
ECs0218 NP_308245             ------------------------------------------------------------ 1
HH0252 NP_859783              RPLIHLIQSLKNEKHIQYK-----------------EIKQEVTKAFRRAKRNYFIALNDA 111
mlr2349 NP_103715             AQRRAIRANRMFVAEIAAPVTEKQLTPGE-------ESVAAVGAKFGEVMAELKRRKLGG 120
MXAN_4804 YP_632965           LEGALAAQADEQATTVRPDLQP---------------EIKAMQSEFTKAVEALKASKLAR 103
Pden_2453 YP_916236           GQIRAARRNRMFVTELAAPP---PLPDTP-------DAAAEIGGKFQEILATLARSRLGK 117
Pcar_2811 YP_0067185          RRGNQFRQDLDSQTGQDQSRQ---------------LEIEELKAKMNQAVASLKSSELGS 113
plu2288 NP_929545             PEIMNKLRWLNKFWRKGNNR-----------------LEYLLYQHWRQGGYLQSWRRFRS 99
plu0359 NP_927714             KIWRRLRQLEKLKLEGELN------------------TVDPVRADITRQDRYLHHWKAQL 121
plu3247 NP_930474             ---LFSNAVWWHIRQ------------------------NKAQKK-LRPETERTADRSSQ 91
PFL_6081 YP_263139            CIIQARRNAAKVLQSLAADTPPDPDSVAT------AEELAALRQRMDEALALLKKAKLGG 112
PP_3091 NP_745235             AEHNLENLLQIEVDR-----SWNATGEFR--------DQQVLRERLKHAITMLRTDRSAG 111
PSPPH_0125 YP_272435          TEHNLENLVQIEVDR-----SLKSTGEFR--------DQQVLRDRLKHAIAMLRTDRSAG 111
Rmet_0633 YP_582788           ---PIAAAWKTVASR------------------------SNERKRHQRDPAARVGKGAGS 64
PA2361 NP_251051              WWWRTRGG-----------------------------------TEIRSFYAAVRQMEREQ 49
PA1669 NP_250360              VSWRAGVRRKAVEDSDEGQERLRRDGLIE-------DEQKELRSRFKDALRTLKTSSLYR 121
PA0077 NP_248767              ------------MQSLAEVSAPDAASVAT------AEELATLKQRMDEALALLKRAKLGG 42
Atu4332 NP_356323             TIYRGRKQDKELVDSIER----EALANRQ-------AEIGEIQTRLKEALALLRRVTKKR 125
EvpO ABW69087                RAWRTRSLEAEPVEKARAAER----------------HPMTLQRQWKAATTLLKHSQLRR 103
pRL120464 YP_764970           TTIRDRRRDKQLVDGIERDAQAEAAASQQ-------AEVGEIHGRLKEALQLLRRITKKR 129
STM0285 NP_459283             RLWRALRMDEQLLRRFLHPRGEEVPVAGE-----IKADLRTVNHIVTQAIRQLRQLRVDM 172
Acid_0223 YP_821521           KKKKAAHDGAAGGDSSAPG-------------------AEDIDALVREAEQRLAAAKLGD 65
YPK_0399 YP_00171915          KVMRRVQHLEKLQLQTKIQ------------------LDDPVSADIEQQNHYLNGWKHQL 116
YPK_1485 YP_00172023          CLVKALAKNPALLDNFIKHKKAAKPEA--------EAELSEVNAMIRNGMNYVRRIHSGI 172
YPK_3550 YP_00172227          IFFSWIFRYARAPLPKRKKKTVQLDRV----------SARFHDAMLTLQHADLSSQKTRW 106
YPK_2591 YP_00172132          AGTRLSGRKHFDELR------------------------QQAQGD-DAPLPEDNAQAGET 106
XOO3038 AAW76292              RWRRDSAKQQRLAAELVGQGARAEDASGNDSRARAAQEQVRLRARFTEAMAVLRRRRRNG 124
RRSL_01754 EAP72360           ASARLEGALEQSADK------SSAPDKRE--------EVEALRVRLAEAVKTIKRSKLGQ 110
GSU3166 AAR36557              LRRRERHFVREVIEQEKDTLSTLSDGSRT--------HLDQLAQQWKEGVGALRGSHLKR 112
ECA3432 YP_051522             LVWRRVQQMRAERHEHQVI------------------EQDPLQVYVDGQQTFLDRWLEAF 115
VC0395_0016 WP_00203          ------------------------------------------------------------ 1
APECO1_1770 WP_00047          RVMKRLQKLEKQQKQQREE------------------EKDPLTVELHRQQQYLDHWLLRL 123

AHA_1845 YP_856379            NTHLG-----------KGALYAMPWYLVLGLPGSGKSSLIHRANPANKLNPRLDTELRD- 163
BB0816 NP_887365              GGGR-----GWLQRYSRQYLYQLPWYVIIGAPGSGKTTALVNSGLDFPLAERFGKAA--- 173
BURPS1106A_A2840 YP_          ADGARKGLPRWLEQMSRQYLYQLPWYVFIGAPGSGKTTALVNSGLSFPLAEQFGRAA--- 180
BUBURPS1106A_A0140 Y         FGWR-------RLLESGRYLYELPWYMVVGAPGAGKSAAIARSGLKFPLADQMEASS--- 223
BURPS1106A_A0236 YP_          RGLR-------RLLRGKRYLYELPWYVTLGSRACGKTSALLNAGLSFPVAAQMPRAA--- 220
BURPS1106A_A0717 YP_          KRWV-------GALT-REHRTMLPWYLVLGSEGSGKSSLVAKAVSVSGSLQDRVLGS--- 153
BURPS1106A_3650 YP_0          VSGG-------------SALYELPWYIVIGNPAAGKSSAVINSGLQFPFADKNSAVIHG- 162
Daro_2188 YP_285397           VSGA-------------EALYELPWYMTIGNPAAGKSTAIVNSGLKFPFDDGGDAIIKG- 160
c3398 NP_755273               QPNY-------------ASLLTIYLRDRYGPFWRRKVRLLVTGEPEQAEAIAPGLTG-- 132
ECs0218 NP_308245             --------------------------MVIGPAGSGKTTLLREGFPSDIIY-APEGARG- 31
HH0252 NP_859783              KSMWKR----------KLYLKNIPLVIIIGNEGAGKSSFINYANIQYPLSESLTSYKKF- 160
mlr2349 NP_103715             ----------------RKFLREMPWYVIVGPPATGKTTALRQSGLNFPID---LTDD--- 158
MXAN_4804 YP_632965           GGKD--------------ALAVLPWYLIVGPPGAGKSTALRNSGLKFPYLSARGGVRG-- 147
Pden_2453 YP_916236           ----------------RRFLREMPWYVIIGPPGTGKTTALRQSGLNFPIA---LNDD--- 155
Pcar_2811 YP_0067185          HRRG------------NAALYALPWFMIIGPSAAGKTTLLRNSGLHFPYANGEDIDIRG- 160
plu2288 NP_929545             LLHR--------------NEPVPPWFLVVGERGNGKQKLLTRCNVVPMTGKNLPALTEN- 144
plu0359 NP_927714             QRHLGT----------RDYLYQRPWYLVMGNTGSGKTALIQAGCRLTDIA-APDTLSGE- 169
plu3247 NP_930474             SALV-------------LSDLGDRMRLRYGRFWWYKVRILLVVGEVEQVEAIAPGLTT-- 136
PFL_6081 YP_263139            --------------DERRNLYELPWYVIIGPPGSGKTTALVNSGLHFPLAAQLGAGA--- 155
PP_3091 NP_745235             GGGK-------------AALSDLPWYLVVGMSAAGKTSLLTHSGLSASIAT-ANDSES-- 155
PSPPH_0125 YP_272435          GGGS-------------SALYDLPWYLVIGMSAAGKTSLLTRSGLSASIASSANDTES-- 156
Rmet_0633 YP_582788           KINL-------------AALQYANLKLALREHRCRRLPRLLVTGNDAAVSRLLPELEE-- 109
PA2361 NP_251051              GWQG---------------RYEAPWLLMLGNETEGEQLCSTWRLLPVARPAWFGRWWS-- 92
PA1669 NP_250360              GRSE-------------RWRNELPWYLLIGPQGSGKTSLLDFSGLEFPINRIDRKLTR-- 166
PA0077 NP_248767              --------------SERRNLYELPWYVIIGPPGSGKTTALMNSGLDFPLAAQMGAGA--- 85
Atu4332 NP_356323             ----------------FGYIYDLPWYVIFGAPGSGKTTALTNSGLQFPLGDALGENA--- 166
EvpO ABW69087                FRHP---------------LQVLPWFMLLGRPGSGKSALLAQSGLAAPLDLPHELND--- 145
pRL120464 YP_764970           ----------------FGYIYELPWYVIFGAPGSGKTTALTNSGLKFPLGDALGSNS--- 170
STM0285 NP_459283             PGWR-------KIFEGKRFLYELPWFMVVGSPGDGKTTALLNTGLQFPLAEQMEQTSRIL 225
Acid_0223 YP_821521           R------------------LAGLPVLFLAGDTGSTKTSVMVNAGLEPELLAGLVYQDKN- 106
YPK_0399 YP_00171915          QRHLNT----------PEYLYHLPWYMVIGARNSGKSTLIKEGYKLTEIS-ASERLHVE- 164
YPK_1485 YP_00172023          PAWR-------RFFISSRWRTELPWFMVVGMKGAGKTALILSSGQDFPLPEQLNRIG--- 222
YPK_3550 YP_00172227          QRWR-------QRRK-RQYIDAKPWFLVMGPPGCGKTSIVYESSEKFLLSEQYGLAQ--- 155
YPK_2591 YP_00172132          SQGE-------------PSRLKTRLRRRYGLFWRDKVRLLMVVGEPDEIAALAPQLAE-- 151
XOO3038 AAW76292              -----------------PGLHVLPWYLLIGAPGSGKSTLLQSSGLEFPLKAHGTHAA--- 164
```

Walker A motif: G X X X X G K T/S

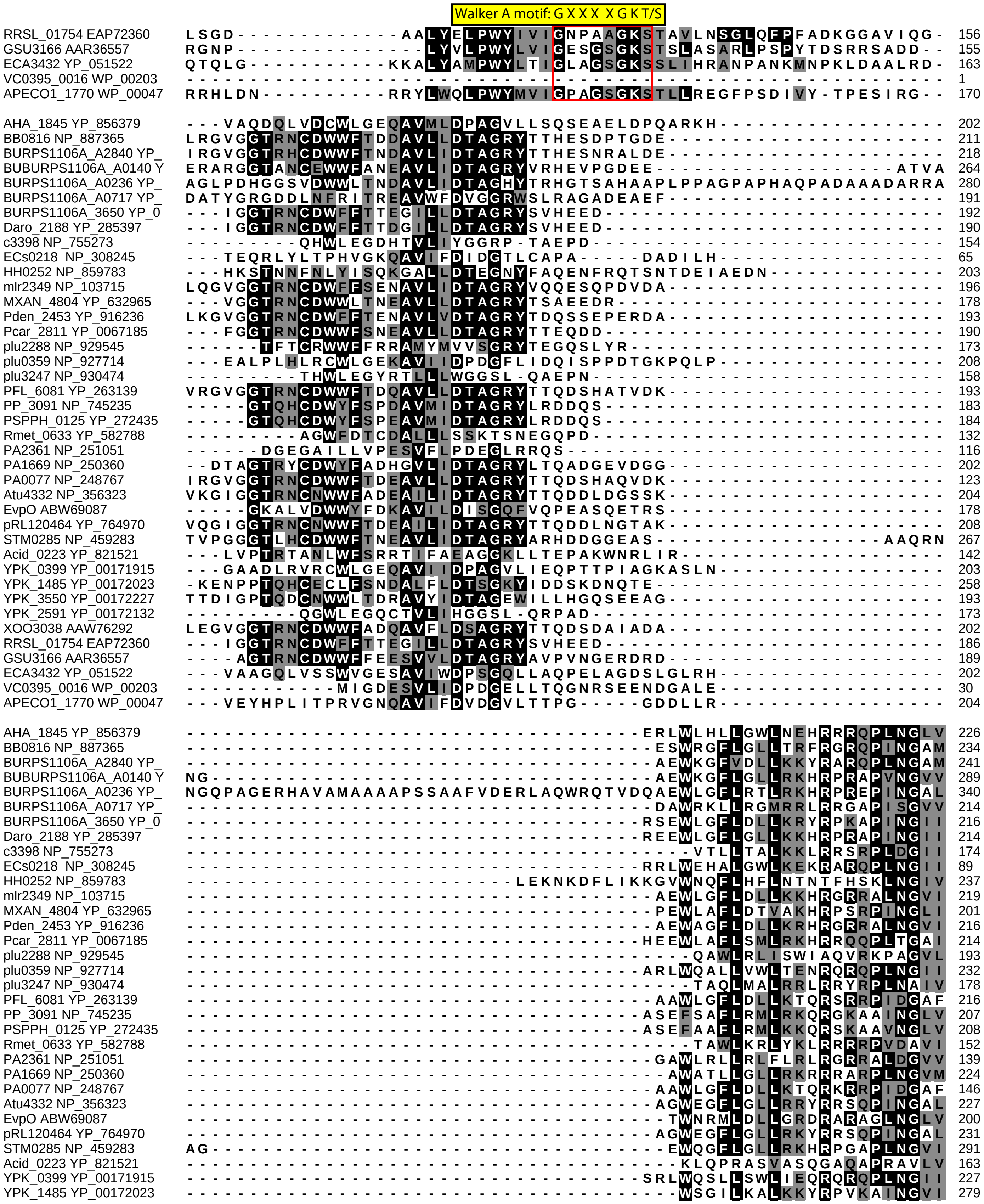

Walker A motif: G X X X X G K T/S
RRSL_01754 EAP72360 LSGD--------------AALYELPWYIVIGNPAAGKSTAVLNSGLQFPFADKGGAVIQG- 156
GSU3166 AAR36557 RGNP----------------LYVLPWYLVIGESGSGKSTSLASARLPSPYTDSRRSADD-- 155
ECA3432 YP_051522 QTQLG-----------KKALYAMPWYLTIGLAGSGKSSLIHRANPANKMNPKLDAALRD- 163
VC0395_0016 WP_00203 -- 1
APECO1_1770 WP_00047 RRHLDN----------RRYLWQLPWYMVIGPAGSGKSTLLREGFPSDIVY-TPESIRG-- 170
AHA_1845 YP_856379 ---VAQDQLVDCWLGEQAVMLDPAGVLLSQSEAELDPQARKH------------------ 202
BB0816 NP_887365 LRGVGGTRNCDWWFTDDAVLLDTAGRYTTHESDPTGDE---------------------- 211
BURPS1106A_A2840 YP_ IRGVGGTRHCDWWFTNDAVLIDTAGRYTTHESNRALDE---------------------- 218
BUBURPS1106A_A0140 Y ERARGGTANCEWWFANEAVLIDTAGRYVRHEVPGDEE-------------------ATVA 264
BURPS1106A_A0236 YP_ AGLPDHGGSVDWWLTNDAVLIDTAGHYTRHGTSAHAAPLPPAGPAPHAQPADAAADARRA 280
BURPS1106A_A0717 YP_ DATYGRGDDLNFRITREAVWFDVGGRWSLRAGADEAEF---------------------- 191
BURPS1106A_3650 YP_0 ---IGGTRNCDWFFTTEGILLDTAGRYSVHEED--------------------------- 192
Daro_2188 YP_285397 ---IGGTRNCDWFFTTDGILLDTAGRYSVHEED--------------------------- 190
c3398 NP_755273 ---------QHWLEGDHTVLIYGGRP-TAEPD---------------------------- 154
ECs0218 NP_308245 ---TEQRLYLTPHVGKQAVIFDIDGTLCAPA-----DADILH------------------ 65
HH0252 NP_859783 ---HKSTNNFNLYISQKGALLDTEGNYFAQENFRQTSNTDEIAEDN-------------- 203
mlr2349 NP_103715 LQGVGGTRNCDWFFSENAVLLDTAGRYVQQESQPDVDA---------------------- 196
MXAN_4804 YP_632965 ---VGGTRNCDWWLTNEAVLLDTAGRYTSAEEDR-------------------------- 178
Pden_2453 YP_916236 LKGVGGTRNCDWFFTENAVLVDTAGRYTDQSSEPERDA---------------------- 193
Pcar_2811 YP_0067185 ---FGGTRNCDWWFSNEAVLLDTAGRYTTEQDD--------------------------- 190
plu2288 NP_929545 -----TFTCRWWFFRRAMYMVVSGRYTEGQSLYR-------------------------- 173
plu0359 NP_927714 ---EALPLHLRCWLGEKAVIIDPDGFLIDQISPPDTGKPQLP------------------ 208
plu3247 NP_930474 ---------THWLEGYRTLLLWGGSL-QAEPN---------------------------- 158
PFL_6081 YP_263139 VRGVGGTRNCDWWFTDQAVLLDTAGRYTTQDSHATVDK---------------------- 193
PP_3091 NP_745235 -----GTQHCDWYFSPDAVMIDTAGRYLRDDQS--------------------------- 183
PSPPH_0125 YP_272435 -----GTQHCDWYFSPEAVMIDTAGRYLRDDQS--------------------------- 184
Rmet_0633 YP_582788 ---------AGWFDTCDALLLSSKTSNEGQPD---------------------------- 132
PA2361 NP_251051 ------DGEGAILLVPESVFLPDEGLRRQS------------------------------ 116
PA1669 NP_250360 --DTAGTRYCDWYFADHGVLIDTAGRYLTQADGEVDGG---------------------- 202
PA0077 NP_248767 IRGVGGTRNCDWWFTDEAVLLDTAGRYTTQDSHAQVDK---------------------- 123
Atu4332 NP_356323 VKGIGGTRNCNWWFADEAILIDTAGRYTTQDDLDGSSK---------------------- 204
EvpO ABW69087 -----GKALVDWWYFDKAVILDISGQFVQPEASQETRS---------------------- 178
pRL120464 YP_764970 VQGIGGTRNCNWWFTDEAILIDTAGRYTTQDDLNGTAK---------------------- 208
STM0285 NP_459283 TVPGGGTLHCDWWFTNEAVLLDTAGRYARHDDGGEAS----------------AAQRN 267
Acid_0223 YP_821521 ---LVPTRTANLWFSRRTIFAEAGGKLLTEPAKWNRLIR--------------------- 142
YPK_0399 YP_00171915 ---GAADLRVRCWLGEQAVIIDPAGVLIEQPTTPIAGKASLN------------------ 203
YPK_1485 YP_00172023 -KENPPTQHCECLFSNDALFLDTSGKYIDDSKDNQTE----------------------- 258
YPK_3550 YP_00172227 TTDIGPTQDCNWWLTDRAVYIDTAGEWILLHGQSEEAG---------------------- 193
YPK_2591 YP_00172132 ---------QGWLEGQCTVLIHGGSL-QRPAD---------------------------- 173
XOO3038 AAW76292 LEGVGGTRNCDWWFADQAVFLDSAGRYTTQDSDAIADA---------------------- 202
RRSL_01754 EAP72360 ---IGGTRNCDWFFTTEGILLDTAGRYSVHEED--------------------------- 186
GSU3166 AAR36557 ----AGTRNCDWWFFEESVLLDTAGRYAVPVNGERDRD---------------------- 189
ECA3432 YP_051522 ---VAAGQLVSSWVGESAVIWDPSGQLLAQPELAGDSLGLRH------------------ 202
VC0395_0016 WP_00203 -----------MIGDESVLIDPDGELLTQGNRSEENDGALE------------------- 30
APECO1_1770 WP_00047 ---VEYHPLITPRVGNQAVIFDVDGVLTTPG-----GDDLLR------------------ 204
AHA_1845 YP_856379 ------------------------------------ERLWLHLLGWLNEHRRRQPLNGLV 226
BB0816 NP_887365 -------------------------------------ESWRGFLGLLTRFRGRQPINGAM 234
BURPS1106A_A2840 YP_ -------------------------------------AEWKGFVDLLKKYRARQPLNGAM 241
BUBURPS1106A_A0140 Y NG-----------------------------------AEWKGFLGLLRKHRPRAPVNGVV 289
BURPS1106A_A0236 YP_ NGQPAGERHAVAMAAAAPSSAAFVDERLAQWRQTVDQAEWLGFLRTLRKHRPREPINGAL 340
BURPS1106A_A0717 YP_ -------------------------------------DAWRKLLRGMRRLRRGAPISGVV 214
BURPS1106A_3650 YP_0 ------------------------------------RSEWLGFLDLLKRYRPKAPINGII 216
Daro_2188 YP_285397 ------------------------------------REEWLGFLGLLKKHRPRAPINGII 214
c3398 NP_755273 ---------------------------------------VTLLTALKKLRRSRPLDGII 174
ECs0218 NP_308245 ------------------------------------RRLWEHALGWLKEKRARQPLNGII 89
HH0252 NP_859783 ------------------------LEKNKDFLIKKGVWNQFLHFLNTNTFHSKLNGIV 237
mlr2349 NP_103715 -------------------------------------AEWLGFLDLLKKHRGRALNGVI 219
MXAN_4804 YP_632965 -------------------------------------PEWLAFLDTVAKHRPSRPINGLI 201
Pden_2453 YP_916236 -------------------------------------AEWAGFLDLLKRHRGRRALNGVI 216
Pcar_2811 YP_0067185 ------------------------------------HEEWLAFLSMLRKHRRQQPLTGAI 214
plu2288 NP_929545 --QAWLRLISWIAQVRKPAGVL 193
plu0359 NP_927714 ------------------------------------ARLWQALLVWLTENRQRQPLNGII 232
plu3247 NP_930474 ---------------------------------------TAQLMALRRLRRYRPLNAIV 178
PFL_6081 YP_263139 -------------------------------------AAWLGFLDLLKTQRSRRPIDGAF 216
PP_3091 NP_745235 ------------------------------------ASEFSAFLRMLRKQRGKAAINGLV 207
PSPPH_0125 YP_272435 ------------------------------------ASEFAAFLRMLKKQRSKAAVNGLV 208
Rmet_0633 YP_582788 ---------------------------------------TAWLKRLYKLRRRRPVDAVI 152
PA2361 NP_251051 -------------------------------------GAWLRLLRLFLRLRGRRALDGVV 139
PA1669 NP_250360 -------------------------------------AWATLLGLLRKRRRARPLNGVM 224
PA0077 NP_248767 -------------------------------------AAWLGFLDLLKTQRKRRPIDGAF 146
Atu4332 NP_356323 -------------------------------------AGWEGFLGLLRRYRRSQPINGAL 227
EvpO ABW69087 --------------------------------------TWNRMLDLLGRDRARAGLNGLV 200
pRL120464 YP_764970 -------------------------------------AGWEGFLGLLRKYRRSQPINGAL 231
STM0285 NP_459283 AG------------------------------------EWQGFLGLLRKHRPGAPLNGVI 291
Acid_0223 YP_821521 ---------------------------------------KLQPRASVASQGAQAPRAVLV 163
YPK_0399 YP_00171915 ------------------------------------SRLWQSLLSWLIEQRQRQPLNGII 227
YPK_1485 YP_00172023 --------------------------------------WSGILKALKKYRPVKAINGVI 279

```
YPK_3550 YP_00172227         -------------------------------------TARQKLFSMIRKYRRYPGVDGIV  216
YPK_2591 YP_00172132         ----------------------------------------ETGLSEWRKLRRGRPLDGIV  193
XOO3038 AAW76292             -------------------------------------GAWHGFLDLLRRHR-RQPLNGVI  224
RRSL_01754 EAP72360          ------------------------------------RREWLGFLDLLKRYRPKAPINGIV  210
GSU3166 AAR36557             --------------------------------------EWQKLLALLVRHRRREPLNGLV  211
ECA3432 YP_051522            ------------------------------------ARLWQHLLQWLSENRRRQPLNGLV  226
VC0395_0016 WP_00203         ------------------------------------RRLWLHFVDWLDRTRSRRPLNGIV  54
APECO1_1770 WP_00047         ------------------------------------RRLREHWLGWLMQTRARQPLNGLI  228

AHA_1845 YP_856379           LTVDLAWLSHASVAERKAYAQLMRSRLQEVSATMNTRLPLYVTFTKLDLLRGFDVIYQQL  286
BB0816 NP_887365             LTVSIEDLLAASDAERARHAAVLRRRLQELREQLGIHFPVYVLVTKADLLAGFEQYFASF  294
BURPS1106A_A2840 YP_         LTISVADLLGASEAERTQHAMVLRKRLLELRAQLGIRFPVYLLVTKADLLAGFAEYFGGF  301
BUBURPS1106A_A0140 Y         LSVSVEELVGRTPAERTAHAATLRARLGELHQELGIHFPVYVIVTKLDLLPGFPEYFQSL  349
BURPS1106A_A0236 YP_         LAVDVATLTSADDNARHAEAAALRARLADLRAQLGVRFPVYLIVTKTDQLPGFAEYFSAL  400
BURPS1106A_A0717 YP_         LCVDGLEMIDAPLDARKRLADSVRARLEEMREAFGQQVQVYVALNGLDRLDGAVSTLSLL  274
BURPS1106A_3650 YP_0         VTASIAELTGNRPEFAINLAKNLRQRVQELTEKLEVFAPVYVMFTKADLITGFTEFFSSN  276
Daro_2188 YP_285397          VTVSIGELMGNSPSFAINLAKSLRQRVQELTEKLEVFAPVYVLFTKVDLIPGFHEFFLDL  274
c3398 NP_755273              WALTEEQSRQTAQLDKGWR------GLINGGKRLGFQAPLYLWQVCDDGDYQTGRPLQSV  228
ECs0218  NP_308245           LTLDLPDLLTADKRHREHLLQTLRSRLQDIRQHLHCQLPVYVVLTRLDLLQGFAALFQSL  149
HH0252 NP_859783             LIIDVHAFLQNPKQYEQNVLQYLSKRVSDCEENLGLQLPIYIVFNKIDLLEGMRTYWDIF  297
mlr2349 NP_103715            VALSIDALSEGD-EAIKAHGRKIRRRLAELNDRLEIRLPVYLMLTKADLIKGFEAFFGGL  278
MXAN_4804 YP_632965          VAISVSELLNADPQAVGEMGQTIRERLDEITTRLKMLVPVYVMITKCDLLPGFVEMFSDL  261
Pden_2453 YP_916236          VALSLDELLGPE-DALRRHAREIRKRLTEIAERLEMQLPVYLMLTKADRLPGFETFFGAL  275
Pcar_2811 YP_0067185         VAISLSDLLTADTQELERHVKIIRDRINELTTKLGCIFPIYIVFTKCDLLYGFSSYFADL  274
plu2288 NP_929545           VCLSVEQLLNSDNRALYIAARQVRAQLEPLQIRLERRLPIWLILTHSETLPGFDHWCTQL  253
plu0359 NP_927714           LTVDIHRLLTDNKAQRERVIAALHLRLQDLRLTLHHPLPFYLVLTKLDRLHGFSAAFQSL  292
plu3247 NP_930474           WALTENQSAQPVWMDKALR------MLQKQAHQLRWQAPVYLWQVCHSTWSQEGRITQTV  232
PFL_6081 YP_263139          VAISLSDLLLGSEAERAAHAAAIRLRIQELYTQLGVRFPIYLMLTKLDLVPGFMEFFDSL  276
PP_3091 NP_745235            LVVSLPELLAANSDERNALAAQLVARVEEYAECLDANPPVYLMLSKTDQLPGFSQAFDGL  267
PSPPH_0125 YP_272435        LVVSLPELLAASAAERNEMGARLVTRIEEYTDCLDANPPIYLMLTKTDQLPGFNQAFDGM  268
Rmet_0633 YP_582788         VVTDGAADLPMPRRGTHPYSI----RLARIIEILRYAAPVYVVDVAG-MDPTVSSDAPII  207
PA2361 NP_251051             WNIPLAR--LQDGEQAANLGLAARRRYVELTQRLGLSLPVYVVITGMEDLPGFQELLAAL  197
PA1669 NP_250360             VSIPVETLLSDDEMALDTLARQVRARLQEIQQTLHLDLPVYLVLSKADQLLGFDEFFDQL  284
PA0077 NP_248767             IAISLSDLLGSDAERAAHAQAIRARIQELYQQLGVRFPIYVMLTKFDLVPGFMEFFDSL  206
Atu4332 NP_356323            VTLSIPDLLNRDPEEQRQELRSIRQRLSELDEYLHARVPVYIVLTKADLLHGFVEFFDGF  287
EvpO ABW69087                LTVSAQTLLSADGGGVVDEGRLIRTRIEQLIRLFDRRFPVYVLVTQIDSLYGMEAWSHLL  260
pRL120464 YP_764970         VTLSIGDLLTRDPEAQREEIRAIRQRLSELDELLHARVPVYLLLTKADLLTGFVEFFDGF  291
STM0285 NP_459283           LTLNVADLTAQSPAERLAACAALRARLAELRETLGIRFPVYLVVTKMDLLPGFSEYFRTL  351
Acid_0223 YP_821521         CYDAETFTRPGAQEAATAAARNLRTRLGEMSQAFGINLPVYVLFTKMDRLPYFTEYVRNL  223
YPK_0399 YP_00171915         LTVDLHQMMTANKAQRETYVADIHQRLQEIRLSLHSQVPLYVVFTKMDLLYGFEAMYQSL  287
YPK_1485 YP_00172023         VAISASDILGKSKADLLNVSATLRTRLDEVRAVLGVRFPVYVTITKLDQLSGFDEYFRNL  339
YPK_3550 YP_00172227         LCLDVSVLLNSSVTERKSLADTLRLRILEVASTFQADIAVYLFLNNIDKLPGGETFLSLM  276
YPK_2591 YP_00172132         WAMTAAQSGTPQWMDNGLR------TLEKMGAALRYQPPVYLWQVCDSDWPQDSRTEQPV  247
XOO3038 AAW76292             VTVSVAELLELDGDAGLSHARAVRHRLNELVEKLRARVPVYLIVTKCDLVSGFAEFFADL  284
RRSL_01754 EAP72360          VTVSVSELTQNRPEFAINLAKNLRQRVQELTERLEVFAPVYVMFTKADLITGFTEFFGDS  270
GSU3166 AAR36557             LTVAADRLLAGGREENAEEGRTMRRRVDELMRALGVRVPVYVLVTKCDLIEGMNCFAELL  271
ECA3432 YP_051522            LTLDISWLAQAGVADRKAYAQVMRARLQEISVNINTRLPVYIALTRLDMLSGFDKVYRQL  286
VC0395_0016 WP_00203         LALDVAHLATATASERKAYANLLRARLRELMETLSTRLPVYIALTKLDLLHGFEPFFKHY  114
APECO1_1770 WP_00047         LTLDLPDLLTADKSRRETLVQNLRQQLQEIRQSLHCRLPVYVVLTRLDLLNGFAALFHSL  288

AHA_1845 YP_856379           DKEARDAVLGVTFKPGAD---------WQQDLALFWDQWVDNLNQNLPELMLSRLDAAQR  337
BB0816 NP_887365             SREDLEQVWGFTLPYARAQ---EPDFDLYEAFHAEYRLLQRRLDDALPEVLAAEPDEARR  351
BURPS1106A_A2840 YP_         GRAECAQVWGFTFPLAESE---APGFELRAAFDREYRLLHQRLNDGLPELLASQTDARQR  358
BUBURPS1106A_A0140 Y         TAEGRTQIWGFTLPYDAENRR-SAVGALREHCADELKRLEMRIDAGLNNRLLEEYENDRR  408
BURPS1106A_A0236 YP_         TTEGRAQAWGFTLPYDKET---IADEGVHARCADELRALAARLAAGVNTRLQEEHDTQRR  457
BURPS1106A_A0717 YP_         EASKWVKGVGFSLPDDGAHA---DAARADASWQHALQGLQQRVQQQVLYSAPAATEVSMN  331
BURPS1106A_3650 YP_0         DRQEYDRVWGATLPYEPDEK-----RDVVALFDQRFEELYDGLKEISVAQLSVSRGNKLS  331
Daro_2188 YP_285397          DWNERDRVWGATIPYDQSNG-----NELITLFDRHFDELYEGLRAMSVAQMSRLQGENAP  329
c3398 NP_755273              GCLLPER----------------------CTPEQLAVMLEAQTLPLTEQGMSQLLTDNRH  266
ECs0218  NP_308245           NRQDRDAILGVTFTRRAHE-----NDDWRTELNAFWQTWVDRMNLALPDLMVTQT--HTR  202
HH0252 NP_859783             DESIANKMLGLTLDKNSSK----------LELQNTFTELSNSLLYTFMRRNQSLHSLEDK  347
mlr2349 NP_103715            STSAREQVWGTTFPLDARV---NAG-----MIEAELARLGTELERRLVPRLEDEDKLASR  330
MXAN_4804 YP_632965          SRVERGQIWGFTVPLEQQRE------ASTDLFRERFDQMLSVLEQRSLRRLGQERRLETR  315
Pden_2453 YP_916236          TSAERAQVWGATLPVDALP---EPE-----AITREFRSLAAVLEGRLDARLAEDADLPQR  327
Pcar_2811 YP_0067185         TPKEREQVWGSWLGHEKDSN-------ICQAFIRRMETLHHRLCELRLQKLSMVRNMRAK  327
plu2288 NP_929545           SVEQHAELLGDFIEHHTVGN-------VTDVLDKSMNTIVNTLKMMR-LKLLNQQRQQPN  305
plu0359 NP_927714           DSAQRAQILGVTFSLNARD-----EQSWRTELAQFWHQWMQQLNAAMPDMMLNKVDISQR  347
plu3247 NP_930474           GCFFPER----------------------CTPDIVATQLQTLVDPLRERGMQQLLEKTAH  270
PFL_6081 YP_263139          SKEERAQVWGMTFALDDGK---SGDSPL-AHLPAELAALEQRLNERLVERLQQERDPARR  332
PP_3091 NP_745235            DLHERQQPLGMTFGLSEIRT-----NGLHAVLQTRLKKLQAHIRQHVDAQMIALG-AEAD  321
PSPPH_0125 YP_272435        DLHERQQPLGMTFGLTEITT-----ESLRSVLDRKLANLQAHVSRHVDAQMIALG-ADAN  322
Rmet_0633 YP_582788         VCALPNQ----------------------ADASAIEEALLLLRNQLARRGLDQLTQEDRY  245
PA2361 NP_251051             PEEARERALGWSSPFAAEAA------WQSRWCEQALEEITATLTESIVELGTLRG--QVD  249
PA1669 NP_250360             SREESDQVLGTSFGKEQNG-------TDVTVLRQAFEDLLRRLNSQVIMRIHQERDTLRR  337
PA0077 NP_248767             NREERAQVWGMTFALDDGK---SAEGPL-AVFDSEFALLEQRLTARLVERLQQERDPARR  262
Atu4332 NP_356323            NKTDRQQVWGTTFKLDESY---SAENLP-QRLTEEFELLQQRVDAMLIERLQQEQNAEIR  343
EvpO ABW69087                TPEALDQAMGHVVDEPENGR------DVRDVIDIALGRVNSRLKTMQLTMLLQHP--DAD  312
pRL120464 YP_764970         NKSDREQVWGTTFGLDESY---KAANLP-ERFTEEFTLLQQRVDSMLIERLQQEPNPELR  347
STM0285 NP_459283           TSHLRAQIWGFTLPYSRRRKA-GDPQALHAACAQELARLTLRLDQGLDTRLQEEYDLKSR  410
```

```
Acid_0223 YP_821521          TREESGQVLGVTIPMRDVRAGGVYGEEQTARLNADFESLFRSLADARPEFLARETDAAIQ  283
YPK_0399 YP_00171915         DKAEREAVLGVTFSLNAAD-----PDVWRTELKQFWQQWVAQLNGAMPDMMLNSVDAGQR  342
YPK_1485 YP_00172023         TAEDREQIWGVTFPYNEAKE--QSDMPLQQRISHELALLEDRISNNMHVRQQEEYAVADR  397
YPK_3550 YP_00172227         NDELLAGGLGISMTHNEKGQ---NNFVADE---ERYRELLTRVSRYVLEILHDSPNMEMR  330
YPK_2591 YP_00172132         GAVFPAK----------------------ATPEKVEQQLRALLPQLREQGMQQLSIEPHH  285
XOO3038 AAW76292             DAAGRAQVWGVSFPQAQAA---GDTDPL-TRFPTELERLLERIDQRVLERLHRARDARER  340
RRSL_01754 EAP72360         EKQERDRVWGASLPFEPDAK-----PDVAALFDQRFDELCDGLKEISVAQIALHQKNKLS  325
GSU3166 AAR36557             PEKALRQPMGMINRDLTANVG--------SFTARTMETVTERLRSLRQILHRPEARDAT  323
ECA3432 YP_051522            NRDARQAILGVTFTPQASN-----SKGWLEELERFWDEWVTHLNDNLPDMLLTQSDRSVR  341
VC0395_0016 WP_00203         TKSQREEVLGFTFSMDSVDN----LDSWLEEFASEYTQFVSRVNGMLPHAVAAPMTLEER  170
APECO1_1770 WP_00047         DKKDRDAILGVTFTRRAHE-----SDGWRSELGAFWQTWVQQVNLALSDLVLAQTGAAPR  343

AHA_1845 YP_856379            NALFSFVRQLAGLKDYVTSLLAETLAIE---ESKP-LLVRGVYVSSVYQQGVPFDAFAQA  393
BB0816 NP_887365             ALAYLLPQQFAGLQAVLGHFLSDVFASS---RFEERLVPRGVYFTSGTQGGETFDQVTGH  408
BURPS1106A_A2840 YP_       EMTYLLPQQIADLQDMLGQFVAEVFSVS---SFEPMPMLRGVYLTSGTQEGTAFDRVMSG  415
BUBURPS1106A_A0140 Y       KRLYALPQEFRSLSAALSDMLALVFLDSRYDDAQLQNTLRGVYFTSAEQTDQVLAADRET  468
BURPS1106A_A0236 YP_       RRLAALPEEFLVLTRPLGELIDRVFADSRYDDTQHHATLRGVYFTSAAQTGGEAAAETRT  517
BURPS1106A_A0717 YP_       HAQLRFVETLSRLQKALVAWLHVALAPG---EPHTAARLRGVWLGSMAELAEPHPAGAGG  388
BURPS1106A_3650 YP_0       PGQLSFPLEFSTIKPALRSFLATLFEDN---PFQYKPIFRGFYFTSALQEG-ETNSAAAQ  387
Daro_2188 YP_285397          PGLLTFPSEFASIKPALKAFIATLFEDN---PFQFKPVFRGFYITSAIQST-DIKPVSND  385
c3398 NP_755273              DFLLRLAHTLAERGIAHWQTVLKPLLAG---GAFSSLRLRGLMFSPPLAAVPEAAP----  319
ECs0218  NP_308245           ASLFSFSRQMQGSREPLVSLLEGLLD-G---ENMN-VMLRGVYLTSSLQRGQMDDIFTQS  257
HH0252 NP_859783             NLALLFLKQLDVLFALAIDFILQAKEQN---AFKNKSYIRGVYFTSAYQENVPRNYLLDA  404
mlr2349 NP_103715            AEIFRFPAQLASLRGPIQVLIEAMFGES---RYEEAAWLRGLYLTSATQEGAPIDRLTAA  387
MXAN_4804 YP_632965          EKIYGFPQKFDALRKNLAEFLQPLFLEN---VFQDTPVFRGLYFNSGTQELRPVDKVSPS  372
Pden_2453 YP_916236          AAIFRFPAEFERLEAPLRLLIDTAFGES---RYEQTPWLRGFYLSSATQEGSPLDRMLAG  384
Pcar_2811 YP_0067185         SELFDFPSQFEAAKDRLVAFISRLTEAN---PYQDTPHFCGIYLTSATQEGTPLQKILGN  384
plu2288 NP_929545            AEILALPETVAQLQPALGCYLNALFDQD---HYLEHGLLRGLFFTAEHQTAGVFSQRLLG  362
plu0359 NP_927714            SPLFSFTRQIQGLHSYLVPLLEGLLDNS---GPAQ-PGLRGLYFNSGTQELRPVDKVSPS  403
plu3247 NP_930474            DFLCRLSANLKQQGIAHWQQVLTPWLAE---YG-DVVSLRGLMFSLPLKAQTTRLP----  322
PFL_6081 YP_263139           DLIYGFPQQFAALKESLQSFLEGVFKPN---AFEERVLLRGVYFTSGTQEGSPIDRLIGA  389
PP_3091 NP_745235            STLLNFPQYFAALSGVLEQFLEHFTRGH---RGGAPLLLRGLYFTSALQNGQQLGQVYED  378
PSPPH_0125 YP_272435         SALLNFPNYFAELSAILEQFLQHFARSN---VNGTQLLLRGLYFTSALQTDKQLTPVYED  379
Rmet_0633 YP_582788          PYQARLSQRFDTR-----SKALAKWTAG---LIAGQIAVRGIAFAP-YPSGTSTKPEM--  294
PA2361 NP_251051             NELYCLPPRLESLRGSLQALLEPVFQGN---ARGEAPRFRGLYLSGSEAAGAAADEVLPA  306
PA1669 NP_250360             GRILDFPHQLGQIGERLCLFVELAFTGN---RYQRASRLRGFYLTSAPHLAQQLDEDTAG  394
PA0077 NP_248767             DLVYGFPQQFAALRECLGEFLNGVFKPN---PYEERPLLRGLYFTSGTQEGSPIDRLIGS  319
Atu4332 NP_356323            GRIFRFPAELARLKDRLHEALAELCASS---PLIEAPLLRGVYFASGTQP----------  390
EvpO ABW69087               PALLMFPTELARLAEPLKRYVYHALGDS---PYLESPILRGVFFASAVQKGNTRSGPEDQ  369
pRL120464 YP_764970          GRIFRFLAELTSLKERLHEVVTELCSGS---KLVEAPLLRGIYFASSTQ-----------  393
STM0285 NP_459283            QRLYTFPREFAALGEPLLEAIEQIFLDSKFDATQLNNTLRGVFFTSAAQQAQADAVADQLS  470
Acid_0223 YP_821521          PGAYEFPREFRKIRPAVVQFLVDICQPS---QLTVGPFLRGFYFTGVRPIVINEVAPVAP  340
YPK_0399 YP_00171915         SQLFSFTRQMQGLHDYVVQLLEGILYRG---EHAQ-PLLRGVYLTSAQQRGQMDDIFTQS  398
YPK_1485 YP_00172023         KGMYALPQDFRMLSQRVTEVLQNVFFASRFDETKFHTTLRGIYFVSSCQPADVELLNNNT  457
YPK_3550 YP_00172227         HQLLLFTESLGALQKPLFGLFEQIFPAT---PIGYTGCLRQFWFGSTQNLTSWEALYYP-  386
YPK_2591 YP_00172132         DFLLRLAQSLDQGDAARWRQRLTPWFTE---YA-ARIPLRGLMFSLPDTSASAAQVHE--  339
XOO3038 AAW76292             AAVLSFPQQLRLLQPALMDVVQTAFGRH---GYAGQPWLRGVYLSSGTQQGHPIDHVISA  397
RRSL_01754 EAP72360         PGLLSFPLEFASIKPALRSFLVTLFDEN---PFQYKPVFRGFYFTSALQEG-ATNSASAE  381
GSU3166 AAR36557             PALAFFPEEFAGLREGLTAFMEGTFREN---PYQETPLLRGVFFASGRQAGSPRSRFSGT  380
ECA3432 YP_051522            NSLFSFVRQLAGVKDYVMEVLTETLATG---EDRA-FLIRGVYVSSVYQQGVPFDAFAQS  397
VC0395_0016 WP_00203         NAIYSFTRQISGLKEILQQFFQEALASD---QFSTSALVRGAYVSSVYQQGVPTNAFDDA  227
APECO1_1770 WP_00047         SAVFSFSRQMQGTGEIVTALLAALLD-G---ENMD-VMLRGVYWLTSSLQRGQVDDIFTQS  398

AHA_1845 YP_856379            ASRRYNLP-EPIHSALRG------------------ESNTYFVRQLFSSIIFPEAHLAG  433
BB0816 NP_887365             LKRYLRIE-SGPAPAAAE------------------EGRSFFLKRLLQDVIFPEAGLAG  448
BURPS1106A_A2840 YP_       IKRFLKIEGVPPAAQTGS------------------SGRSFFLKSLLQDHIFREAALAG  456
BUBURPS1106A_A0140 Y       ILQRLKRQLGRMLGGDAGAQTRDSGA----------VSGSRGYFLRDVFQHVIVPEAHLVR  519
BURPS1106A_A0236 YP_       VARRLAAATGRAPAAAARAAQQET------------------SQSFFLHDLLTKIVIPDARLVQ  563
BURPS1106A_A0717 YP_       VGSAELPVPSRPLS----------------------------ELWTPLIRQVALERDAVR  420
BURPS1106A_3650 YP_0       RIAHRFGLDSQSLPKP----------------H--SAFSKNGFFLRDLFSKVIFADRQTVR  430
Daro_2188 YP_285397          RILRKFSLNGGGSASV----------------R--TALS-HGYFLKELFSKVIFPDRNLVR  427
c3398 NP_755273              ---------------------------------------------HAWLPSPVWAGVTGDN--ARG  338
ECs0218  NP_308245           AARQYRLGNNPLASWPLV-------------------DTAPYFTRSLFPQALLAEPNLAT  298
HH0252 NP_859783             VCEKYEIKKPPAKSATKQ------------------NKRSYFVQGLLEHIFLKDAHLSS  445
mlr2349 NP_103715            LSSSFGLPPRRAMPAARV------------------EKRSFFLRNLLTEVIFKEAGLGT  428
MXAN_4804 YP_632965          AAEIFGSTNGRAQTDGAT------------------DGRSYFLWDVFTKVMFQDQQVAV  413
Pden_2453 YP_916236          MAAGFGLAPAPVPHRAMG------------------ERRSFFLHDLLAGVIFGEAGLGL  425
Pcar_2811 YP_0067185         IRQAFGYVDEDQSSEQAET-----------------KSFFVKDFFKEVVFPNANDVS  424
plu2288 NP_929545            EYLPDQVQHSQDQRSHNVLLTG--------------WRTLTKRVCVSLLSLMLVYGVGNALM  410
plu0359 NP_927714            AAVQYHLGPQAFPTWPVA------------------DTVPYFTHALFEAVLLAEPNLAA  444
plu3247 NP_930474            ---------------------------------------------QIWLPDTAWQGVLEGSRCFHG  343
PFL_6081 YP_263139           MAQSMNLDRQHLARQTG-------------------TGRSYFIEKLFSAVAFAERGLVG  429
PP_3091 NP_745235            VIADEFALQAAYDEQAGHVG----------------KALGNRSYFITDTFRQVIFPDRDLIL  424
PSPPH_0125 YP_272435         ELSESFVLRPATAPDALEEDE-----------Q--RKISDRSYFITDTFRRVIFPDRDLTL  427
Rmet_0633 YP_582788          ---------------------------------------------PGSADLPHWRYLSEAARRQPG  315
PA2361 NP_251051             VDAPRR------------------------------RSSFASQLWARRILAEEGLAQ  333
PA1669 NP_250360             IGANLGIASGLLPTLRNG------------------RSRFIHHLLSQVIFPEANLAG  433
PA0077 NP_248767             MAQSMNLDRQHLARQTG-------------------TGRSYFIERLFREVAFGERGLVG  359
Atu4332 NP_356323            --------ETEKSPAASR------------------TRRSYFLSRLFKDVIFPEAALVT  423
```

```
EvpO ABW69087             QEIALQEAAHDTVK-------------------------RGVFVRDLFNRVLPADRHLNR  404
pRL120464 YP_764970      ---------REETVTVPR-------------------MRRSYFLSRLFKEVIFGEASLVA  425
STM0285 NP_459283        IWQRFVRAIKTARGESSASLPHALP---------DGNRSYFLHDLLTQFIFREAHLVE  519
Acid_0223 YP_821521      APQAKAGYGSAAGTTGLFQAGPAAQAPQAQPVGATRKVPQWLFLSHLFNDVLLADRAAMG  400
YPK_0399 YP_00171915     AAVQYHLAPQAFPTWPVS-------------------DTTPYFTKALFNQVLLAEPNLAG  439
YPK_1485 YP_00172023     LIQKWRNVINHKKPLSPADLSQKSDNEDRFIS--DVAYGKQYFLKQLFSDVITKDADLVS  515
YPK_3550 YP_00172227     --SSTSTFESRPAG----------------------------QMYFPALSQAITERGVLH  416
YPK_2591 YP_00172132     --------------------------------------KSWTVPASWQGGVLDDCRVARG  360
XOO3038 AAW76292        VAQHFGVAAAGLP-PVSS-------------------APRSYFLSRLLKDVVFAEAGLAG  437
RRSL_01754 EAP72360     RIARRFGLSTDGAARS---------------R--EVFSKNGFFLRDLFSKVIFSDRHTVR  424
GSU3166 AAR36557        LGTVAGPQPLPDTS-------------------------RGIFLHDFFARVLPADRALLT  415
ECA3432 YP_051522       ASRRYQLP-EPINAALRG-------------------ESNTFFVQRLFPDVIFPEASLAG  437
VC0395_0016 WP_00203    ASRRYGLSHAINTAQRAK-------------------NSTVYFTQKLFTHIIYPEAGLAS  268
APECO1_1770 WP_00047    AARQYGLGNSSLATWPLV-------------------ETTPYFTRRLFPEVLLAEPNLAG  439

AHA_1845 YP_856379        ENRLHTLYRRRRMAIGLSCLSLFSAALIGGWHYFYRVNEEAGRNVLTKAQAFMETNE--V  491
BB0816 NP_887365          RNLRWERRYRRLHWAGYGLITLAFLGLAAGWLASYGNNVRYLDEVARRVPTVDKLGRDIK  508
BURPS1106A_A2840 YP_      SNLRWHQRQRALQIVGYAAIALLCVAVLFAWLRSYSRNRDYLDEVAARVPAVDAQIGRAK  516
BUBURPS1106A_A0140 Y     PNVRWEVRFRLMRWAGHLLAVALLVWLASALTVSFDNNRGYLDAISEKTAALAARVN--A  577
BURPS1106A_A0236 YP_      PNLRWEYRSRTLSLAAHALALLLFAWVAIGLRVSMGNNDAYLDALARKTAALASRVD--Q  621
BURPS1106A_A0717 YP_      PSGPKSWRGRLGEALRWGAVPLVALSLLLWFGWGYVTERDYLDGVWAQFTEAKRLAQ--A  478
BURPS1106A_3650 YP_0      QFASPTKTR-----VRYATFFGFVAALALALGGWTWSTIG-NQQLVSNVQADLDNVTRLQ  484
Daro_2188 YP_285397       QHTTKAKAQ-----LRQFAVLGALCFLGVALGGWSWSYFN-NRSLLANVEQDIAKAVKVQ  481
c3398 NP_755273           RTVGFPWLR-----TALMSAVCVLVIWGAGMTTSFFANRA-------LVQETGIQTARAL  386
ECs0218  NP_308245        ESRAWLMRSRRRLTVFSATGGVAALLLITGWHYYNGNYQSGITVLKQAKAFMDVPP--P  356
HH0252 NP_859783          MIKTSWQTFK-----LAFGTISICIFGYVFCTYLIHKTYKEVDKSHITFNSITSLLANTS  500
mlr2349 NP_103715         FDPLAQRRAWIWRGAAAACALAALLAGGLFTWSYLDNRNAITEQAGQFEALQGPLTQVA  488
MXAN_4804 YP_632965       RSSLEEARVRRQRMMLASAASAATVLLLSLPTVSFFKNRNMAEAVTEAITSVN-------  466
Pden_2453 YP_916236       FDPRAEERRTWIWRGSAVAAALLVLLAGLGFLFNTMRQSGAVADQQRLLADLAGRLANAA  485
Pcar_2811 YP_0067185      RTKKQGILHRALKTACIITSLAVITGCFLLLSTTLTMNTLLLKKGTDTSEHLAQEAQTKN  484
plu2288 NP_929545         TTWRGVTQLENIHEADPLERSYLRYQKVEYWLQETLPSLFFYPVTHTMEQRMAQQYQQQA  470
plu0359 NP_927714         ENRRWLSRHRHTLLTFSAVGAAVVLALWSGWHYYYQQNYRAGDEVLAQANIFLSVPP--P  502
plu3247 NP_930474         RRVGMLHTQ-----TVCRGLIVLGLLWGAGMVLSFFTNRD-------QLAVVQAAVTDLK  391
PFL_6081 YP_263139        VNPKVERRRKWIARGALAGTVALVLVVSTLWIISYRANQAYIAQVDQRVGPVRQDVQNLS  489
PP_3091 NP_745235         YQSRLGRQA-----AFSPLLLGLAAATGLLFIGWQALSFANNRQWLDSLRGQLAHIEQAA  479
PSPPH_0125 YP_272435      YQSRHGRDK-----SLGPIVIALALFAGLSFIGWQALSFQKNREWLAAISQQLTELEQSP  482
Rmet_0633 YP_582788       KRIGWHPMT-----VCVGFALVAVGLWFAGMLASGWFNSR-------DVYAAQQAVTEIG  363
PA2361 NP_251051          AVPRILQLRQRWQRGIGLAALCLCLLWGGLMTWVWRDALRDAGELSQLLHGASERYQPLD  393
PA1669 NP_250360          LDRRERTRIHWGQRALYVGSLALLLLFGLLWAGGFSANHERLERLRELAQQWTQQRQ---  490
PA0077 NP_248767          TNPKVERRRKWLTIGALSATALVVLAVTAVWIASYRANQSYIAAVDQRVDPLARGIESLS  419
Atu4332 NP_356323         RDKRLSRRQLLVRRIAYAVSATAVAIVFTGWIFTYFANTQALAEADRKLGAYEQLVQGIP  483
EvpO ABW69087            PVAVTSPWRRLNRHLGLFAWWLCVAAALGYLGVSVVYWQHTLDDLRQRYPATMAMNR---  461
pRL120464 YP_764970       RDKRLSGRQLLFRRAAYASAVLLLAVVLTSWTATYIQNATALAGAERRIDAYEQLVRGVP  485
STM0285 NP_459283         PNLQWAWRYRLLRLGGHLLVLVLAFLLWQGMQTSQQTNGDYLNEISARATRLDGDVK--A  577
Acid_0223 YP_821521       AS-GSSVKAGFARRALFLAAAVLCLLLSIAFTVSFFNNRALETQARDAALGISSAES--S  457
YPK_0399 YP_00171915      ENGIWLQKTRKRMFIFSGVGALAALTLWGYWHYYHQLNYRAGEEVLTQAKTFLSIPP--P  497
YPK_1485 YP_00172023      YNLKVQSKYRFQNLLGHAACIGLAVWLVWAFLVSFQHNEGYLQATGAKLSQLESTVM--S  573
YPK_3550 YP_00172227      ATGPMPLRNRLLNIGKYVFVVLVLCILLLMLSARYFWEYDYIGYATARFEETRRIVR--D  474
YPK_2591 YP_00172132      RRVGLPWEQ-----TLCYSLLALIVLWGVGSVVSFTVNRH-------QMVSAAQQAQQLA  408
XOO3038 AAW76292         SQPGSAQRRRLLQLACWAVLTATTLGVLSGMAGSYARNVRLIGQVRDALDAYPAGPLPAD  497
RRSL_01754 EAP72360      QFASPAKTR-----LRIATFFGLVLALGLLGGWTWSYMG-NRTLTANVQADLDKIVKLQ  478
GSU3166 AAR36557         PTQRSVQWRTVTGNLGLLSWLLVGLALCGLLSFSFVKNMATIREVSRQFERTPPINADPV  475
ECA3432 YP_051522        ENRLHSLYRRRRLSIGVGCMLLASLALVGSWHHFYRVNEEAGRNVLTKAQAFIGTNE--L  495
VC0395_0016 WP_00203     DNFRVAKNKRRLMGLSFVACSVATLLLAGTWHRNYLNNVQHADTVLTKVNQYKEQFP--T  326
APECO1_1770 WP_00047     ENSVWLNSSRRRLTAFSTCGAALAALMVGSWHHYYNQNWQSGVNVLAQAKAFMDVPP--P  497

AHA_1845 YP_856379        ADAHAFGVSQLPRLNLIREATLSFGNYRERMPLVADLGLYQGDEIGPYVEGSY-LQLLSL  550
BB0816 NP_887365          ITRSGDVLGLMPFLDGLWYLPRDAAFEPDAPPLSYRFGLYQGGKMHAAMQGVY-RGTLDS  567
BURPS1106A_A2840 YP_      FTGAADIVQLLPVLDELSGLPNAGGVDLRHPPLAYRWGLFQGEKIEEASDAVY-RRALDD  575
BUBURPS1106A_A0140 Y     YNKAPKPAQIGGVLDGARDLPQHGNLDLDAPGASFRYGLYVAPGIVDASDATY-RSLLRR  636
BURPS1106A_A0236 YP_      LYKAPKPEAVPDVLTQARSLSAYPGLDLSAPGSGWRFGLYTPPGIVAESSRTY-DALEDT  680
BURPS1106A_A0717 YP_      EASYGNDGGS--TLIEIANQMRYAQLQAEDAAQGMATPYFEHGLVAETARETY-YRHLQK  535
BURPS1106A_3650 YP_0      QNRNDLQSRLQAMDILEDRIEQLEQFRRD-KPLSVSLGLYQGNRLEQHLLTEY-YNGVRQ  542
Daro_2188 YP_285397       EGRIDLQSRLEALEIIQDRLAQLEQFNED-HPLSIGLGLYQGELMADKLRREY-FAGVNN  539
c3398 NP_755273           DTRLPLAEQLVALHTLQGELERLQYRIREGAPWYQRFGLERNQQLLAAAFPGY-AQAANR  445
ECs0218  NP_308245        QGEDDYGNLQLPLLNPVRDATLAYGDWGDRS-RLADMGLYQGRRIGPYVEQTY-LQLLEQ  414
HH0252 NP_859783          SYEHLTLQEKTELMLHLKAILKNYPSLFEKPSIIQYFSLNFSYQGFLPARDFY-YAINEE  559
mlr2349 NP_103715         ATPAAVEQPTM-DGALAAMDAVATARTPPPDAVHNLLGPTASPELVRAQTDTY-DHALRN  546
MXAN_4804 YP_632965       LDPRDDIRRVEDLIPLRNRLQELTEYEEGSAPVFMRFGLYQGQKLLPQARQFY-NAALRR  525
Pden_2453 YP_916236       SRQAPTEPLDL-PVALDAMTEIEAARTPVPGGPLALIGPSAAAEIEGAARLAR-ERGLRN  543
Pcar_2811 YP_0067185      NGVNQDFKALTQVLAHYRTLMN----YEKHLPLSYTLGIYEGDKQLPAIRSIL-LNAFKR  539
plu2288 NP_929545         PVGAFNPESVAAHLLARFHNAETPQKRLLVLHWARFINIEQAMTHGATLETLQRMPAYSA  530
plu0359 NP_927714         TGEDRLGNLQLPLLNPLREATLAYGDYHQKG-LLADMGLYQGAAVGPYVENTY-LQLLSQ  560
plu3247 NP_930474         VQPENSDAQLMALKDLRNEVDRLQDRAEHGVPWYERFGLSQNQKLLTAVLPDY-AQVNNR  450
PFL_6081 YP_263139        PAQR-DVLAVLPLLNATRHLAG------DAPGWAEGLGLYQGDMLEAESASVY-RKLLVA  541
PP_3091 NP_745235         DREQQLAA-GKGLEVLREQMANVKAHRLQGVPLQLGGGLYQGEAIHQVTRSAY-LAQLRS  537
PSPPH_0125 YP_272435      DRAQRLAS-GQGLELLRNQLAAIEKYRTKGVPLQLSGGLYRGDDIYFATQTAY-LQQLRT  540
Rmet_0633 YP_582788       SAR-NPATRLHALLALQQQIERYEYRTQQHAPLLTRFGLNRDADVLAALWQPY-VKASRE  421
PA2361 NP_251051          DDTRRAAQVRQNVQAWWQLVSQAPRWRFTSLAFPSSWFSSLDARIDNAYRRVS--ERLLV  451
```

```
PA1669 NP_250360          ALGERDDAMAALKTLDSSYAATTVFPPKGEVSLHERAGLYQGNEVNPPLAAAY-RRELET 549
PA0077 NP_248767          PAQR-DVLAVLPQLNAVQNLAG------DAPSWAEGYGLYQGDMLGEESASVY-RKLLIA 471
Atu4332 NP_356323         VREVADADFLR-ILPALDNLRDVNSGFARERVWNVSFGLDQEDKIAGRQRDAY-QRALNA 541
EvpO ABW69087            -----ALPHDMQMMIAQRDAIYWLENRNAQWASRVLAFQHQLLSVEADAKSQY-VNDFSQ 515
pRL120464 YP_764970     VRDVSDTDFLR-ILPALDNLAAVTTDFSKTRVWPISLGLDQEGKVASRQREAY-QRALNT 543
STM0285 NP_459283        YTGKPAMAPVPALLDSARELSAWPELDPDAPPLAWRYGLYSVPPVTDSVASLY-NRLLDQ 636
Acid_0223 YP_821521      GQNLASLDALRKLEALRQSLETLTTYRREGEPWSYRWGLYTGDSLYPEVRRIY-FDRFHQ 516
YPK_0399 YP_00171915     EGDDRYGNLQLPLLNPIRDATLAYGNYHERSPFLADMGLYQGNNIGPYVESTY-LQLLQQ 556
YPK_1485 YP_00172023     YAKTSNEQILPALLNATQQLPEYGTLDVANPELDWRYGLYTGSAISQGANTLY-QFFLQK 632
YPK_3550 YP_00172227     IP-ITNQVND--NLVAAYEQLGYINTHYLESSPPLLTPYFEHRLLNQAMLQTY-HRHLNK 530
YPK_2591 YP_00172132     QSHAVSDQQLMALQALRNDIGRLQARVAQGAPWYQRFGLDHNAPLLEVLMPWY-GQANNR 467
XOO3038 AAW76292         AGEAAFYAKALQRFDALAQARDAALPPGASIPWSRRFGLHQGTALARDVQEAY-LRDLNG 556
RRSL_01754 EAP72360     QNRVDLQARLEALDILQDRIEQLERFRSE-HPLALSLGLYQGDALQHKLLDEY-YNGLRQ 536
GSU3166 AAR36557         NNLLVLESLRQGILRVE---------EQNRAWWVPRFGLNESRRVEAVLKDKF-CNQFRD 525
ECA3432 YP_051522        EGQPGYGYQQLPRLNLIRDATLSFGNYHERTPLLADLGLYQGGKIGPYVEGTY-LQMLNQ 554
VC0395_0016 WP_00203     SRSLASQREVLDPLNKIREATLEFGFFRDKPQYISDFGLYQGHTIGPKVEETY-LNLLET 385
APECO1_1770 WP_00047     QGTDEFGNLQLPLLNPVRDATLAYGDYRDHG-FLADMGLYQGARVGPYVEQTY-IQLLEQ 555

AHA_1845 YP_856379       RFLPAQMQGLLADLNQAPAGS--------------------------------------- 571
BB0816 NP_887365        VLLPQVARRIEAALRAARADDL-------------------------------------- 589
BURPS1106A_A2840 YP_  VLLPIAASRMEQALRDARPDEV-------------------------------------- 597
BUBURPS1106A_A0140 Y  SLLPQIVRRVEDALSAQIDAKHA------------------------------------- 659
BURPS1106A_A0236 YP_  LLLPRIVTRIEAVLSQAIADRDP------------------------------------- 703
BURPS1106A_A0717 YP_  MLMPELYNEVRRTLVSQVDGSPG------------------------------------- 558
BURPS1106A_3650 YP_0  ILLAPVSDNLASFLKDVNAHPDQLAPMTRAPESGA------------------------- 577
Daro_2188 YP_285397      VMLIPVKDNLEAFLNEVNAQGDRLKPQG-------------------------------- 567
c3398 NP_755273          LVRDVAVDHLQQQLNAFVALPPN------------------------------------- 468
ECs0218  NP_308245       RYLPSLFNGLVKAMNAAPPES--------------------------------------- 435
HH0252 NP_859783         VIGKTLLREMEQILLHNTDQT--------------------------------------- 580
mlr2349 NP_103715        VLEPHMVALLEATMWRQIRDP--------------------------------------- 567
MXAN_4804 YP_632965      VLLGKQFELIQQRLDTFGKNPDLLTVRS-------------------------------- 553
Pden_2453 YP_916236      LLEPRMVAFLEATMWRQIRDP--------------------------------------- 564
Pcar_2811 YP_0067185     IFFKPAIKDLEMRLVNYSQVWAAADLQR-------------------------------- 567
plu2288 NP_929545        SLLSGENDPLSAEQRVALRLAQYR------------------------------------ 554
plu0359 NP_927714        RFLPALMNGLLDDLNRAPKGS--------------------------------------- 581
plu3247 NP_930474        LIRDKAAAVLHAKLSALVNLPPG------------------------------------- 473
PFL_6081 YP_263139       VFAPRLMTRIEEQLHSGGNS---------------------------------------- 561
PP_3091 NP_745235        QALEPIARSLQVQMRAFNTFANGINQELEFTPAPQ-PKKRNGKP---------------- 580
PSPPH_0125 YP_272435     QALEPITLKLQLQMREFNEFAKTMDPQYGFGATPAGSETKTGKVRAQGQKLLDRGARNVA 600
Rmet_0633 YP_582788      LLVTPVQQNLEATLVDLGQLQTTT------------------------------------ 445
PA2361 NP_251051         RPLRSLLEGEASDLRAIRSDGQPGLKEGDDPSQWKDYLAAKDLVARATTLERHN------ 505
PA1669 NP_250360         QLLPRVARMLEAQIRGNMQNR--------------------------------------- 570
PA0077 NP_248767         VFAPRLVTRIEEQLRSGGSS---------------------------------------- 491
Atu4332 NP_356323        LLLPRMIVQLQKQLK-DEKDV--------------------------------------- 561
EvpO ABW69087           YILPSLDKAIQQSTTADSIASHP------------------------------------- 538
pRL120464 YP_764970    LLLPRMLVQLQKDMT-ETTDV--------------------------------------- 563
STM0285 NP_459283       LLLPPLVKRMEYVLADAIARQDS------------------------------------- 659
Acid_0223 YP_821521     LLFLQTQSAMLENLRGLPVGTQGP------------------------------------ 540
YPK_0399 YP_00171915    RFVPALMSGLLEQLNAAPKGS--------------------------------------- 577
YPK_1485 YP_00172023    YLFPMVEHQSEVALRKTMHSGQS------------------------------------- 655
YPK_3550 YP_00172227    VFWPAVQNYVTNELKKDTQSPDG------------------------------------- 553
YPK_2591 YP_00172132    ILRDATAQSLYQKLSELAELPAN------------------------------------- 490
XOO3038 AAW76292        ALLPALAQSLRRKLEQSSNDP--------------------------------------- 577
RRSL_01754 EAP72360    VMLKPVGGALETFLADVNAHPDQLAPMVRPPETGA------------------------- 571
GSU3166 AAR36557        GFLAPYDRHLAEELNGLSAATP-------------------------------------- 547
ECA3432 YP_051522       RFLPAVMQGLLEDLNQAPANS--------------------------------------- 575
VC0395_0016 WP_00203    RFLPLLMADTIVALNQAETD---------------------------------------- 405
APECO1_1770 WP_00047    RYLPSLMNGLIRDLNIAPPES--------------------------------------- 576

AHA_1845 YP_856379       ------------------------------------------------------------ 571
BB0816 NP_887365        ------------------------------------------------------------ 589
BURPS1106A_A2840 YP_  ------------------------------------------------------------ 597
BUBURPS1106A_A0140 Y  ------------------------------------------------------------ 659
BURPS1106A_A0236 YP_  ------------------------------------------------------------ 703
BURPS1106A_A0717 YP_  ------------------------------------------------------------ 558
BURPS1106A_3650 YP_0  -------------------------------TPAGPMP-----VSTHPAAPGAAPSPAVA 601
Daro_2188 YP_285397      -----------------------------------------------AAVANQAAKPARS 580
c3398 NP_755273          ------------------------------------------------------------ 468
ECs0218  NP_308245       ------------------------------------------------------------ 435
HH0252 NP_859783         ------------------------------------------------------------ 580
mlr2349 NP_103715        ------------------------------------------------------------ 567
MXAN_4804 YP_632965      ------------------------------------------------------------ 553
Pden_2453 YP_916236      ------------------------------------------------------------ 564
Pcar_2811 YP_0067185     ------------------------------------------------------------ 567
plu2288 NP_929545        ------------------------------------------------------------ 554
plu0359 NP_927714        ------------------------------------------------------------ 581
plu3247 NP_930474        ------------------------------------------------------------ 473
PFL_6081 YP_263139       ------------------------------------------------------------ 561
PP_3091 NP_745235        ----------------LAPAIAARAKAALGNTRAGAYAAKVNLATASDGAELSAATGGLS 624
```

```
PSPPH_0125 YP_272435          AGRPTSLSSIPRSTGEVANRLTGAARGTASQTRSEAIAALRNPATANDAAELSATTGGLS 660
Rmet_0633 YP_582788            ------------------------------------------------------------ 445
PA2361 NP_251051              ------------------------------------------------------------ 505
PA1669 NP_250360              ------------------------------------------------------------ 570
PA0077 NP_248767              ------------------------------------------------------------ 491
Atu4332 NP_356323             ------------------------------------------------------------ 561
EvpO ABW69087                 ------------------------------------------------------------ 538
pRL120464 YP_764970           ------------------------------------------------------------ 563
STM0285 NP_459283             ------------------------------------------------------------ 659
Acid_0223 YP_821521           ------------------------------------------------------------ 540
YPK_0399 YP_00171915          ------------------------------------------------------------ 577
YPK_1485 YP_00172023          ------------------------------------------------------------ 655
YPK_3550 YP_00172227          ------------------------------------------------------------ 553
YPK_2591 YP_00172132          ------------------------------------------------------------ 490
XOO3038 AAW76292              ------------------------------------------------------------ 577
RRSL_01754 EAP72360           -------------------------------VIATSVTSATATVATSGAAANAPASPAAT 600
GSU3166 AAR36557              ------------------------------------------------------------ 547
ECA3432 YP_051522             ------------------------------------------------------------ 575
VC0395_0016 WP_00203          ------------------------------------------------------------ 405
APECO1_1770 WP_00047          ------------------------------------------------------------ 576

AHA_1845 YP_856379            -----------------EEKLAILRVMRMLDD---------------------------- 586
BB0816 NP_887365              -----------------EYGYEALRAYLMLY----------------------------- 603
BURPS1106A_A2840 YP_          -----------------EYAYAALKAYLMLY----------------------------- 611
BUBURPS1106A_A0140 Y          -----------------DEVYRTLTVYLML------------------------------ 672
BURPS1106A_A0236 YP_          -----------------KAAYDALRVYLML------------------------------ 716
BURPS1106A_A0717 YP_          -----------------D-IYQTLKVYLMLC----------------------------- 571
BURPS1106A_3650 YP_0          QGRQQGGLYNDASPTNVEDAYNALKTYLMLSD---------------------------- 633
Daro_2188 YP_285397           ------KQYKDATPSDVEDGYNALKTYLMLSS---------------------------- 606
c3398 NP_755273               ---------SPQRTATGEQRYKQLKALLMTSR---------------------------- 491
ECs0218 NP_308245             -----------------EEKLAVLRVMRMLED---------------------------- 450
HH0252 NP_859783              ------------------TLIETIYMYQSLYD---------------------------- 594
mlr2349 NP_103715             -----------------DFMLGALKTYRMMT----------------------------- 581
MXAN_4804 YP_632965           ------DEDYSKHFDAYRQYFDDLKMYLLVTTPRD------------------------- 582
Pden_2453 YP_916236           -----------------DFLLGALKAYQMLT----------------------------- 578
Pcar_2811 YP_0067185          ------------QDQIRDPYYKTLKCYLMLSTPKR------------------------- 590
plu2288 NP_929545             -------------QGNAQQAFDQWRNVLQTLLE--------------------------- 574
plu0359 NP_927714             -----------------EEKLAVLRIMRMLED---------------------------- 596
plu3247 NP_930474             ---------SPLRAKWAQAGHDQLKAYLMMAQ---------------------------- 496
PFL_6081 YP_263139            -----------------DFLYEGLKAYLMLA----------------------------- 575
PP_3091 NP_745235             LSEEMLGRLDEQQVASIIEAYNTLKLYLLLTEP--------------------------- 657
PSPPH_0125 YP_272435          LSEEMLGRLDERQVASIIESYNALKLYLILTQP--------------------------- 693
Rmet_0633 YP_582788           --------LDDKTSQWALSGRDDLKAYLMLAY---------------------------- 469
PA2361 NP_251051              ----------RLFAQAIDNRRTPLDDLLQLSNDALGSSYNAGSLARLAYYNRTLFAERPA 555
PA1669 NP_250360              -----------------ERLLNSLRAYLMLNLR--------------------------- 586
PA0077 NP_248767              -----------------DFLYEGLKAYLMLG----------------------------- 505
Atu4332 NP_356323             -----------------TRTFNSLKLYGMLG----------------------------- 575
EvpO ABW69087                -----------------DGLVNAIQVEVRRAN---------------------------- 553
pRL120464 YP_764970           -----------------TRTFDALKLYGMLG----------------------------- 577
STM0285 NP_459283             -----------------KAAYDALRIYLLLNLDK-------------------------- 676
Acid_0223 YP_821521           ---------------EYGPTYDALKAYLITTSN--------------------------- 558
YPK_0399 YP_00171915          -----------------EEKLEILRVMRMLED---------------------------- 592
YPK_1485 YP_00172023          -----------------EEVYAALKLYLMLT----------------------------- 669
YPK_3550 YP_00172227          -----------------D-NYDTLKVYLMLG----------------------------- 566
YPK_2591 YP_00172132          ---------SPQRAARAKTGYDQLKAYLMMAR---------------------------- 513
XOO3038 AAW76292              -----------------QRLYPLLKGYLMLG----------------------------- 591
RRSL_01754 EAP72360           ATAATGKRFTDASPTNVEDGYNALKTYLMLSD---------------------------- 632
GSU3166 AAR36557              -----------------DELFARYAIHLTRRIN--------------------------- 563
ECA3432 YP_051522             -----------------EQKLTILRVMRMLDD---------------------------- 590
VC0395_0016 WP_00203          -----------------EEKLAVLRVYRMLVD---------------------------- 420
APECO1_1770 WP_00047          -----------------EEKLAVLRVVRMMED---------------------------- 591

AHA_1845 YP_856379            ---------ASGRNKELVAQYMASRWQKAFPGQGAVQEQLMGHLDYALDHTNWYGARAER 637
BB0816 NP_887365              --------EADHYDAEFMHAWLLSDMQGTLPEGYTRRQYDLMSLHLRQLAQGGVLASP-- 653
BURPS1106A_A2840 YP_          --------DSAHYDPAFVQAVVDLEMERALPADFSSAQRSALRAHLGALFGNRVAVSP-- 661
BUBURPS1106A_A0140 Y          -------DDAARHDAKAVKDWVMRDWERS-DSAAEMGGRNLMARHLDALFVDARPFEPS- 723
BURPS1106A_A0236 YP_          -------YDRARFDAAQVEAWVLDDWART-DSAAVFGGRASMIAHVEQLFRGERIVQSP- 767
BURPS1106A_A0717 YP_          --------RPARRSADDVVRWLDGRWDALSGGQYSDDDRRSLLAHVRTLMS--LKEVPA- 620
BURPS1106A_3650 YP_0          ---------KRHVEQAHLTDQVARFWRGWLETNRGNMPRDEMIRSAERMITFYLSRVSDN 684
Daro_2188 YP_285397           ---------HEHVDVGHLSDQMTRFWRGWLEANRGTMTREQMIRSAGKVLTFYLEHATHP 657
c3398 NP_755273               ---------PEKADAAFFSTTLMADGL-----RYENIPEGVRQSVLPSLLTFWTANLPEH 537
ECs0218 NP_308245             ---------KSGRNNEVVKQYMAKRWSEKFHGQRDIQAQLMSHLDYALAHTDWHAERQAG 501
HH0252 NP_859783              ---------EAYINKPLLANWVIKNWQPFAKYKIPKDDFITAIEDLAIGNLSHTQPK--- 642
mlr2349 NP_103715             --------GLSQMDTDFVQNWWVNSLPQFAPAPPFPTADAEEHQIAAIRRMA-VDDSY-- 630
MXAN_4804 YP_632965           ----PREPELDELQRKWLQDEIVKHWKRVRGDAVDERAVANHAETFLLMLANEQMLPEER 638
Pden_2453 YP_916236           --------GLGPYDRAWLAEWWQEVLPAFAAETPFPTEAARLWQLAALDRLG-GDESR-- 627
Pcar_2811 YP_0067185          --------LEAEFAAPVLQTLIQERLMGFAKGQSLDKDSQRQLSELIHLYLSHMQIDQNH 642
plu2288 NP_929545             ----------DSNNWQWLLADEQPTGTHSLTLADFWPLASDDVATINTRIRGIYTQAGE- 623
plu0359 NP_927714             ---------GSGRHKSLVEHYLQARWSQAFNGQRPLQAQLLTHLDYALDHTDWRAAREAG 647
```

```
plu3247 NP_930474           ---------PEKVDAVFLTRILDENEP-----ERAGIAPAVWLDMAWDLWSFYAENLAAH 542
PFL_6081 YP_263139          --------DSEHYDPDFIKAWITLDWDHTLARDLPPEQRQALTGHLQALFEK--HPPN-- 623
PP_3091 NP_745235           ---------QAHPDPAFVAASLPQAWASAAG--EGTPADAGVIAENA---PLYVQLLEQG 703
PSPPH_0125 YP_272435        ---------TTHPETEFVNAALPVAWAGISS--ETNPISDDVIEDNS---PVYVQLLKNG 739
Rmet_0633 YP_582788         ---------PERTEADFLAGRLPHDWS-----TEARITPGEKQALAERLFKFYAQHLKAN 515
PA2361 NP_251051            DLAALDLGRVAATAADNFHDLMARWLNAYFLTDSLERTGNALTQELAQLGQERDATAAQL 615
PA1669 NP_250360            ----------ERRENGWLKDWVATDWSQR------YAGNGTVQNGLNTHFARLLEQPFS- 629
PA0077 NP_248767            --------SPDHYDADFIKAWISLDWERNLPRDLSPEQRQALHAHLDALLER--RPPS-- 553
Atu4332 NP_356323           --------GMGGLDRDFLTTQTHQMFASLYPGDGRAAAREALDQHAKALADG-VLAP--- 623
EvpO ABW69087              ------LLRARLRGSSYGALMAMPPIPTPPLRLLYPALDSDTVSRFNDLFLAQLAWSTD- 606
pRL120464 YP_764970         --------GLGPVNADFAALEAEDMFTRLYPDEGRAAARQALIAHADVMARG-ALPP--- 625
STM0285 NP_459283           -------DHEDKYNAAEIQSWVINDLGNS-DSVAGFGGRAAVLTHIEALFDGSRVVHSP- 727
Acid_0223 YP_821521         ---------HDKSTKLFLTPVLTKWWSGNRGPDAERIALAQKQFDFYATELKEENPFAK- 608
YPK_0399 YP_00171915        ---------GSGRNAALVEQYMSHRWSQQFNGQRELQEQLSSHLNYALKHTDWHGARESG 643
YPK_1485 YP_00172023        --------GEGKFDRQYMIEHVTQDWQKS-GKIEPYEEKSIFVGHLNTLFTLKDWRQYG- 719
YPK_3550 YP_00172227        --------QPDHRSQEALVQWFMSRWESFAPQGYGQNDRKLFSYHLSELFSDTASAAPK- 617
YPK_2591 YP_00172132        ---------PEKADAVFYAQVMQTTEP-----TRAGVSPGLWQSLAPDLWQFYAQNLPAQ 559
XOO3038 AAW76292            --------DPTRRDAMHLATLADTVWRQIFPDDAS--VRAGLNLHLRALLGPVDGARA-- 639
RRSL_01754 EAP72360         ---------KRHVEVAHLTDQITRFWRGWLEDNRGNMPREQMIRSAERNLSFFLARVNDD 683
GSU3166 AAR36557            ------ILTASRAGRGLDELRALPQPASLSFMAAETPSVADAHKRFGELYLHYLAWRSDS 617
ECA3432 YP_051522           ---------ASGRNKVLVEQFMAQRWQKAFPAQGNVQEQLMQHLDYALEHTDWHKAREQK 641
VC0395_0016 WP_00203        ---------KSGRYQDYVMDYFAKYWQKSFSGQRQIQEELLGHLDYAMRHTDLTAERLNG 471
APECO1_1770 WP_00047        ---------KSGRNNEAVKQYMARRWSNEFHGQRDIQAQLMVHLDYALEHTDWHAQRQSS 642

AHA_1845 YP_856379          DQAAI--TAFVPFKEPVYGAQRELGKLPMYQRVYQNLVVKAGD-VLPPDLNVRDEVGPTF 694
BB0816 NP_887365            ---------FPRDEALVRQARDELARHTLAQRAYSRLRRMLAGADDQPPTTAVTLGGPQA 704
BURPS1106A_A2840 YP_        ---------FPMNERLVADVRERLRQVPFSQRLYRQLARTLHASTASYDFSVARAVGPDA 712
BUBURPS1106A_A0140 Y       ---------GHRNAALVQRARLFLNANPAPRRLYERAMAAIEK-EAPENFTLARAVGLQG 773
BURPS1106A_A0236 YP_        ---------LIRNDALIRQARAFLDGSNATERLYERAKAAMDK-EAPDEFTLLRAIGPQA 817
BURPS1106A_A0717 YP_        ---------TPEDANLVRSARAKAAQIPLVTRVLQHIHAQGLP-QQVNDISLSRAAGFEA 670
BURPS1106A_3650 YP_0        DW-----PMIEANLALVDQTRENLRRVVRGMPARQRVYEEIKA-RASTRFAPMTIARIVG 738
Daro_2188 YP_285397         EW-----PVIDNNLVLVDEVRDKLRQVVRGMPAAERVYAEIKA-RASTRFAPLTVANIVG 711
c3398 NP_755273             PQ-----WKTSPPPELTGAVRKILLRQIGVRNAENTLYQNVLQ-QVSRNYADMTLADMTG 591
ECs0218 NP_308245          DGDAI--SRWTPYDKPVVSAQKELSKLPVYQRVYQSLKTRALG-VLPADLNLRDQVGPTF 558
HH0252 NP_859783            ------------DTQALHIAKTQIMQLEQQQRIYTIMAFRNSL-KKQSFFNLKDKVGTAF 689
mlr2349 NP_103715           ---------IAPDKALVAEALKTVCTISLPERAYKQLLADPEV-AALKEWVPANFAGPNG 680
MXAN_4804 YP_632965         KPAREQRIAFGRVTGVVQSARRSLNNVPLVRLELAQLVANMSG--AYPDVTLEQLVGSVP 696
Pden_2453 YP_916236         ---------IEANPQLVEAALQAICTVPLSLRTYRALMSEPEI-ATLPDWIPAEKAGPLA 677
Pcar_2811 YP_0067185        PDYMET---VKLEASLVTDAQNHLKAIPDAGRLYTQILNKGKL--IAPALHLKDMIKGPG 697
plu2288 NP_929545           ----------QDIQATLGELAQALNRPAFFTPLQRSFYQQYQNQRQSEWLAFAQAMPQGE 673
plu0359 NP_927714           DQAAI--SRFAPYQLPLINAEKELSQLSIYQRVYQNLRIKAQD-SLPPPLNLRDQIGPGF 704
plu3247 NP_930474           PE-----WRITPDKALIKQVRQLLLLQLGKHNAEAALYQQMLQ-SVEKNYSDLTLRQMTG 596
PFL_6081 YP_263139          ---------ARLDQRLIDDLRRQLQQLPVAQRVYDRVKRAKLP-DGVPDFRLSEAGGRDA 673
PP_3091 NP_745235           QA-----PSLPRNEQLINETRQNLKSFMISSSLVDREYLRLQL-ESSRQFPALSLNDLVP 757
PSPPH_0125 YP_272435        DA-----PAMPRNEQLIDETRKSLKFFMISSSLVDREYLRLQL-ESSRQFPAIGLNDLVP 793
Rmet_0633 YP_582788         PD-----WKIEPRPELVAGARQTLLAVIGARSAQETLYRGILN-GAGNKYPDLTLPSLTA 569
PA2361 NP_251051            LGIGELINHLQQQVNRINLIWGNGVGQDLVPGYQNLLKSAQQSSLLGNRVEELQNLTVQL 675
PA1669 NP_250360            ---------YPLNETLVAQARQVLRSESLANVVYRVLREQARS---LPEYRFGQHLGPQA 677
PA0077 NP_248767            ---------ARLDQDLVEDLRRQLQQLPVAQRVYDRVKRQRLP-KDVPDFRISDAAGRDA 603
Atu4332 NP_356323           ---------IELDARLIATARETIRDQAIGTRAYDILAGLPQV-RELMEWTPATAFGPLG 673
EvpO ABW69087              ---------IQQQNARLLHLQARLRQLAAQSATLDWIIPWANAQPDLTNVTLDDFWQGSL 657
pRL120464 YP_764970         ---------IELDKALIAKAREVIRSQNIANRAYDILAEYRES-RALPAWSPAGALGPLG 675
STM0285 NP_459283           ---------YEKDEALIRQARAFLDGHTSTERIYARALAAMES-EAPQEFTLVRAVGADA 777
Acid_0223 YP_821521         ----------ENDAVAIERARAYLKQFAGAERVYSFMLAEAGK--TNPPIDFNRQFPGSA 656
YPK_0399 YP_00171915        DQYAI--KSFVPYLSPIQSAQQELSKLSIYQRVYQNLRIKAQD-ALPPALDLRDQIGASF 700
YPK_1485 YP_00172023        ---------QPADSELIKQARAILAEKTVTARLWGRIQSRLLA-DAPANMTLRKMVGEHA 769
YPK_3550 YP_00172227        ---------VKLNDELIRESRIKAMKTPIHIRVINRIHDAPLP-ARIEDLSLADAAGPSV 667
YPK_2591 YP_00172132        PD-----WKITADTGLISQVRQVLLGQMGQRNAESTLYENMLL-SVRRNYADMTLMDMTG 613
XOO3038 AAW76292            ---------LALDRQQIEQTRASLRTAELPALVYGGLKLTQPGVDAGQAPRLDRRLG-LL 689
RRSL_01754 EAP72360         NW-----PLLEGNLSLIDQTRENLRRVVRGMPARERAYAEIKA-RASTRYAPMTVARIVG 737
GSU3166 AAR36557            ---------PDLAREAAQLQGWLRRSLALKTDSLSWLAPWVDRHGGLSPVTLAEFWGGSV 668
ECA3432 YP_051522           YAAAI--STFTPFNQPITLAQQELSKLPMYQRVYQSLVMKATQ-VLPPDLAIRDEVGPTF 698
VC0395_0016 WP_00203        DKGAE--QVMRPYDKVIARAQVELGSMPNDQRVYRNLKLSAQT-VLGPSVNLRSLIGPVF 528
APECO1_1770 WP_00047        DSDAV--SRWTPYDKPIINAQQELSKLPIYQRVYQTLRTKALS-VLPADLNLRDQVGPTF 699

AHA_1845 YP_856379          DTVFAL---RSDNAGQVPRLLTWPGFNDFFLKQDKALIDLTAMDAWVLGQRKLSQLSEAD 751
BB0816 NP_887365            SAVFVR-KSGKPLAQGIAALYTYRGYWDVFNPRVTRVAERLRSDDAWVLDIAPPGVLDEA 763
BURPS1106A_A2840 YP_        SLVFRR-QSGKSLADGVPGLYTRSGYRNVFAPRLPGAIDSYGREEVWVLNLGASEIPNPA 771
BUBURPS1106A_A0140 Y       AGIFRL-ADGSRFQRGMPGLYTYDGYHQVFSARLPEFLARAQSDDAWVMGNAGPAARWGD 832
BURPS1106A_A0236 YP_        GTVFTR-ASGAPLARGVPGLFTFDGYRRVFDKRLAEFVRTAREDDAWVMGRAYLGDAQKK 876
BURPS1106A_A0717 YP_        SMSLRMRSNVPSTDTAVSGWFTRAGYTDVFLPRLQKSARAMLEEESWVLRDETLSGNSFQ 730
BURPS1106A_3650 YP_0        EGNTG----LVAGSYAIPGTFTRDAWFQYVQPAIRDAATKELQA-KDWVLNTASADDLTL 793
Daro_2188 YP_285397         PDNAA----LVAGSHVVSGAFTVEAWREYIQNAIKEAATNEQNN-ADWVLQTSTKDDLTL 766
c3398 NP_755273             DTLTES---LFSTEQTVPGMFTRQAWEGQVREAIEQVVTARREE-IDWVLS-DRQQDTSA 646
ECs0218 NP_308245          DQVFTS---ADDNKLVVPQFLTRYGLQSYFVKQRDELVELTAMDSWVLNLTRSVKYSDAD 615
HH0252 NP_859783            YMIFEH----TDKLSQIDKTYTKDGLMEFLSNLDTNIQTAIKIESWAYNEKMQYQALTEQ 745
mlr2349 NP_103715           AKVFAR-RSDKTLRVGVPGPYTYAGFHDAILDRVEDVAGQAALDRAVFAGGCS--ENSET 737
MXAN_4804 YP_632965         QMSATS---------RVRGAFTRKAYDDVIRER----LDLAFQDQQSWVLDRDEKVDTVS 743
Pden_2453 YP_916236         GQVLTR-LSGQTLRQGIPGAFTWQGFHGTVLPMLPGMAEAALNDRKVFAGGCA--ESADA 734
```

```
Pcar_2811 YP_0067185          RSVLAS-------SFTMPFMFTSKGWHNYVQPEIDNLVHIACQGDWVTGYLPDSLSDASG 750
plu2288 NP_929545            MLVRGK---KNWQALILAAGQYDSPYITFLTQLERDLSSIEQADRQPWLQTQQRLWQLHG 730
plu0359 NP_927714            DRLFMA---NNDKQLIIPQFLTINGLNNYFVKQDDQLIDLTVMDSWVLNLSHNVQYSDTD 761
plu3247 NP_930474            DTDAAR---LFTTSHVIPGMFTRQAWEGGIQKAIAEAVASRKEA-IDWVLS-DNRQAVSA 651
PFL_6081 YP_263139           ALVFTR-KSGKPLSEPLSGLFTAKGYRQGFLLASLNQAGTLA-EEQWVLGR---DQADQQ 728
PP_3091 NP_745235            QPGRA----LLYGTAGVPAIYTRQGWDTFVKPELIKLVSGNLRNESDWVLDGEGGDALVQ 813
PSPPH_0125 YP_272435         MPGRQ----LLYGSEAVPAIFTRQGWEEFVKPELIKLVSGNLRNESDWVLDGEGGDSVVQ 849
Rmet_0633 YP_582788          GTDARG---LLRSAAAVPGVFTRQAYEGYVADAIDAAATRRDVA-ADWVLTGSQVAAVTA 625
PA2361 NP_251051           QQQFRS-EWIAPPEAAGDGLLVKQGATLKLADDLLGLKRAIDDLKTKDFVSVALADKTLA 734
PA1669 NP_250360           GLFVGT-------DYVIPGFYTQQGYQQYFVVQGASLVNDILRDNWVLGEGSGISDMDLR 730
PA0077 NP_248767           PLVFAR-KSGKPLTDPLSGFFTYRGYREVFLTASLSQAGTIA-EEQWVLGR---DLNDAG 658
Atu4332 NP_356323          ERAFER-RSKAPMAEGIEGLFTADGYRRVVIPQVAHAARVALSEGWVRGSDDA--IKG-A 729
EvpO ABW69087            RPDKLP---EVDAAYTLEGQQRIRAFLVEMRSSMPNDIAFDKKRAAFWHEYAQKRLQAWQ 714
pRL120464 YP_764970        EQAFER-TSKAPLNEGIPGLFSATGYRTVVLPGITDAAREALDEQWVR-RDPN--PAG-V 730
STM0285 NP_459283          GTVFVR-SNGAPLDRGVPGIFTREGYRELFDKRLPEFVAAATANDGWVMGRESTPKKLTD 836
Acid_0223 YP_821521          QVVTQP-------HVVPGAFSKGGWT-FMKDAIAHADRYFNGEQWVLGDQVAGNIDRAA 707
YPK_0399 YP_00171915        DDIFVS---GNDRLLVIPQFLTRSGLQSYFIKQNDQLVDLTVMDSWVLNLTKNVEYSEAD 757
YPK_1485 YP_00172023        TQVFTL-SDDELLHEGIPGLYTYAGYHEVVKKKLTPLLMLLQEEDRWVMAQSLSSSLDPL 828
YPK_3550 YP_00172227        TLMLRRKSPSTVTDLAIPAFYTRASYHDVVNPQLQEASEAMINEEAWVLSDGKASVSQVS 727
YPK_2591 YP_00172132        DTDAQR---LFQTSESVPGMFTRKAWDEQIQQAIDKTVASRREE-IDWVLS-DNRRAISE 668
XOO3038 AAW76292           SDVFER-RSGLPLSAPLPLFTRQAFQAQIGGGIAHGVQFLADDWVLGTAP----LDPL 744
RRSL_01754 EAP72360        ETGSN----VIAGSYAVPGTFTKEAWQGYVQGAIREAASKELQT-KDWVLNVAARDDLTL 792
GSU3166 AAR36557           PAPDEP---------TITPSFT--GKGKDRIDGLMRELETAVGDSRLLAKARAG------ 711
ECA3432 YP_051522          DPVFSL---RNDKAGSVPRLLTYPGFSDYYLKQDKALLELTALDAWVLGQRERAQFSEAD 755
VC0395_0016 WP_00203       DVVFEERV-LNSSSLFIPQMLTKRGFDDYFMPQSESVSELALIDSWVLGQSKTAQFSEAD 587
APECO1_1770 WP_00047       DNVFVA---GNDEKLVIPQFLTRYGLQSYFVKQREGLVELTALDSWVLNLTQSVAYSEAD 756

AHA_1845 YP_856379          RKE-----------ITRQVNDRYVTDYVNQWQKLLTNLDVQT--------LESPEQALDV 792
BB0816 NP_887365           ARQQ----------LIVDIKRLYLNDYVARWDGYLDDLALAP--------RSSLLQSIQL 805
BURPS1106A_A2840 YP_    DAAA-----------WARDIRQLYLNDYIKTWDDYLADIRLQR--------TSTLAQSIQV 813
BUBURPS1106A_A0140 Y  AIRNTQAVAGRSS-LADDIRRQYLTDYGNYWQQYLADIRPASSGED--GAGGTLAFDLAT 889
BURPS1106A_A0236 YP_   TAEIANALAGADDPLTDAIRRQYLIEYAQQWDAFLGDIRTIG--------GTSLAFDLVV 928
BURPS1106A_A0717 YP_   IDG-------LVQKLADSARNQFLQDYISAWQNFLNDVTVRG--------VTGLDDASQL 775
BURPS1106A_3650 YP_0  EGSP--------EQIQKTLVAMYKTEYAQHWQKFMQGIAVQG--------FGNFTQAVDA 837
Daro_2188 YP_285397          EGSP--------EQIQKSLVVMYKRDYTEEWKQFVQGVSVSS--------FETFPEAVAA 810
c3398 NP_755273            DISP--------DTLRNRLTSRYFTDFAGSWLAFLNSIHWKK--------EDSLSGILDQ 690
ECs0218 NP_308245          RAE-----------IQRQLTEQYISDYTATWRAGMDNLNIRN--------FESIGQLTGA 656
HH0252 NP_859783           AQRD----------LYTHIISIYLNDYKQRWEELLQAISPKQ---------YTDKNNILT 786
mlr2349 NP_103715          SVSA----------LSQDILKLYYDDYIAQWDSFLRDMRLAP--------LTDLNIASEN 779
MXAN_4804 YP_632965        SRRE---------------LRTRYFEAYTQEWKDFLGAIRVQAP-------ENLTQMESL 781
Pden_2453 YP_916236        SAAS----------LQADMMKLYQDDFINAWEGFLRDVRLAP--------IPDLATATAN 776
Pcar_2811 YP_0067185       TKTDTSANNPLAVQLETEIRALYFADYVDHWFSLLAEIQLPP--------FHSLDDAAKK 802
plu2288 NP_929545          YVQK-------------SGMMQRVSLGNFSVRQRIMSWLGISP--------QISVRLDDTA 770
plu0359 NP_927714          RQE-----------IQRQITEQYLGDYTATWRAAMNNLDIRD--------FTDIPQALSA 802
plu3247 NP_930474          SVSP--------EELKARLTARYFTDFAGVWQDFLNSLRWNK--------TRNLSDVIDQ 695
PFL_6081 YP_263139         NVAS----------LAADVRRLYFQDYLRQWDALLADIDFVP--------ITSVAQAADV 770
PP_3091 NP_745235          KAN-----------FVREFMTRYKRDYTQAWYKMVSSVGVRH--------FADLASATRE 854
PSPPH_0125 YP_272435       KAN-----------FVREFMARYKRDYTQVWYTMIDSVGVRR--------FSDMANATQQ 890
Rmet_0633 YP_582788        TKSA--------DELRAALTEQYFAEYAAHWQDFMNSLQWES--------AATLPAAIDQ 669
PA2361 NP_251051          DHSLLS-----IDDIGVTQALSFFNDFTGYYESTLPSLNPRYR-------YSVLHAASA 782
PA1669 NP_250360          RLMV-------------ELEQLYFRDYANFWSEAVGRLGLLP--------FNDAGEGADQ 769
PA0077 NP_248767          DAAN----------LALDVRRLYFQDYLRQWDDLLADLTVVP--------ITNVTQAADV 700
Atu4332 NP_356323         TVEQ----------VAQAALQIYFDRFEKIWADTLSDLRVKP--------SQSLGDAVET 771
EvpO ABW69087           DFAG-----------QFSQGERILAGQGEWQSTIDRLPHDD--------NPYLRLARRL 754
pRL120464 YP_764970       TVDT----------IAQATLQLYFDAFEQRWSMILTDIRVKP--------SQTIGDAAET 772
STM0285 NP_459283         SLR--SQIPGQEQSVAREVRRLYLTEYARRWQDFLDSIHSINSAGE--EGSSGLAYDLQV 892
Acid_0223 YP_821521         LAQG---------------LHDRYYSDFVKEWRTYIKSANVVR--------YAGLKDATTK 745
YPK_0399 YP_00171915       RKE-----------IHRQVTEQYLGDYTATWRAAMNNLSVSD--------FEGLPQAISA 798
YPK_1485 YP_00172023       TLR------------QDLVTMYKQLQGYAAYWKRFLSSVRLISVDADSAEYAQGLSLDIAL 875
YPK_3550 YP_00172227       LLA---------AAQKLSDEARKYYLMEYANQWDAFFNDIRVRP--------INGLDDAALL 772
YPK_2591 YP_00172132       DISP--------EALKKRLTERYFTDFAGSWLSFLNSLHWNE--------AHNLSDVIDQ 712
XOO3038 AAW76292          ARSQ----------LEREVLALYQRDYIAAWDSLLADLVLRP--------VVQTGQASAV 786
RRSL_01754 EAP72360       EGSP--------EQIQKTLVTMYKTEYAREWQRFMQGVAVQD--------FSGFDQAVAG 836
GSU3166 AAR36557          -----------------FVPWYRALCLQAWQGFAASFPRGAER-------LRGSGEWQQA 747
ECA3432 YP_051522         RRE-----------ILRQVNDRYITDYINQWQKVLANIDVQT--------LDTPEQALDI 796
VC0395_0016 WP_00203      KQA-----------LREKIRDLYVADYTNTWRAALNEIDVKY--------FNDINDAVMV 628
APECO1_1770 WP_00047      REE-----------IQRHITEQYISDYTATWRAGMDNLNVRD--------YEAMSALTDA 797

AHA_1845 YP_856379          LAAITG-NDQPFQRVLASLDDNTRIRKISDVEGDPAQA-------------------- 829
BB0816 NP_887365           ARTLSA-PDSPLVRLTQGVARETTLLRDAG-----GDERSLADQARDRVSSTREALE--- 856
BURPS1106A_A2840 YP_    ARTLSS-ADSPLTRLMVALARDTPLG-DAP-----GGARNLASRAQDKVDEARNSLA--- 863
BUBURPS1106A_A0140 Y  LRALAA-PDSPLVRLARAVVRETSLSVVDA---REDAS--LTDAALSSLGRRSGAAKEVA 943
BURPS1106A_A0236 YP_   LRSFAA-PDSPLERLARAAVHETTLAQPEA---SADRS--FLQKASAQLSQQ------- 974
BURPS1106A_A0717 YP_   AAAMME-AQSPLANLLRFAARETTLTGASD---EGNIDS-WIDRQKYRFEKGRRQ----- 825
BURPS1106A_3650 YP_0  MNKLGDPQDSPIRKVLETAYDQTSWDNPSLA--NATIKKAQTGVVNWVKQWFTRQPGGQI 895
Daro_2188 YP_285397          MDRLGDAQMSPVGTLIKVVFDQTAWDNPAMA--NLGLERAQKGFIEWFKRSVLQMAPSRV 868
c3398 NP_755273            LTLMADARQSPLIALTDTLAWQA----------ATGRENRGLSDSLAKSAQELFNGKEKT 740
ECs0218 NP_308245          LEQVIS-GDQPLQRALTVLRDNTQPGVFSEKLSAKERDEALAEPDY------------- 701
HH0252 NP_859783           QLQILSESTNPLNSLIKIVNDNTHLNDTLLLNYAYGLGLPSSDIKAQFTYISN------- 839
```

```
mlr2349 NP_103715          LKDLSS-ADSALKRLLTAVVQETDLTRSDD--APADDKSGAAAKSGSKLLSKLGKLG---  833
MXAN_4804 YP_632965        LTNLTRGKPKPYGRLFRALTYNVVLDKRDAK--AAEADTGFLAKAGKLFGSEPGKDVPQK  839
Pden_2453 YP_916236        LKDLAS-PDSTLKRLLNAVVAETDLARPPEQGDSPEINPGVLKKVAGKLG--LGAAA---  830
Pcar_2811 YP_0067185       MLLLAR-NDGPIAELLQVVSRNINIGQAKG---SLASDGSVQSAS----------------  843
plu2288 NP_929545          LTRYRAYRQAVQLNSQRELLSDMETEVLVKN--ALNNEKGELDDSG--------------  814
plu0359 NP_927714          LEQVIS-GEQPISRALQILSDNTRLPEINETLPAKARQPLRDTPDY--------------  847
plu3247 NP_930474          LTLMADGRQSPLIALMNTLAYQG----------ETGQQGPSLSDSLVKSAKTLLN--KKT  743
PFL_6081 YP_263139         LRILSG-PTSPLKKLLVAVAKETDLQ---------QEERLLAAQGQKVEGG-VDQLK---  816
PP_3091 NP_745235          LGLLSDVQNSPVKNLLQAVNDNTQWDLPVKQ--AIPAPGTSRDDGFWSKVTGLFDANDTL  912
PSPPH_0125 YP_272435       LSLLSDVRNSPVKLLLSAVNDNTQWDVPAPR--EAQQTGVKRDDGFWGKVSGIFDNKEAA  948
Rmet_0633 YP_582788        LKLLADARQSPAIALMKSLAYQG----------GAGVQKTSLSDTLVNKAKDILG---KK  716
PA2361 NP_251051           ATEAMWQSLGPRSRSLASRNASRFDVQVKQVQVLQAALLELQDLQAGARLLLSLN-----  837
PA1669 NP_250360           VSGLLA-ANSPILQLLEVRENTRFPVLAESAEALAEASDKAAEKGGKLGKAAAAAAG--  826
PA0077 NP_248767           LRILSG-PTSPFRKLLEAVARETDLQ---------KGDRLVAAQVKKAADGTVDKLK---  747
Atu4332 NP_356323          TRALAN-ERNIVVEAARSIAEATDLRPGAN--PAALASAAEGDATAAVLAATVNAAD---  825
EvpO ABW69087             LTEFAEMPDDLRPGWLRVLPQWLEIRQRAHQQALVRVPGLVSDVGGMILRGTAQKG----  810
pRL120464 YP_764970        TRILAG-KPGPVETITKSIVAATDLRAPGA--EIASAAGDSLPASTTALASAANAPD---  826
STM0285 NP_459283          LRTLAS-PDSPLMRLGKAVVEQTTLVPPPD---PQARQKQLAQRASGNAGKVVQT-----  943
Acid_0223 YP_821521        LAQLAG-NQSPLLELFALASTNTAVDDP--------------------------------  772
YPK_0399 YP_00171915       IEQVIS-GEQPFRRALQTLSDNTRLPVISDLIPAKEQQELLQKPDY--------------  843
YPK_1485 YP_00172023       LRTLVS-ENSPLLNLARQAVKETTLAEKEK---AITDEINIRTQGQLLSQAK--------  923
YPK_3550 YP_00172227       ARQLSD-PSSPLANLVRFSTREITLTTDNNG--KGAMSSGWFNIQRNKLTTARRN-----  824
YPK_2591 YP_00172132       LTLMSDVRQSPLIALMNTLAWQG----------QTGQQNQALSDSLVKSAKALMN--KDQ  760
XOO3038 AAW76292           AAKLGG-PASPLKALLVLLREHTHALGRTP---AAKAGAGAAAAADTAAAAEPDVAA---  839
RRSL_01754 EAP72360       MNLLGDPTNSPIRKVLDTAYDQTSWDNPSMF--NAGLKQAKSGMLGWFKQLFSRQAPSQI  894
GSU3166 AAR36557           ATRMAT-EHGPYFAFITRMTQELHTLAGTEGLPPFAAQLFAFQIARTGGAVSHDAAAN--  804
ECA3432 YP_051522          LTDITG-NDQPFQRVLTTVSDNTRIRKLSDDDNDTAQG----------------------  833
VC0395_0016 WP_00203       LENITS-NLEPMQRLLRTLDDNTQLYSALPK-DESALKELLKSPKY--------------  672
APECO1_1770 WP_00047       LEQIIS-GDQPFQRALTALRDNTHALTLSGKLDDKAREAAINEMDY--------------  842

AHA_1845 YP_856379         ---------------ISARIGRPFMATNGVLAGRGEQG-------PLIQEVNQKLVELQH  867
BB0816 NP_887365           -----QMFGPTGPGAATRADAGD-GKLERVVDQHFEPWRRLAR--ADGQGGAPIAATTSL  908
BURPS1106A_A2840 YP_     -----QIFAGQPGGEAGAAAAPP-ASPEQIVDSHFAGLRAFAP--GGGDQAASFDAVLKA  915
BUBURPS1106A_A0140 Y      DGAQKLAARRPEQRLEKELVDNRFAALREVVTGQADTGSGPAMTDVPVAAGGRALQLDAI  1003
BURPS1106A_A0236 YP_     --ADKALGVRAQERVERELVDNRFAGLREMVTGRADT-----QTDARPGAPAGKSGLDAV  1027
BURPS1106A_A0717 YP_     VVGELSGQHYRTVLLPEHVVEEHFQAIRQLAAQLNRNN---------------TIANNPL  870
BURPS1106A_3650 YP_0     AANVDINGNP--ADVPMGPIGQEFVGLGRIVATHD--------GTSMLKGYMDTLSKVRT  945
Daro_2188 YP_285397        QMDVNVSAKP--AEIPMGPIGKEFAGFGRLVIPRDK-------ADPLVDTYLKQLSKVRT  919
c3398 NP_755273            PQQSREGDDV-----PVGPLDKTFTPLLRLLGDKAGGG----DSQLSLQTYLTRVTRVRL  791
ECs0218  NP_308245         --------------QLLTRLGHEFAPENITLAVQKDKE-------STMQAVYQQLTELHR  740
HH0252 NP_859783           -----SFKDYYDFIGEKSLLLSQIDSFNDIKKQSSTEEIK--------EILAKDIQNMSG  886
mlr2349 NP_103715          -----KVVTSGAKLLPRAGSANQVDMTGSLVADHFKPLKGTIAQVDGQPPALDAAVVALT  888
MXAN_4804 YP_632965        RELLNANSPSGAQEVTARDLEREFASLIRFATETSKTED-----GEESLTALDSYEDQLA  894
Pden_2453 YP_916236        -----RIGTKVAAAARRPGEP-EPPPPGAEIARHFAPLKAIVAEVDGKPPLIADAETALG  884
Pcar_2811 YP_0067185       ---------------LVQELENPLHDLRKLTDPGDKMN-----TSLLINQYLLALTAAQA  883
plu2288 NP_929545          ----------------LRALFNRFTLWHHETESKQSAG-----VNETLWRLYQGDAHLVL  853
plu0359 NP_927714          --------------RLRVRIHREFAPETAVLEESGDKN-------STLQGVYQQLAALHR  886
plu3247 NP_930474          PRMINQQTAG-----LHGPMDETFGPLLALMGENQAEGRIKADSTLNLQTFLTRVTDVRL  798
PFL_6081 YP_263139         -----QRLGNLLGQEQ-SANPAAVASSEDPVSAHFAELNSLVSKGEG--EPAAIDGLLAD  868
PP_3091 NP_745235          PANVAPALPA----VDDGSLAKRFEPVARVFAENNAEGA----DSTIMDRYLAALRKLKV  964
PSPPH_0125 YP_272435       ASVLISPLPA----VDDGSLAKRFEPVSRVFAAQNAEGA----DSTIMDRYLAALRKLKV  1000
Rmet_0633 YP_582788        DDAPNSAKAA-----PAGPLDAAFGPVLRLAGQPGQAGQ-GGNNDLSLQRYLDRITALRL  770
PA2361 NP_251051           ------ALAVADIGQALRDIDEQAVLREPVDFSRWSGAPNFGLQMFRAQDSAELKQSLNS  891
PA1669 NP_250360           --KARDALAKNLPDTAKKSLQRRFEPLHRLLDDNNGP-------AADLTPLLQALNELQL  877
PA0077 NP_248767           -----QRLGSLVGQEEEGAREQPRQVDSDPISAHFAELNSLVSKGEGGNEPAPIDSLLED  802
Atu4332 NP_356323          -----PYARLRDMLATKGAATTGEQPN---------------GDKTGGSPSEQLLAHFKL  865
EvpO ABW69087             -----------ALDQTRSQLVGRLDAIRLYQEYQAAMTQAMNSVVISQAQATKVAADFHG  859
pRL120464 YP_764970        -----PFGRLRDALKAPAGQSSPDDQP---------------KDAPR-SEIGQLEPILQR  865
STM0285 NP_459283          --AKLFQDIHPEERLEKTLVDDRFAALREVIAGRTDGG----------QSGGGTMQIASL  991
Acid_0223 YP_821521        ------------------AVAKVFQAVQAVVPPGSTDRFIAPSNQNYMNALVAIQTSLEA  814
YPK_0399 YP_00171915       --------------LLLTRINREFSPETAVLVENGDKG-------SVIQSVYQKLTELHR  882
YPK_1485 YP_00172023       --KLQETIAFRENKLIKEWVDNRFESLRVFVNGEQSADAP----GGNNLSVLQGTQMSRI  977
YPK_3550 YP_00172227       IIDEMSGERSRFRLTPEKAVEDRFELLRRLGYALQSAS---------------TSNSDPL  869
YPK_2591 YP_00172132       APAIDQSAGG-----PVGPLDETFGPLLALMGKGDAQNRLSSDSSLSLQTLLTRVTRVRL  815
XOO3038 AAW76292           -----EPIERHFEPLTRLLDGGP---------------------------------GASA  861
RRSL_01754 EAP72360       NVNVDVGGNADAGTLPMGPVGKEFAGLARVVVVRD--------DKSLLRGYMDALSKVRT  946
GSU3166 AAR36557           ---------------VTAESRRLMASIRRRLGNEAAAS--------TIDPTFMASRSWQE  841
ECA3432 YP_051522          ---------------INTRTGRPFMTINAALSGRGEQG-------PLVQEVNQKLTDLYH  871
VC0395_0016 WP_00203       --------------KVASMIETPFADLNGMLKPVGSKP-------AYMTEVLASVDELKS  711
APECO1_1770 WP_00047       --------------RLLSRLGHEFAPENSALEEQKDKA-------STLQAVYQQLTELHR  881

AHA_1845 YP_856379         YLELIVNATEPGQSALKAVQLRMTNKYADPVFA----------LQQYARSLPAPLDRWVG  917
BB0816 NP_887365           VNELYTYLTAAD----AALRSASPAPASDVVTK----------LRAEAGRLPRPLRDVLD  954
BURPS1106A_A2840 YP_     IDALYTYLTATD----DALRSGAAPPPSDAPAR----------LRAQAGRLPTPVREVLD  961
BUBURPS1106A_A0140 Y      LTLINEQYTRLVVADNALSS-QSMPPALDIGTT----------LQMEAEKLPAPLRTVLG  1052
BURPS1106A_A0236 YP_     ANLLNDYYTALTVSDDALAN-NSMPPANDTAAK----------LKMAAQTMPAPFRSVLT  1076
BURPS1106A_A0717 YP_     SRLFEPLYRQLGLVNGALQAGQVLPAQYDAFSR----------LKETAARQPEPVRGIML  920
BURPS1106A_3650 YP_0     RFNVIKNQGDPGPGARQLMQQTLDGNGSELADSLKFVDEQ--MLTGLTDSQRKSLRPLLV  1003
Daro_2188 YP_285397        RLNQLKNQGDPGPGALKLMRETIDGNGSELAETLRFVDEQ--MLTGLPDNQRAVLRPLLV  977
```

```
c3398 NP_755273            KLQQVTNAPDPQEMTQQLAQTVLQGKTVDLTDTRDYG-RL--IAASLGEEWSGFGQALFV 848
ECs0218  NP_308245         YLLAIQNAPVPGKSALKAVQLRLDQNSSDPIFA----------TRQMAKTLPAPLNRWVG 790
HH0252 NP_859783           KITEFTKDDTKNIKDKLIYILEGSNDETDAFVE----------LKRNTAMLPHALKQYYN 936
mlr2349 NP_103715          ALSNVLQTVTANPNPQDAIKKQGG--LAELTGA----------VARQAQILPSPINEWLG 936
MXAN_4804 YP_632965        TVQTTLLAVKDKPAESGLLLDKIESTRNNVEMM-----------VRKQTDNVAIFERLLL 943
Pden_2453 YP_916236        ALSKELLTVQASPDPQAALLSRGG--LPQLTGD----------LRNVAATLPDPVNAWLG 932
Pcar_2811 YP_0067185       EIEQMAAAVDVPQQSFQYASTILGSNGVTSSELYKCWLSTNSLLNGTDVRTRKVAEHLLY 943
plu2288 NP_929545          RYAFLSAASRLQTQWESKVIWPMEKLSRQELFD----------GRELNARLYEYSNAFVR 903
plu0359 NP_927714          YLLGIQNAPVPGKAALNAVQQRLEQHNDDPIFD----------VQQRAKTLPAPLNRWVG 936
plu3247 NP_930474          KLQQMNNSDDPPAMANALAKTVFEGKSTDLTDTQGYG-RL--IAASLGGQWRSFGQTLFV 855
PFL_6081 YP_263139         MNALYVQVSAMVGASGDALLGEAKNQASAAAAR----------VSLTAERQPPVVQGLVK 918
PP_3091 NP_745235          RMNNIQRSQDVGKSSKQLISETLEGQPSEITTVRNYVESS--VDTSQ-DGLSRSLQGLFS 1021
PSPPH_0125 YP_272435       RMNNIQRSQDVGKSSKQLISETLEGQPSEVTNVRNYVETT--VDTSQ-GGLSTSLQSLFS 1057
Rmet_0633 YP_582788        RLQQVSIGADADDQARQMAQAMFQGKSSDLADTQAYA-QL--VAASLGAEWAGMGETLFV 827
PA2361 NP_251051           QFNAMAAVAERHAPALEWLQAQQSSLATADYNAFVRFSALNAELQKFKADNPASTAAQLA 951
PA1669 NP_250360           QLAGLARASQPEQAAFDMAKSRMGGQRDALSNLR-----------NASARLPRPLGGWFS 926
PA0077 NP_248767           MNALYVQVSAMAGASGDSLLGDAKNQVAAAASR----------VALSAERQPPVVQGLVK 852
Atu4332 NP_356323          LNEQLARSATTSDEVAKVFDVDSQ--LTKANQD----------LLQQARELPAPLDVWVA 913
EvpO ABW69087            FDSQQSGQSSLQTAYARLMSLRSLLGYNRPDEQLTWGLMAG-PLQLVIRYADQQASCFLQ 918
pRL120464 YP_764970        VQEQLSRATTSTAEVAKVFDVDSQ--LTDANQD----------LLQQARKLPAPVDTWMA 913
STM0285 NP_459283          LTMLNEYYTQLTIADSALAA-GTLPARITAADK----------LQLEAAKLPAPLKNILL 1040
Acid_0223 YP_821521        IANQPGAPNDAAAAQTVTNATQAKVNTRQMAQAFT---------IDAEGHIEASVQKLLE 865
YPK_0399 YP_00171915       YLLAIQNSPAPGKAALKAVQLRLDQNNSDPIFE----------VQQLAKNLPEPLNRWVG 932
YPK_1485 YP_00172023       IGTLNDQYTRFVVYNSAFEY-GDIPPLSQDGER----------IAVESQTWPDPFRNIIA 1026
YPK_3550 YP_00172227       GRTFEQIYNQLITLSTSLRAGQILP-QNNDFSR----------LQIDMARQPEPVRSIMM 918
YPK_2591 YP_00172132       KLQQVVNASDPQEMTQVLAQTVFQGKSVDLTDTQEYG-SL--IAASLGEEWSSFGQTMFV 872
XOO3038 AAW76292         LDETLAALTQMSRTLLGAAAGVAADQNAPALLI----------AAQQAEQLPPPLGNWMA 911
RRSL_01754 EAP72360        RFNLIKNQGDPGPGARQLMQQTLDGNGSELADTLKYVDEQ--MLTGMTDGERQTLRPLLV 1004
GSU3166 AAR36557           YQAALAAIAPAASSRQQAFQLASQTFTDDPATG----------KTPFHAAWNAAARLKRD 891
ECA3432 YP_051522          YLDQIVNATDPGQAALKAVQARQGNKFADPVFA----------LQQYARSLPAPLDRWVG 921
VC0395_0016 WP_00203       YLKSIQDAPDVGMAALDATKARVKLVNADPIYT----------LKRISSGLPKPLDSMMA 761
APECO1_1770 WP_00047       YLLAIQNSPVPGKSALKAVQLRLDQNSSDPIFA----------TRQMAKTLPAPLNRWVG 931

AHA_1845 YP_856379          QLSEQ--------SSRLVIDLAMSSLNQEWQDKVLTPFNSQLAGRYPFDPSSNKDVPLSE 969
BB0816 NP_887365           DLSTG--------AAGEVSGVARERLGEDLYATIGLFCRQAIAGRYPFAPGSPRDVAPND 1006
BURPS1106A_A2840 YP_    DLSNV--------ANGSIASVEQRNVAQRAGANVGDFCRQAIAGRYPFARGAARDVAPSD 1013
BUBURPS1106A_A0140 Y    GIATQ--------AADKVGREVGSLLAMQVDSSVGNACRAAVDGKYPFAR-SAQEVDIED 1103
BURPS1106A_A0236 YP_    GLAAQ--------GSREVNRGIGQLLSRQLQATVGDVCRLAIEGNYPFAPDSKRDVGIED 1128
BURPS1106A_A0717 YP_    DLVSS--------GSTMTTRESGALLNRGAAGATKMVCDQGFTGRYPMRRGAQADAGVED 972
BURPS1106A_3650 YP_0    RPLMQ--------AFAVVIQPASVEVNKVWNAQVYQPFQNSLANKYPFAAGAKVEAGAGE 1055
Daro_2188 YP_285397        RPLLQ--------SYAAIIRPASEELNKTWEAQVYEPFNRKLAIKYPFAAKSNIEASPTE 1029
c3398 NP_755273            RPVEQ--------SWRQVLTPAADSLNRQWQRAIVSHWNQDFAGRYPFKA-SQNDASLPL 899
ECs0218  NP_308245         RLTDQ--------AWHVVMVEAVHYMEVDWRDSVVKPFNEQLANNYPFNPRSAQDASLDA 842
HH0252 NP_859783           EISTL--------SWNVIEKSTYALLNNAWKKEVYDTFINDISPFYPFNAYSDKSLPLNT 988
mlr2349 NP_103715          GIAGD--------TSGLSQKAVTNELNAIWRADILPFCQAALNNRYPFSPDSAVDVNVRD 988
MXAN_4804 YP_632965        PPFQE--------MRSVVFVGVACNKSKLWQDQVVMAWSSAFKGKYPFDRASQADAPLPE 995
Pden_2453 YP_916236        AIAGD--------TISVTREAILAQLNARWRADVLPFCRAATGGRYPFVADSAIDVTVSD 984
Pcar_2811 YP_0067185       KPIAG--------AWKAIIQQTQSYVQNQWSSTVFLNYQGKIAGKFPFSN-NGADASLND 994
plu2288 NP_929545          DTAGYAL----DIRPEGIKRQEIEDVVFPFSDDFIFYINNLIQPNDLLSATSDIRRRLQE 959
plu0359 NP_927714          ALAEQ--------AWRVVMREAIRSLEIEWHDTVVRQYQTYLAGRYPFNPDATEEVPLSE 988
plu3247 NP_930474          EPLAQ--------AWQGVLKPATANLNSQWQTAIVDNWDRAFAGRYPFAG-GDSEISLPM 906
PFL_6081 YP_263139         SVVGS--------TTNTMMGGVRNQLNAAWVSEVVNVYRQSLSGRYPMSPGSSRDATLED 970
PP_3091 NP_745235          LPIQY--------AWATLRDPAGEQIAKAWAQQIAKPWEQVMAHRYPIAGSSRNEASVKD 1073
PSPPH_0125 YP_272435       LPIQF--------AWETLRDPAGEQIAKAWAKQVAKPWEQVMAHRYPIAADSRNEASVKD 1109
Rmet_0633 YP_582788        RPVAQ--------ATQAIVLPAQASLNDVWQRFIMAEWNRIFAGRYPFAN-TTNDASLPE 878
PA2361 NP_251051           KLVGNDFNQMDIGSCADILNGVLLPSSRSDLATRLVDLRQGALGRCQSLQQQAAQAWKD 1011
PA1669 NP_250360           VLAED--------AWRLVLGDAYLYLNQRYQSELYGFYSKAINKRYPFNAHSASDVAVSD 978
PA0077 NP_248767           NVVNS--------TTSSMMGSVRNQLNAAWISDVVSVYRQSLAGRYPIAAGSSRDATLED 904
Atu4332 NP_356323          GVAAD--------VGSLAVKSARSRIAELWTADSASLCSSIVTGRYPFDRASSRDVAIAD 965
EvpO ABW69087            SAWQDRV------IAPSRWTLNQAERADKLYGDNGLLWTFLSGPIKPFIQRNDSGWQEVD 972
pRL120464 YP_764970        GLAAD--------VGSLAVKSARSRISDAWAAEGAGFCSNVVAGRYPFDRKSPRDVAMSD 965
STM0285 NP_459283          DLTKQ--------GTRKINAGTGDVLNTQMEAMMGDDCRDAIDGRYPFAD-SPQEVSAED 1091
Acid_0223 YP_821521        DPITY--------LDAMLRSIDTPEINAGGKALCGSFR--PVLSKYPFSASASSEATLAD 915
YPK_0399 YP_00171915       ELAEQ--------AWRVVMMEAIQSLEVEWNETVIKQYQTYLAGRYPFDPHAKQDVPLSE 984
YPK_1485 YP_00172023       PLLTG--------SSQKFENKVITHTSKAIDMGVGEICRNTLQGRYPFAE-SEQIVSLND 1077
YPK_3550 YP_00172227       DLLAV--------GQTQSLQQSKANLSKGVSSIASDLCSNAVSGRYPFNRLASEEIGIGD 970
YPK_2591 YP_00172132       QPLTQ--------AWETVLQPSSASLNDQWKNAVVANWKSAFDGRYPFAA-SKSDASLPM 923
XOO3038 AAW76292         TLSGR--------GRALTASSAGEALRNAQRQAAGRECALLVSGRFPFVPGSNNEIPLRD 963
RRSL_01754 EAP72360        RPLLQ--------SYAVVVRPASVEINKVWNAQVYQPFSQTLADKYPFAANAKIEASAAE 1056
GSU3166 AAR36557           IAGAG--------ADDAFWRLVTGPLDFLWGYVRREAGCQLQTLWEEQVLAPTLGMPPQQ 943
ECA3432 YP_051522          QLAGE--------SASLVTGLAMSSLNQEWLDKVVTPFNEKLADRYPFDPSSNKDVPLSE 973
VC0395_0016 WP_00203       KLADE--------SWYVVKQEAIKHLEVRWTEDVYKTFQSKLAGRYPFNPASNKDVALAD 813
APECO1_1770 WP_00047       KLADQ--------AWHVVMVEAVRYMEVDWRDNVVKPFNEQLADNYPFNPRATQDASLDS 983

AHA_1845 YP_856379          MERFFAPN-GTLDSFYQVNLKPMVESGLMEGE-------------------FSSPIQAE 1008
BB0816 NP_887365           LARLFAPN-GMMDEFFQKNLVSQIDVSEARWRFKPGM--------------DGTP-GQASA 1051
BURPS1106A_A2840 YP_    FAQLFAAG-GLMDDFFQKNLQTLVDTTAHPWRFNNRN--------------AEAD-PSAAA 1058
BUBURPS1106A_A0140 Y    FNRLFAAG-GLFDAFFQKALAPHVDTHSKPWRYKALN--------------PGMPPIRGPS 1149
BURPS1106A_A0236 YP_    FTRVFAQG-GVIDDFFAKTLAPFVDTSTRPWRYKTLP--------------GATEPVEGPD 1174
```

```
BURPS1106A_A0717 YP_   FERLFSAQ-GLMATYFRDHLAAYVDTSAKPWQALRSN-------------GGPNGMVSQS   1018
BURPS1106A_3650 YP_0   IAQVFGPD-GSIAKFVGTTLGPLAVRRGDTLSARTWG-------------DMGLALTPDF   1101
Daro_2188 YP_285397     IAQVFGPE-GAVSKYVESAMGALVVRRGNTVTPRTWG-------------DLGITLQPEF   1075
c3398 NP_755273         LAQYLR-DDGRINLFIAANLSGVLKREGRYWVADAMN-------------TQGLTVNPDF   945
ECs0218 NP_308245       FERFFKPD-GILDTFYQQNLKLFIDNDLSLEDG------------------DNNVIIRED   883
HH0252 NP_859783        FKDFFGNT-GTLQSFYTQYLSKILQKKGNTYVPNPAY--------------SETIKLSAQ   1033
mlr2349 NP_103715       FQRLFGPT-GLIDAFTTDHLINYVDTASEPWKWR-AD-------------FG----LDPA   1029
MXAN_4804 YP_632965     VAEFLRPEGGLVRKFVKEQLLEDVVATGRRWEFTSS----------------GGVMYRPD   1039
Pden_2453 YP_916236     FQQLFGPG-GRFDGFTNETLAPYVDTSARPWRWR-AD-------------LG----LDAR   1025
Pcar_2811 YP_0067185    VADFFRPEDGLIWAFTDDQLKPFLRKERNRWTEKTWL-------------GTGLAFDKSL   1041
plu2288 NP_929545       KLALLESEQQLTTAQQEPEQEQKPSAEVTIVSQPATAN----------RGAKVLPVGSSV   1009
plu0359 NP_927714       FGRFFRPG-GTLDAFYQQNLKPFVENNLTHRA-------------------DGQQLIRPD   1028
plu3247 NP_930474       LGQFIRSDSGRIEQFLTRQLGGILHKAGNQWVVDEVN-------------GQGVQINPDF   953
PFL_6081 YP_263139      FGQFFGTG-GVMDNYFRKYLQPYVNTSTTTWSWQPGA-------------AQKL-GISPG   1015
PP_3091 NP_745235       LQRFVDPDSGLLPAFKRNEIGNLAG--GEGLGVGDG--------------KGPALVNPGM   1117
PSPPH_0125 YP_272435    LQRFVDPESGLLPNFKRNEIGNLSD--GEGLGMGSGS-------------KAAPLVNPKM   1154
Rmet_0633 YP_582788     LAQFLRPQGGTIQTFLATQLAGVLELQGDQWMPAAG--------------ARGITFDPEF   924
PA2361 NP_251051        LADYFNQYLAGRFPFAYSLEAADAEPGRVRHLLQLMETRLPQVREGLAQVRSPDLPAAED   1071
PA1669 NP_250360        FREFFKAQ-GVAERFFETYMRPFVSGDPGSYRLR-SV-------------DGYSLPMSKV   1023
PA0077 NP_248767        FGHFFGAG-GVMDSYFRQYLQPYVDTSASTWRWQPGA-------------AQKL-GINPG   949
Atu4332 NP_356323       FTRLFAPT-GVFQSFFKQRMEPFVDKTTTPWSWK-GT-------------FGAA-GIPSS   1009
EvpO ABW69087          TLGQSVPFTADFLPFINRALIRQVDLKAGKIALHQKQ-------------QQATLAQQQQ   1019
pRL120464 YP_764970     FIRLFGPE-GLFKTFFKEKLEAFVDTSASPWGWK-GT-------------FGAV-SIPSA   1009
STM0285 NP_459283       FNRIFASG-GVLDAFWSKQLAPLADTASDPWRYKPTE-------------GNMT-LQGPD   1136
Acid_0223 YP_821521     INTLFRKPDGLIWTFFDQKLQKVLIRQGAQFVQN----------------PSSKVTVNPA   959
YPK_0399 YP_00171915    FERFFGPK-GTLDAFYQQNLKPFVENNLTGGS-------------------DGELLIRPD   1024
YPK_1485 YP_00172023    FERFFGTD-GTVDSYFKKNLESKVDTSSIPWRYKGS-------------------VSSEG   1117
YPK_3550 YP_00172227    FNRMFGRS-GAMQSFFDQNLAPYVDTNGNRWKVKPDS----------------NGVVTPK   1013
YPK_2591 YP_00172132    LAEFIRKDSGRIDSFLTRELGGVLHKEGTRWVPNKVN-------------SQGLTFNPAF   970
XOO3038 AAW76292        FAELFAPG-GRMDRFAQQALLGKVDTSGPGWKWNAGM-------------AAAG---ADD   1006
RRSL_01754 EAP72360     IGQIFGPE-GAIAKFANTTIGPLSVRRGDMLAARTWG-------------DLGLAFVPEF   1102
GSU3166 AAR36557        AGPMLLGADGLAWRFVKGPAAPFVRGTAAGYAPRQAL-------------GGALPLEGSL   990
ECA3432 YP_051522       MEMFFMTG-GTLDSFYQTNLKAMMESGMLEEG--------------------MASPLQAE   1012
VC0395_0016 WP_00203    FEAFFAPN-GTLDNFYNQQLKMFIDENISVASDD-----------------SAQSIIRKE   855
APECO1_1770 WP_00047    FERFFKPD-GILDNFYKNNLRLFLENDLTFGD-------------------DGRVLIRED   1023

AHA_1845 YP_856379      LVKQLDRAARIRQIFFSQQ--------GNLEVQFALEPIELTANKRR------SVLNLDG   1054
BB0816 NP_887365        LLDSFQRAAAIRDAYFMA-------GSAEPAFRVSIRPVEMDPAITQ------FVMDVDG   1098
BURPS1106A_A2840 YP_   MLGSFEKAAVIRDVYFGG-------GARTAQIKVEIVPLEMDPSISE------MLLDVDG   1105
BUBURPS1106A_A0140 Y   -LEPFERAAAIRDVFFREP------GAKRMAWRMDAKVASIDPEITE------LIVDVDG   1196
BURPS1106A_A0236 YP_   -LEPFQHAKAIREVFFGAP------GQKQMAWKADIRIPELDPTITS------LAIDVDG   1221
BURPS1106A_A0717 YP_   VLNSYETAERIRGAMLDDS------G--HLRVSTVLRFIDMDSQLSE------AQLSVAG   1064
BURPS1106A_3650 YP_0   TGGFARWVAPLAGGAATSA--AA-SSE---PQTVFQVLPQPSSGTTE------YTIAIDG   1149
Daro_2188 YP_285397     MSDFARWVSPLEGGAATDAGGGA-GGAGGQAQTVFMLRPQPSPGTSG------YVIEIDG   1128
c3398 NP_755273         IRALNRLRDVADTAFASGD-----------AGIHFELRAKPARDVMK------THLVIDG   988
ECs0218 NP_308245       IIAQLETAQKIRDIFFSKQ--------NGLGTSFAVETVSLSGNKRR------SVLNLDG   929
HH0252 NP_859783        FISFFNQISNLNSIFDNNN---------NLTLNFFIQCLGLSSDFSS------LDISYNN   1078
mlr2349 NP_103715       ALAAFEQARHIRDDLFPG-------GTG-PVMNFTLEPKDLSPNVAR------VTLNLDG   1075
MXAN_4804 YP_632965     LLGFLEKTGALSTTLFPGGDTVDPLVRFQVRLRPGVSADGMASQISS------ITLTLDG   1093
Pden_2453 YP_916236     TLAPFEQARAIRDALFPG-------GAG-PVMAFTLEPKDLTPNAAR------VTLNVDG   1071
Pcar_2811 YP_0067185    LRSLGLASRITSGMFRRGQ---------IQPDVHFSIYPIPSQGLRV------VRFETNG   1086
plu2288 NP_929545       TLSCDDGVQQLKSMNFNDSMTLHWSPGTCGKVQIDIYFPRFTLSKSYIG-----SDGLLQ   1064
plu0359 NP_927714       VLEQLKRADRIRDTFFSPQ--------NGLGVQYAIEPLSLTGNKRR------SLLNLDG   1074
plu3247 NP_930474       LKAINQLSQLSDLLFADGS-----------QGLRFELRARAVRDVVE------TDLTIDG   996
PFL_6081 YP_263139      VLQTFQRASNIRDAFFRSS------GGTQPAVRFELKPVAMDANISQ------FLLDLDG   1063
PP_3091 NP_745235       LNSIDKASSVGQVIASLSD-----------RDNGFEIMLEPSANFTD------IVFTLDG   1160
PSPPH_0125 YP_272435    VSNIDKASSLGEVIASLSD-----------RDNGFEIMLEPSAYFTD------IIFTLDG   1197
Rmet_0633 YP_582788     LKTINILQRIGAHMLVQGD-----------PQYRFELKPIATPGLTD------TKLTIDG   967
PA2361 NP_251051        FVRRLEQAQRWLGPLFERD------KSGLLGVALDIDWRSDRSLERGADQVIAWSLYSGD   1125
PA1669 NP_250360        YLDQMGTAQLIRQSFFAEN-------PAEPQVRFRLEPYTLDPGVSR------AEFRFDG   1070
PA0077 NP_248767        VLHTFQRAAAIRDAFFRS-------GGMQPTVRFELKPVTMDAAISQ------FILDLDG   996
Atu4332 NP_356323       AVAQFENADKISRAFFPN-------GSETPTVSINVKPVSLTNASSA------VMLEIEG   1056
EvpO ABW69087          KQRWQTVAAESGEKLAELNTAAAALKAKSYPVALTGLPTDVNSNAKAKVVGTLLTMQCTN   1079
pRL120464 YP_764970     AIAQFENADKINRAFFPA-------GSENPSISINVKPVSLTEAASA------VMLEIEG   1056
STM0285 NP_459283       -LTPFQQAKQIRSVFFNSE------GGKKFSWSMQISVVDMDPAIME------LVIDIDG   1183
Acid_0223 YP_821521     FLNFFNTAAAFGEALYADG--------GQDPHFSYSLKPETTEGVQM------LTVRLDG   1005
YPK_0399 YP_00171915    VLQQLAQARKIRDTFFSAQ--------NGLGTQFAIEPVLLSGNKRR------SVLNLDG   1070
YPK_1485 YP_00172023    -LAFFEQAAMIRNVLFNTN------DGKKMALDFSVSVRYLAPTITQ------LIMNFDG   1164
YPK_3550 YP_00172227    TLRSFENSAQIRDTFFDAS------G--KMAMSMIIRPISLTPSILE------AVLDVDG   1059
YPK_2591 YP_00172132    LAAINQLSQISDILFTDGS-----------QGLRFELLARPVPNVVE------TNLAIDG   1013
XOO3038 AAW76292        VLARAQLADEIKQAFFAG-------GGAQPQVAFTVS-IPPQAGIRR------LQLEVDG   1052
RRSL_01754 EAP72360     TSGFTRWIAPLTGGAAGGAGGAA-AAA---PQTVFQILPVPSQGTTE------YTIEIDG   1152
GSU3166 AAR36557        FAFLGKGAQLNAMASGRQS---NYTVAIKGLPTDANPEARIKPHGTR------LELQCAG   1041
ECA3432 YP_051522       LVKQLERATRIRQTLFNAQ--------GSLEVHFVLEPLELTANKRR------SVLNLDG   1058
VC0395_0016 WP_00203    VLDQIKQAQKIREAFFNRK--------GILDVSFSVEPLSLSNNKRR------SVLNVDG   901
APECO1_1770 WP_00047    IRQQLDTVQKIRDIFFSQQ--------NGLGAQFAVETVSLSGNKRR------SVLNLDG   1069

AHA_1845 YP_856379      QLLEYAHGR----------RTKIPLVWPNTMRDGAESKITLVPAARERSPRS--------   1096
BB0816 NP_887365        QTVRYAHGP----------QVATSVQWPG-PRGANQVRIELTP-QAGAAG----------   1136
```

```
BURPS1106A_A2840 YP_      QIVRYAHGP----------QVPTAVQWPG-TRGSNQVRLQVTE-QSGATGG---------  1144
BUBURPS1106A_A0140 Y      QSQRYVHGP----------VLPFSVNWPG-PRGGAIAEITAKPRIRPDTST---------  1236
BURPS1106A_A0236 YP_      QTALYQHGP----------VAPFAVSWPG-PRGGVHAAITASPRIRPDTSA---------  1261
BURPS1106A_A0717 YP_      QTVRFAHGV----------TSPHRVDWTN-QNTQLAIKLQLKSV-DGRMTT---------  1103
BURPS1106A_3650 YP_0      QQLRYRNTP----------PQWTNFVWPNP-QGSPGATLSATTFDGRTLQL---------  1189
Daro_2188 YP_285397       QKLAYRNGA----------PQWANFVWPNA-AGAPGAKITATTFEGKIVEV---------  1168
c3398 NP_755273           QELEYFNQK----------ERWQRFNWPDE-QWQPGASLSWTSTQAMERIL---------  1028
ECs0218  NP_308245        QLVDYSQGR----------NYTAHLVWPNNMREGNESKLTLIG-TSGNAPRS--------  970
HH0252 NP_859783          QTLHYDHTL-----------NPKIQIIIEQFNSSTELRLSVNDYYQMPQYN---------  1118
mlr2349 NP_103715         QNLVYYNNA----------TRPQPMTWPG-KDGTGVISLAFQPVDGSPEVM---------  1115
MXAN_4804 YP_632965       TDETYRNGPD---------TVWKPMIWPG-QAGKLGARITVQSADGSTETT---------  1134
Pden_2453 YP_916236       QSLVYFNAA----------ARPMPMTWPG-KDGTNLISLSFTPVDGTGEAI---------  1111
Pcar_2811 YP_0067185      QQMIYQNEP----------QEWKRFQWPG--DEDQNSAFIGCIPSGNQSMVK--------  1126
plu2288 NP_929545         FMHAFSRGE----------LTLAAQAFPQRRDELSALGINNITVRYQVSES---------  1105
plu0359 NP_927714         QLLDYAHGR----------SSIVHLIWPNAMRAGVESQLTLIPDAGGKSPRA--------  1116
plu3247 NP_930474         QKLRYFNQM----------ESWQSFRWPGE-TYKPGVMLTWTSVNAGARLF---------  1036
PFL_6081 YP_263139        QQLSYDHGP----------SRPVAMQWPN-PGSIGVVRLSIMPPSATGRSG---------  1103
PP_3091 NP_745235         QEQHYRNGR----------SSWNRFAWPGT-STAPGARLDVVTLSGTRVTV---------  1200
PSPPH_0125 YP_272435      QEQHYRNGK----------TSWSRFSWPGT-TTAPGARLDVVTLTGERITV---------  1237
Rmet_0633 YP_582788       QKLHYYNQR----------ETWNRMTWPASNLQDPGTLLQWQTEKAGTNKN---------  1008
PA2361 NP_251051          QESRFPGAQDKGLTWNVGDPLKIMLRWAKNGSQRPADDPRQASLAVADLEAG--------  1177
PA1669 NP_250360          QSMEYRHGP----------IVPMSFKWPSDADGGRTS--LVLEKMAGRPVGI--------  1110
PA0077 NP_248767          QQLTYDHGP----------SRPVAMQWPS-ANGLGVVRLTVTPPPSSGRSG---------  1036
Atu4332 NP_356323         ERVVYYHGP----------IQAKSITWPSRENTASLSRIAFQ--PGGWQQA---------  1095
EvpO ABW69087            ATLRLNNLNF---------AVMQSVLWSQQQCGQVTLQIRLPTFTLTRSYPGPQGFVHFL  1130
pRL120464 YP_764970       ERVVYFHGP----------IQSKSITWPS-TDASNVSRLAFQ--PGGWQQA---------  1094
STM0285 NP_459283         QVLRYAHGP----------DRPLKVTWPG-PRNGSMAEITASPRIRQDTST---------  1223
Acid_0223 YP_821521       QTLTYNAGTA---------AAFKKFNWQGSGTHEALLSVRFGTQDLGLASG---------  1047
YPK_0399 YP_00171915      QLLDYAHGR----------SGVVHLVWPNSMRAGVESKLTLVPDESGKSPRT--------  1112
YPK_1485 YP_00172023      NTVRYSHGP----------VTPAFFKWPG-LRQGTIVSLTAQRQKASAMPD---------  1204
YPK_3550 YP_00172227      QIINYSHGS----------NQPVKVDWPG-PKGGVYIRLTFKTQ-DGNVET---------  1098
YPK_2591 YP_00172132      QKLHYFNQM----------ESWQSFRWPGE-TYKPGTMLTWTGVSSGARLY---------  1053
XOO3038 AAW76292          QALDVRDGA----------RATQSMTWPG--PTPGLVRLAAWDASGQALPV---------  1091
RRSL_01754 EAP72360       QQLRYRNTP----------PQWANFVWPNP-QGAPGARVSATTFDGRTVEL---------  1192
GSU3166 AAR36557          QAQALANHNF---------PAGKTFYWSPDSCGDVIFQIEAGDQVLTKRYAG--------  1084
ECA3432 YP_051522         QLLEYSHGR----------RQKTPLVWPNSMRDGAESKLTLVPDDRERSPRS--------  1100
VC0395_0016 WP_00203      QFLAYSHGP----------RENVELIWPNTLRDSAVSKVTLIPTQTNMSPRS--------  943
APECO1_1770 WP_00047      QLVDYSQGR----------NYTAHLVWPNNMREGNESKLTLIG-TSGRAPRS--------  1110

AHA_1845 YP_856379        ------------------EGFVGPWAMFRLMDKGELTQVNDATFDVRFPVDQGAMTYRVY  1138
BB0816 NP_887365          ------------------LSASGPWALNRLLDQARVTRGASPEISLASFDIGGRKVVLEI  1178
BURPS1106A_A2840 YP_      ------------------FTTEGPWALHRLFDRAGVSGGRGPEQMVARFAVDGKPIVLQV  1186
BUBURPS1106A_A0140 Y      ------------------IATTGPWALFRLIERGRLTGTTSASRLMLDFDFDGRHAALEL  1278
BURPS1106A_A0236 YP_      ------------------VSTDGPWALLRLLQKGRVSGTATPGRTRVTLDFDGRKAVLDL  1303
BURPS1106A_A0717 YP_      ------------------LQFDGPWALFRFFDAGQAVGGTGTADRRERLYQTSLGTVRIE  1145
BURPS1106A_3650 YP_0      ------------------VNEPGRYGLEKLINSAQRKRRPDGTFDLTWTQGSVSVSVTMR  1231
Daro_2188 YP_285397       ------------------INVPGRFGLEKMINAAKRTRQADGSFHMTWLADGVEVGIDLR  1210
c3398 NP_755273           ------------------ADYRGSWSLIRLLEQAQVTPVDSSTFKVVWKAQDGLP-----  1065
ECs0218  NP_308245        ------------------ISFSGPWAQFRLFGAGQLTGVQDGNFTVRFSVDGGAMTYRVH  1012
HH0252 NP_859783          ------------------KIYSGEWAWLRFIK--DITPAQNTGNTLYFENNKQWYFDFSI  1158
mlr2349 NP_103715         ------------------LNETGSWAWLRMLRGGRFAATKLTDVYSLRLGTKGMWADFEL  1157
MXAN_4804 YP_632965       ------------------LEAEGDWGLFRLLERVKRIEPSADGRYFTATWEIEEMNGALV  1176
Pden_2453 YP_916236       ------------------STETGAWAWLRLVRKAGLSPTALPEVFGLRLGIGPYAARFEL  1153
Pcar_2811 YP_0067185      ------------------IKFSGDWSLFHLLNKAKITQNHNLYTLDWKLKTGQNNDAIVH  1168
plu2288 NP_929545         ------------------TAVSTLYQQWQQHQQQQTALLEQRNRLDSQLLNLSEPTVAKG  1147
plu0359 NP_927714         ------------------IRFTGPWAQLRLINSGKLTNVRQDSFDVRFTVDGGDMTYRLY  1158
plu3247 NP_930474         ------------------GDYQGNWGLIRWLAQAKAERLDESRYRLIFTAPDGLP-----  1073
PFL_6081 YP_263139        ------------------ITLDGPWAWFRLLEQSDLTATNSPDRFNLRLRVDGASISYEL  1145
PP_3091 NP_745235         ------------------FDFPGRWGLLRMNESARVDDLDGIQQRFSWNTASGRVSLVVR  1242
PSPPH_0125 YP_272435      ------------------FDYTGRWGLLRMNDSARVADLDGIQQRFSWNTAKGPVSLVVR  1279
Rmet_0633 YP_582788       ------------------YEYGGRFALLRMIGHGHIVPIDSATVQITWPAVPDTRGPLEE  1050
PA2361 NP_251051          ------------------WSYQGSWALLRMMRAHFSRQRPPNVDYTEFPLVLQLPVYAPY  1219
PA1669 NP_250360          ------------------ERNTGPWSLFRLFDLMQTEYLSGRDVMVLKADLGGLRANYLL  1152
PA0077 NP_248767          ------------------LTLEGPWAWFRLLDQSDLERGNSPDRFTLRLRIDGSSIACEL  1078
Atu4332 NP_356323         ------------------KTENGDWSPFRLFDGANIE-NQSGELLRVRFENGVQAAEFDI  1136
EvpO ABW69087            RDFPGGARRFTPADFPVDKSRMAAVGLTQITVRYRIAGQETLLQDAAQLAQAERDIQSAQ  1190
pRL120464 YP_764970       ------------------LTENGDWSPFRLFDDADLA-IQGDDLFRAKFQQSGQAAEFDV  1135
STM0285 NP_459283         ------------------LLTGGPWALFHLLDAGMVQETAVRGRQLVEYDFDGRRVVLEI  1265
Acid_0223 YP_821521       ---------------------EGLWAAFHLFQLADTQSAGAGGTQVLDWVAKSGKAGQPM  1086
YPK_0399 YP_00171915      ------------------LSFSGPWAQLRLINAGELTNVGTNSFDVRFKVDGGEMTYRIF  1154
YPK_1485 YP_00172023      ------------------ILFRGPWALLQWADQADEIRDEANGKQTLTYLMGKDRVELEV  1246
YPK_3550 YP_00172227      ------------------VSFNGPWAIFRMYDVSNPRPLGVNSRELMMVMDNVSGVFKIE  1140
YPK_2591 YP_00172132      ------------------GDYQGTWGLIRWLEQAKQKKLDEGRYQLTFTTADKQP-----  1090
XOO3038 AAW76292          ------------------VEYRGPWAWFRVMQAGRLHRSGDLD-YTASLALGGTSVRVDI  1132
RRSL_01754 EAP72360       ------------------LNEPGRFGLERLIATANRKRRPDGAFDLTWARDGISVSVSLR  1234
GSU3166 AAR36557          ------------------PQAFPDFLREFASGHRTFAAGEFPGEKDALARMGITSIRVSY  1126
ECA3432 YP_051522         ------------------LSFSGPWAMFRLVNSDQLTQVNENTFDVRFSLENGAMTYRVY  1142
VC0395_0016 WP_00203      ------------------LQIQGPWAFFRLLDQGDVVSASQTSVDFKFIVDGGEMIYRIN  985
APECO1_1770 WP_00047      ------------------IAFSGPWAQFRLFGAGQLTNVTSDTFNVRFNVDGGAMVYQVH  1152
```

```
AHA_1845 YP_856379          TDSA------------------QNPFTGGLFSQFRLPESLY-------------------  1161
BB0816 NP_887365            RAGS-----------------VRSPFHLAEMRGFSCPGRS--------------------  1201
BURPS1106A_A2840 YP_        TASS-----------------VRNPFRLPQMESFTCPPKQ--------------------  1209
BUBURPS1106A_A0140 Y       RTNG-----------------QMNPLTSGLLTHFRCPGSVG-------------------  1302
BURPS1106A_A0236 YP_        ASTGS----------------VANPLTSDVLKTFRCPGSMPMFSLADTGAPPGLPPGSPA  1347
BURPS1106A_A0717 YP_        WQALT----------------LPSPIWSGILQSFRCPS----------------------  1167
BURPS1106A_3650 YP_0        IISTSQPTG--GGDQP--QQQSLRGLRLPSSVADASAGG-----AANATAQPGSGAAAAQ  1282
Daro_2188 YP_285397         VVSSAQAQAAGNANEGGSAQRGLAGVVLPRSVAVGKAGK---------YQEPAQETAAPA  1261
c3398 NP_755273             ------------LNYLLRVEQGKGPLALLELKNFRLPGQ---------VFLTGKSMKDVE  1104
ECs0218  NP_308245          TDTE------------------DNPFSGGLFSQFGLSDTLY-------------------  1035
HH0252 NP_859783            TPNR-----------------LELLHLSRILTHFNMPQLITQQ-----------------  1184
mlr2349 NP_103715           KAAS-----------------VENPYTLEMFKKFTCPPQI--------------------  1180
MXAN_4804 YP_632965         SIDFR-------------PERTANPFFGMSGNTSKLLAIFRDPGLQPPTTIARKGECAPQ  1223
Pden_2453 YP_916236         RAAS-----------------VENPFDLSMFGAFRCPEAF--------------------  1176
Pcar_2811 YP_0067185        LKLRP--------------DRHSNVFAQGLFSQFRLPSNIF-------------------  1195
plu2288 NP_929545           TLST------------------LPSQITTHWNELRKPKSE--------------------  1169
plu0359 NP_927714           VDES------------------DNPFAGGLFSQFRLPDTLY-------------------  1181
plu3247 NP_930474           ------------LTWILRTELGEGPLALLKLRGFKLPKN---------IFVVTPGNHTTI  1112
PFL_6081 YP_263139          RASS-----------------AFNPFKSRVLSGFSLPERL--------------------  1168
PP_3091 NP_745235           NFG---------------------GVKLT---------------------DLGDVKALSA  1260
PSPPH_0125 YP_272435        NYG---------------------GVKLT---------------------DLANVKALSA  1297
Rmet_0633 YP_582788         STDAQSGKAPYEIRFLMRSEAGQGVLELLPLRGLKLPQR---------VFLVGKGGAAVD  1101
PA2361 NP_251051            SPENE-----------------ARMFLRLSLMSLGGKTPLSIQPLPVRAPQSPFATLLPA  1262
PA1669 NP_250360            LAQR-----------------TPNPFDLAVLRSFRMPVQL--------------------  1175
PA0077 NP_248767            RASS-----------------AFNPFKSRVVSGFSLPERL--------------------  1101
Atu4332 NP_356323           QFGS-----------------VLNPFKLDAIASFACPAQF--------------------  1159
EvpO ABW69087              TRQQQAQQALLTLNRPSASESLPPLDTLPGLTMMSVPERIGQCWRGDIRLARENDGIGRL  1250
pRL120464 YP_764970         QFGS-----------------VLNPFRLEALGAFSCPAQF--------------------  1158
STM0285 NP_459283          TAGR-----------------DFNPVSRELLQNFSCPARAL-------------------  1289
Acid_0223 YP_821521         LLGS------------------GKPLTVRLDLDLGAAPPLFQRGYLSRMTCVQEIAR---  1125
YPK_0399 YP_00171915        VDES------------------DNPFAGGLFSKFSLPDTLY-------------------  1177
YPK_1485 YP_00172023        SGLT-----------------HGGETISDLLRDFKCPSVNDMNGIW--------------  1275
YPK_3550 YP_00172227        LSSTM----------------KDYPLWSKALAQFSCPKNL--------------------  1164
YPK_2591 YP_00172132        ------------LQWILRTELGKGPLGLLQLRNFTLPAQ---------IFLIQHAPLAIA  1129
XOO3038 AAW76292            QPAS-----------------LRHPFAASAVQRFSCP-----------------------  1152
RRSL_01754 EAP72360         IISSPQTAGASGSDSP--QGQGLRGLRLPTSIADANASQNASTPPAPAGSRPASAPAATT  1292
GSU3166 AAR36557            QFTGS----------------QQVIRQGGAMAGATAPRMIARCW----------------  1154
ECA3432 YP_051522           TDAS------------------HNPFAGGLFSQFTLPDSLY-------------------  1165
VC0395_0016 WP_00203        AEAD------------------ANPFTERLFKSFKLSKTLY-------------------  1008
APECO1_1770 WP_00047        VDTE------------------DNPFTGGLFSLFRLPDTLY-------------------  1175

AHA_1845 YP_856379          -------------------  1161
BB0816 NP_887365            -------------------  1201
BURPS1106A_A2840 YP_        -------------------  1209
BUBURPS1106A_A0140 Y       -------------------  1302
BURPS1106A_A0236 YP_        APTSRVARDGR--------  1358
BURPS1106A_A0717 YP_        -------------------  1167
BURPS1106A_3650 YP_0        VAATAASASNAQG----AQ  1297
Daro_2188 YP_285397         PVNTGGGVQ----------  1270
c3398 NP_755273             EYGEDADE-----------  1112
ECs0218  NP_308245          -------------------  1035
HH0252 NP_859783            -------------------  1184
mlr2349 NP_103715           -------------------  1180
MXAN_4804 YP_632965         AIAADRVH-----------  1231
Pden_2453 YP_916236         -------------------  1176
Pcar_2811 YP_0067185        -------------------  1195
plu2288 NP_929545           -------------------  1169
plu0359 NP_927714           -------------------  1181
plu3247 NP_930474           SAVNDDDLITE--------  1123
PFL_6081 YP_263139          -------------------  1168
PP_3091 NP_745235           LNSRGAQ------------  1267
PSPPH_0125 YP_272435        LNATDGRTK----------  1306
Rmet_0633 YP_582788         VPSPAARNAKRGGV-----  1115
PA2361 NP_251051            TVASTGGTP----------  1271
PA1669 NP_250360            -------------------  1175
PA0077 NP_248767            -------------------  1101
Atu4332 NP_356323           -------------------  1159
EvpO ABW69087              MSELVAKQLSDKP------  1263
pRL120464 YP_764970         -------------------  1158
STM0285 NP_459283          -------------------  1289
Acid_0223 YP_821521         -------------------  1125
YPK_0399 YP_00171915        -------------------  1177
YPK_1485 YP_00172023        -------------------  1275
YPK_3550 YP_00172227        -------------------  1164
YPK_2591 YP_00172132        DMSDDEDMAED--------  1140
XOO3038 AAW76292            -------------------  1152
RRSL_01754 EAP72360         QAATTARSTVAQEQGGLAQ  1311
GSU3166 AAR36557            -------------------  1154
```

ECA3432 YP_051522	- - - - - - - - - - - - - - - - - - -	1165
VC0395_0016 WP_00203	- - - - - - - - - - - - - - - - - - -	1008
APECO1_1770 WP_00047	- - - - - - - - - - - - - - - - - - -	1175
